# Cooperative intra- and intermolecular hydrogen bonding in scaffolded squaramide arrays†

**DOI:** 10.1039/d4sc04337e

**Published:** 2024-09-19

**Authors:** Luis Martínez-Crespo, George F. S. Whitehead, Iñigo J. Vitórica-Yrezábal, Simon J. Webb

**Affiliations:** a Department of Chemistry, University of Manchester Oxford Road Manchester M13 9PL UK S.Webb@manchester.ac.uk luis.martinez@uib.es; b Manchester Institute of Biotechnology, University of Manchester 131 Princess St Manchester M1 7DN UK

## Abstract

The structural, self-assembly and binding properties of oligo-(phenylene-ethynylene) (OPE) rigid rods linked to squaramides (SQs) have been studied and correlated with rod length. In the solid-state, OPE–SQ conjugates form indefinite arrays of head-to-tail hydrogen bonded SQs, arrays that include both intra- and intermolecular hydrogen bonds. In dichloromethane solution, intramolecularly hydrogen bonded SQ chains show cooperative polarisation, an effect that increases with OPE–SQ length. Appending powerful hydrogen bonding groups to the OPE–SQ termini further increases this intramolecular polarisation. Greater end-to-end polarisation leads to stronger intermolecular interactions, with longer OPE–SQs showing stronger hydrogen bonding to DMSO as well as stronger self-association. These studies show how cooperative hydrogen bond polarisation in a hydrogen bonded array can be strengthened and how this polarisation can continue intermolecularly.

## Abstract 2

Aromatic rods functionalised with squaramides form intramolecularly hydrogen-bonded arrays that exhibit cooperative polarisation, an effect that increases with rod length. Greater polarisation in turn produces stronger intermolecular interactions.

## Graphical abstract

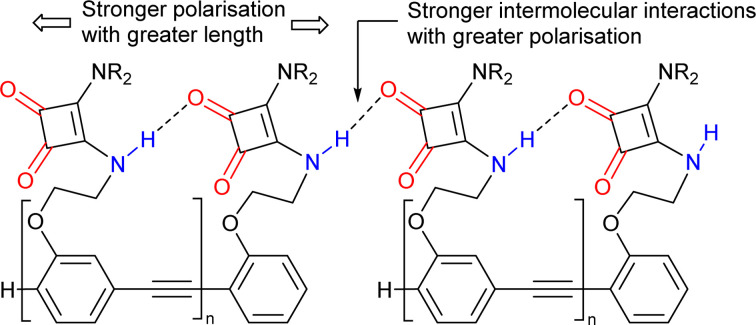

## Introduction

Hydrogen bonding (H-bonding) networks play crucial roles in both natural and artificial systems. They are central to the formation of protein secondary structures, such as α-helices and β-sheets, and help define the way in which secondary structures interact to give tertiary folding. In artificial systems, intramolecular H-bonding networks have been used to develop molecular communication devices,^1–3^ whereas intermolecular H-bonding networks can lead to the formation of supramolecular polymers,^4^ H-bonded organic frameworks (HOFs),^5,6^ co-crystals^7^ or responsive gels.^8,9^

**Fig. 1:**
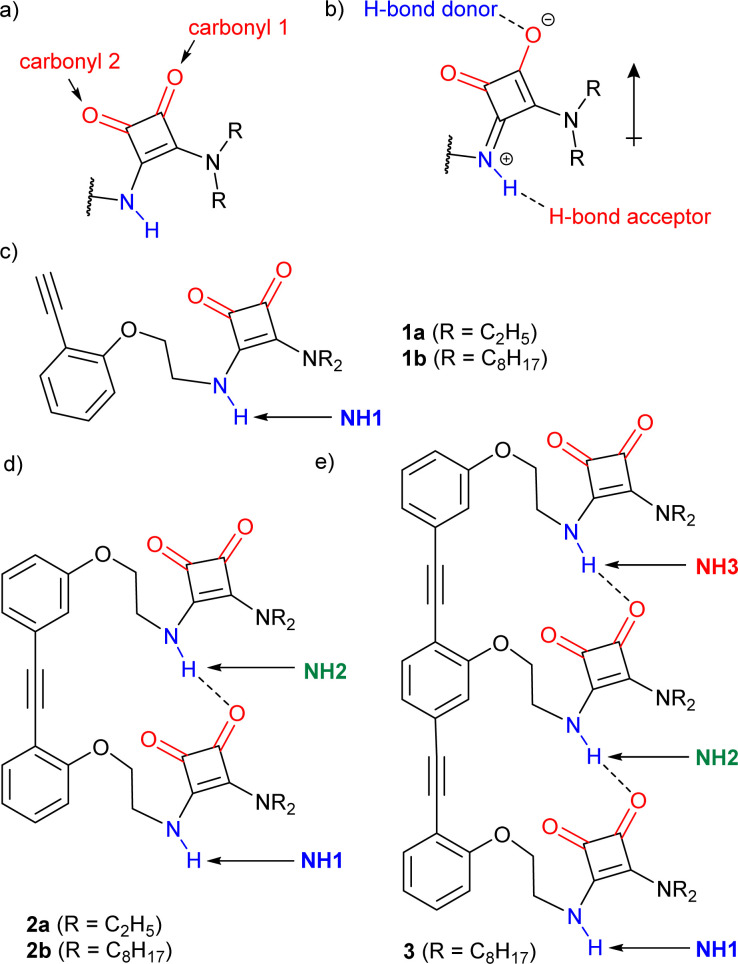
(a) A secondary–tertiary squaramide (SQ) motif. (b) Resonance form showing how H-bonding promotes polarisation in a SQ. (c–e) Structure of the OPE–SQ derivatives reported in this study: (c) monomers, (d) dimers, (e) trimer. H-bond donor and acceptor groups are labelled in blue and red, respectively. Different NH protons are labelled in each structure.

Compounds with functional groups possessing both H-bond donor and acceptor properties, such as squaramides (SQs, Fig. 1a), can form extended head-to-tail chains where the average strength per H-bond is greater than the strength of each H-bond in isolation, an effect called “H-bond cooperativity”.^10–12^ This effect has been observed for water, alcohols, amides, ureas and squaramides.^10–14^ The “H-bond cooperativity” observed in such systems is proposed to arise from polarisation of these functional groups when they interact with another strong dipole (shown for a squaramide, Fig. 1b), which in turn favours further H-bonding. This cooperative formation of successive intermolecular H-bonds has been invoked to explain why alcohols are more polar solvents than might be expected from their individual H-bonding properties^15,16^ or why certain supramolecular polymerisation processes start with a slow nucleation stage followed by a faster elongation stage.^17,18^

This effect has also been studied in intramolecular H-bonding networks. For example, cooperative polarisation between multiple aligned urea and thiourea units in a molecule has been proposed to increase the catalytic activity of the terminal NH groups.^19–22^ Recently, Cockroft, Hunter and co-workers have quantified cooperativity, both in intramolecular H-bonded chains formed by OH groups^23,24^ and when amides interact with either donors or acceptors.^25,26^ These studies confirm that the formation of successive intramolecular H-bonds can cooperatively polarise a terminal H-bond donor or acceptor, strengthening its interactions with molecules bearing a complementary motif. The magnitude of this cooperative polarisation increases with the number of H-bonding groups involved.^23^ Increased polarisation of terminal H-bonding groups should in turn also favour intermolecular interactions between molecules of the same kind. Indeed, formation of an intramolecular H-bonded array should favour intermolecular extension of the array and further cooperative polarisation. It might be anticipated that the strength of self-aggregation will depend on the length of the intramolecular H-bonded arrays in the aggregating molecules.

In previous work we showed trimeric oligo-(phenylene-ethynylene) (OPE) rigid rods linked to squaramide (SQ) arrays could relay conformational change over their 1.8 nm length, specifically by inverting the SQ array direction.^1^ These studies suggested that the internal H-bond network of each SQ array was end-to-end polarised and that the strength of this polarisation may change for different terminal H-bonding groups. However to understand these effects it was clear that simpler OPE–SQ conjugates would be required. Comparison between analogous conjugates of different length could also provide insight into how SQ array size affects end-to-end polarisation.

In this study, rod-like OPE–SQ conjugates of different lengths were designed and synthesised (Fig. 1c–e). Each example contains an intramolecularly H-bonded array formed by SQ units of the same type. Their propensity to form intermolecular H-bonds, both to DMSO and to each other, has been measured and correlated with their length.

## Results and discussion

### Design and synthesis

Compounds 1a–b, 2a–b and 3 (Fig. 1c–e) were designed to retain key structural motifs from previous trimeric OPE–SQ conjugates but without the capping groups present in these trimers (*e.g.* in 4 and 5, shown in Fig. 5).^1^ The SQs are held in close proximity but spaced apart by the rigid OPE backbone, with the flexibility of each ethylene link allowing optimisation of SQ–SQ H-bond distances. In contrast to previously reported conjugates,^1^1a–b, 2a–b and 3 have the same repeat units in the SQ array, which allows the effect of SQ array length to be studied independently of polarisation induced by non-SQ units. These compounds were prepared by modifying conditions developed for the synthesis of previous OPE–SQ conjugates.^1^ The symmetry in 1a–b, 2a–b and 3, however, made them directly accessible by condensation of monomeric, dimeric or trimeric amino-OPEs with the appropriate squarate esters (see Section 2 of the ESI†).

The rigidity of the OPE scaffold is designed to preorganise the SQs so they form a chain linked by intramolecular H-bonds. The aryl–aryl distance in the OPE scaffold was chosen to be commensurate with typical distances between H-bonded SQs.^14^ Much like an amide, each SQ has H-bond donor and acceptor capability (labelled in blue and red respectively in Fig. 1) and H-bonds to an SQ unit can increase its polarisation (Fig. 1b). The flexibility of the ethylene linker permits the SQs to adopt geometries that maximise the strength of these H-bonds. This flexibility also allows the SQ units to adopt opposite orientations relative to the OPE rod. These orientations can be arbitrarily defined as *parallel* (the squaramide and phenylethynyl oxygens point in same direction) or *antiparallel* (with the opposite relative orientation).^1^

### Conformational and self-assembly properties of 1a, 2a, 2b and 3 in the solid-state

**Fig. 2:**
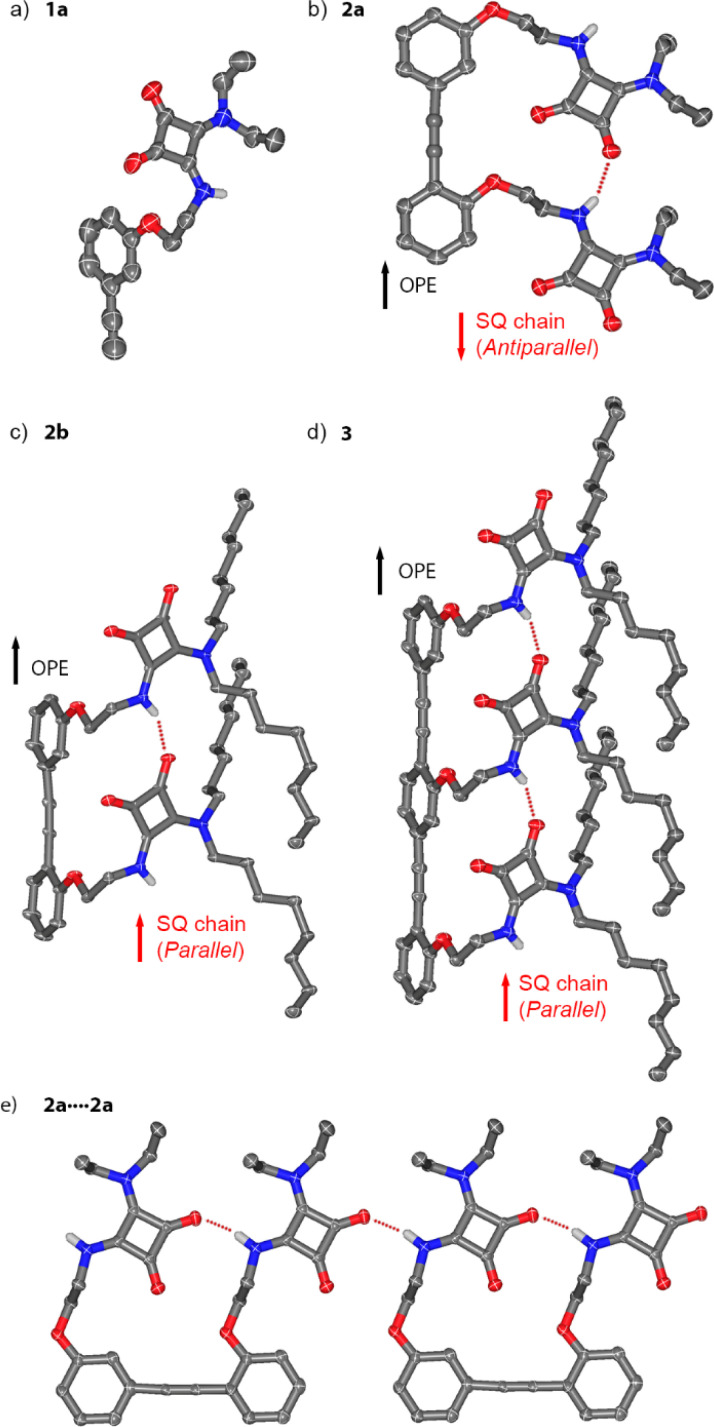
Crystal structures of (a) 1a, (b) 2a, (c) 2b and (d) 3. The relative orientations of the OPE rigid rod (black arrows) and the squaramide chain (red arrows) reveal an *antiparallel* conformation for 2a and a *parallel* conformation of 2b and 3. (e) Portion of the structure of 2a showing the identical geometry of inter- and intramolecular hydrogen bonds. Carbon atoms are in grey, oxygen in red, nitrogen in blue and hydrogen in white. All hydrogen atoms, except those involved in hydrogen bonds, are omitted for clarity. Selected hydrogen bonding interactions are indicated by red dashed lines.

Crystals suitable for single crystal X-ray diffraction were obtained for compounds 1a, 2a, 2b and 3 (Fig. 2). Crystals of 1a and 2a were obtained from solutions in dichloromethane and DMSO–MeOH, respectively. Crystals of 2b and 3 were obtained from acetonitrile and acetone solutions respectively.

The monosquaramide 1a (Fig. 2a) can only form intermolecular H-bonds. In the solid-state it forms head-to-tail arrays linked by H-bonds from the NH to an adjacent C=O group (carbonyl 2, N⋯O distance of 2.8128(14) Å, see the ESI, Fig. S40†).^27^ The planes of adjacent SQs alternate at an angle of 122.89(10)° (Fig. S40b†). CH–π interactions are observed between SQs and the phenylethynyl moieties of neighbouring 1a (Fig. S40c†).

The structure of dimeric rigid rod 2a with ethyl substituents (Fig. 2b and e) shows an intramolecular H-bond between the NH of one SQ unit and the C=O (carbonyl 1) of the neighbouring SQ. The distance between the two SQ–NHs matches the distance between the centroids of the two aromatic rings of the diarylacetylene moiety (6.8442(5) Å). In addition, intermolecular H-bonds are present (Fig. 2e), with geometries and distances between SQ units that exactly match those in the intramolecular SQ array. This produces crystallographic disorder, where crystallographic translation of *ca*. 6.9 Å (half the dimer length) along the “alkyne axis” of the OPE gives the same locations for most atoms. When solving the structure, the electron density at first appears to be polymeric, with the asymmetric unit in the crystal defined by a single phenylene-ethynylene unit linked to a SQ. However, free refining the occupancies of the alkyne moieties in the models for 2a and 2b gave close to 50% occupancy for these carbon atoms (see Section 5.1 in the ESI and Fig. S41a†). These partial occupancies were constrained to 50% when refining the model. As a result of the crystallographic translation, intermolecular and intramolecular H-bond distances cannot be distinguished in the indefinitely long head-to-tail SQ chains (average N⋯O distance of 2.888(2) Å). The relative orientations of the C=O dipoles in the SQ array are opposite to the C–O dipoles in the phenylethynyl, which has been arbitrarily defined as an *antiparallel* orientation.^1^ Planes defined by the phenyl rings and the SQ rings are almost parallel (7.36(11)°, Fig. S41b†), which facilitates intermolecular π-stacking that places the SQ rings 3.474(2) Å above the OPE rings. Each conjugate adopts the opposite direction to its neighbour, a relative orientation commonly observed with SQs^14,28–30^ and one that would be favoured by the macrodipoles in these rod-like molecules (Fig. S41c†).^19,31,32^

Much like 2a, the crystal structures of *n*-octyl substituted 2b and 3 show the molecules form head-to-tail SQ chains of indefinite length that contain indistinguishable inter- and intramolecular H-bonds (N⋯O 2.887(4) Å for 2b, N⋯O 2.871(3) Å for 3, Fig. 2c and d). Crystallographic disorder for 2b is similar to that for 2a, whereas disorder in 3 is slightly different, since the crystallographic translation (*ca*. 6.9 Å) is now a third of the OPE trimer length. Free refining the occupancies of the alkyne moiety in 3 gave approx. 65% occupancy (see Section 5.1 in the ESI and Fig. S43a†), consistent with a trimer. These partial alkyne occupancies were constrained to 67% when refining the model. Distances between repeating SQ–NH units are similar to those between OPE phenyl centroids (6.9824(4) Å and 6.9566(4) Å, respectively). The SQ and OPE rings in 2b and 3 now define perpendicular planes (85.04(13)° and 84.89(12)°, respectively, see the ESI†). Instead of the OPE/SQ π-stacking seen in 2a, SQ/SQ π-stacking is present (3.515(3) Å for 2b, 3.488(3) Å for 3) with stacked arrays adopting opposing macrodipoles.

The SQ array in 2b (and in 3) adopts a *parallel* orientation, the opposite of that observed for ethyl-substituted analogue 2a. This suggests that *parallel* and *antiparallel* conformers are close in energy, which is consistent with previously reported switchable interconversion in related OPE–SQs in solution.^1^ Which orientation predominates in the solid state is likely to be due to a combination of factors. The antiparallel and parallel conformations have significantly different molecular shapes (flat and folded shapes respectively) and the ease of crystallising each will be different. The spatial requirements of the different substituents on the squaramides (*e.g.* ethyl in 2a and *n*-octyl in 2b) are also likely to play a role.

These solid-state structures show how the OPE scaffold preorganises the SQs and facilitates the formation of the intramolecular SQ–SQ array. Each array then extends indefinitely through intermolecular SQ–SQ interactions between complementary “sticky” ends on the OPE–SQ conjugates (*e.g.* NH1 and carbonyl 1). These intermolecular SQ–SQ interactions, which are geometrically different to those observed for monomer 1a, have almost identical geometry to the intramolecular SQ–SQ interactions (Fig. 2e). The absence of capping groups or bulky terminal groups on the OPEs allows extension of the SQ array without significant distortion of array geometry, which in turn permits crystallographic translation in the solid-state. The near-identical SQ–SQ geometry both within and between molecules suggests that crystallisation favours solid-state structures that include long “polymeric” arrays of SQs, perhaps to maximise cooperative polarisation. The solid-state structures of 2b and 3 therefore likely reflect both the effect of scaffolding by each OPE and the geometric preferences of H-bonded SQs.

In solution, the formation of long arrays of SQs involving multiple OPE–SQ conjugates might also be enthalpically favourable. However, how the strength of these intermolecular SQ–SQ interactions depends on scaffold length is unclear, although stronger intramolecular cooperative polarisation might be expected to strengthen these H-bonds.

### Intramolecular hydrogen bonds in 2b and 3 in solution

**Fig. 3:**
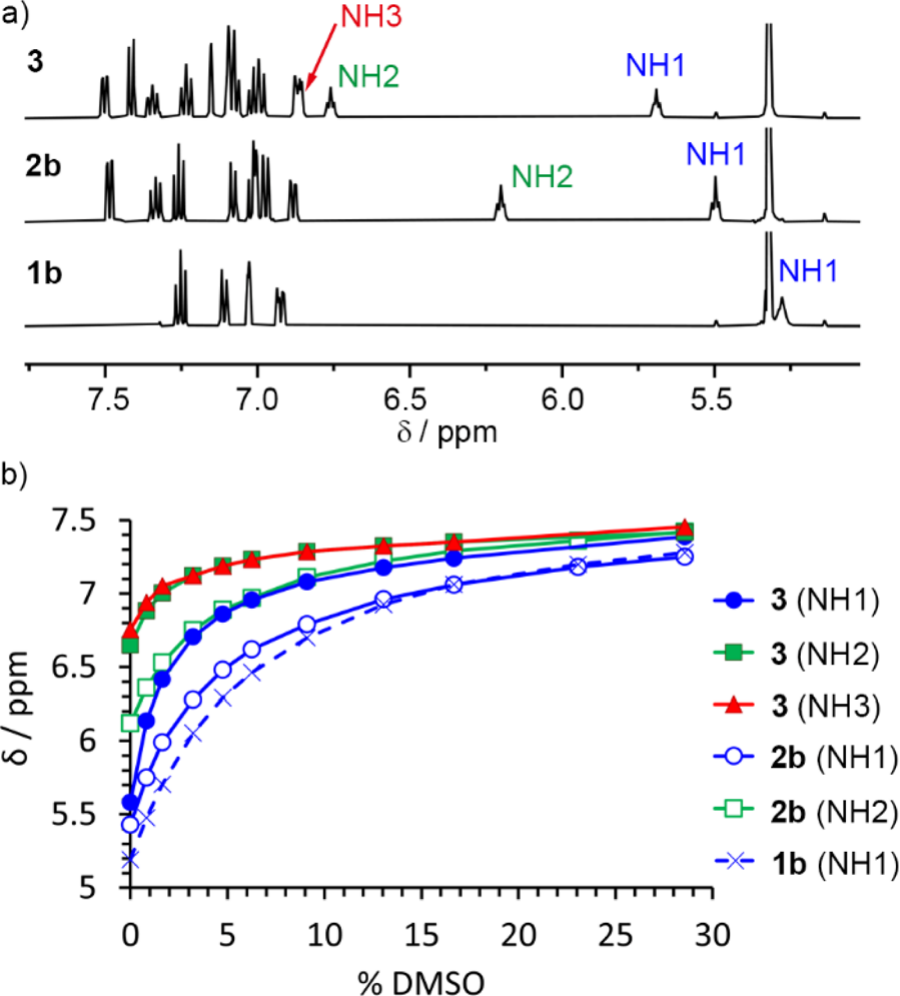
(a) Partial ^1^H NMR spectra of compounds 1b, 2b and 3 (each 2.5 mM in CD_2_Cl_2_, 500 MHz, 298 K). NH1 is exposed to the solvent in all cases (*δ* = 5.3 to 5.7 ppm, *parallel* conformation). The chemical shifts of NH2 suggest that the intramolecular H-bonding network in 3 is stronger than in 2b. (b) Variation of the chemical shift of the NH signals of compounds 1b, 2b and 3 during their titration with DMSO-*d*_6_.

To study how polarisation depends on OPE–SQ length, the NH resonances of 1b, 2b and 3 were analysed in CD_2_Cl_2_ solution using NMR spectroscopy (Fig. 3). Compounds 1a and 2a, which lack the *n*-octyl solubilising chains, were excluded from these studies due to low solubility in CD_2_Cl_2_. In the solid-state, 2b and 3 showed a *parallel* orientation of the SQ chain relative to the OPE; NMR spectroscopy confirmed that a *parallel* orientation was also adopted in solution. The chemical shifts of the terminal NH1 signals (5.5 ppm for 2b and 5.7 ppm for 3; each identified as described in the ESI, Section S3.1†) are close to that of 1b (5.3 ppm) but the chemical shifts of the other NHs of 2b and 3 are at lower field (6.2 ppm for NH2 of 2b, and 6.7 and 6.8 ppm for NH2 and NH3 of 3, respectively, Fig. 3a). This agrees with the formation of intramolecular H-bonding networks of *parallel* orientation. Moreover, the chemical shifts of the internal NHs in 3 are noticeably further downfield than that of the internal NH in 2b, suggesting that internal H-bonds have strengthened as the SQ array has increased in length.

The OPE scaffold is suggested to play an important role in the formation of SQ arrays by preorganising the SQs, decreasing the entropic penalty for the formation of intramolecular SQ–SQ H-bonds. The resulting polarisation (Fig. 1b) should then support the formation of intermolecular SQ arrays, which would be consistent with reports of intermolecular cooperativity in SQ head-to-tail networks.^29,33–37^

Titration of DMSO-*d*_6_ into CD_2_Cl_2_ solutions of 1b, 2b and 3 was used to confirm that longer SQ arrays produce greater polarisation of exposed NH1 protons, as it should produce correspondingly stronger interactions with added H-bond acceptors. The NH1 signal of compound 3 was substantially more affected by the progressive addition of DMSO-*d*_6_ than signals NH2 and NH3 (Fig. 3b). Similarly, for 2b, DMSO-*d*_6_ addition had a greater effect on NH1 than on NH2 (Fig. 3b). These observations agree with a *parallel* conformation in both compounds. Fitting these data to 1 : 1 binding models afforded binding constants of 11, 15 and 35 M^−1^ for 1b, 2b and 3, respectively (first three entries in Table 1 and Fig. S12†), confirming that the NH1 of 3 binds DMSO-*d*_6_ more strongly than the NH1 of 2b and 1b. This observation is consistent with greater end-to-end polarisation of the longer SQ array in 3 due to intramolecular cooperativity.^23,26^

**Table 1:** Association constants for binding to DMSO-*d*_6_ (*K*_a_(DMSO)) and the dimerisation constants (*K*_dim_) determined from dilution experiments performed in CD_2_Cl_2_

Compound	*K*_a_(DMSO)^a^/M^−1^	*K*_dim_ a/M^−1^
1b	11	0.3
2b	15	3.6
3	35	19.9
4	46	3.6
5	11	0.8

a Data fitted with https://www.app.supramolecular.org/bindfit/.^38^

^1^H NMR spectroscopy and DMSO-*d*_6_ binding studies were also performed in (CD_3_)_2_CO (Fig. S23–S39†), a better H-bond acceptor than CD_2_Cl_2_. The conformations of 1b, 2b, and 3 appear to be the same in (CD_3_)_2_CO as in CD_2_Cl_2_; higher chemical shifts of NH2 compared to NH1 are consistent with a parallel intramolecular H-bond network that has NH1 exposed and NH2 bound. Similarly, NH3 of 3 is also involved in the intramolecular H-bond network. In keeping with the H-bond acceptor properties of this solvent, the binding constants for 1b, 2b, 3 and 4 to DMSO-*d*_6_ are all approximately halved (Fig. S34†).

### Aggregation of 1b, 2b and 3 in CD_2_Cl_2_ solution

The differing contributions of inter- and intramolecular H-bonding to polarisation in 1b, 2b and 3 became clear when preparing solutions with the same concentration of SQ units (60 mM); this corresponds to a decreasing concentration of each OPE–SQ (60 mM 1b, 30 mM 2b and 20 mM 3). The chemical shifts of the exposed NH1 protons (*δ*(NH1) = 5.39, 5.71 and 5.89 ppm respectively) increased with increasing OPE length at these concentrations of 1b, 2b and 3, even though the amount of NH1 available to form intermolecular H-bonds decreased. This correlation of *δ*(NH1) with OPE length (1b < 2b < 3) is consistent with increased end-to-end polarisation in longer SQ arrays, but does not provide information on how increasing polarisation has affected aggregation.

**Fig. 4:**
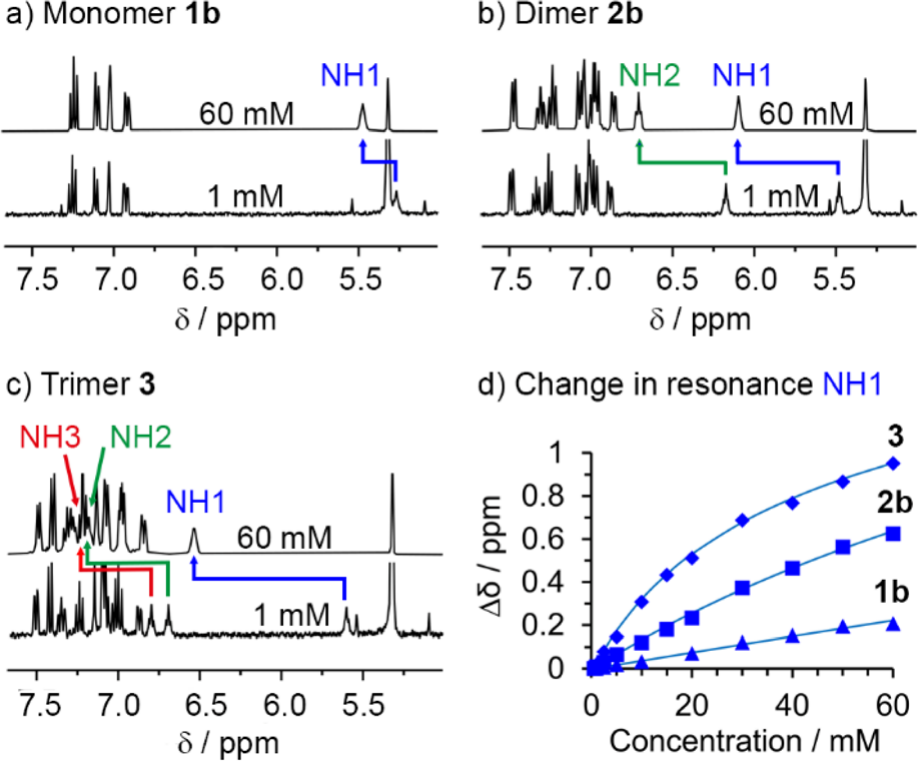
^1^H NMR dilution experiments (CD_2_Cl_2_, 400 MHz, 298 K) for (a) 1b, (b) 2b and (c) 3. (d) Changes in chemical shift observed for NH1 with increasing concentration (0.5–60 mM). Calculated curves resulting from the fitting of the experimental data to a dimerisation model are shown.

To quantify the relationship between OPE–SQ length and aggregation, the effect of changing the concentration of 1b, 2b and 3 on their respective ^1^H NMR spectra was studied. For each compound, all the NH signals appeared further downfield at 60 mM than at 1 mM (Fig. 4). The resonance of solvent-exposed NH1 experiences the greatest downfield movement upon increasing OPE–SQ concentration, an effect that is stronger in the order 1b < 2b < 3. This suggests that aggregation is stronger when the number of SQ units in the relay is increased (Fig. 4). The resonances of the other NHs also shift downfield as OPE–SQ concentration increases, suggesting that H-bonding to NH1 causes additional polarisation that is relayed along the SQ array. The sensitivity of terminal NH resonances to concentration has been used to quantify the head-to-tail aggregation of peptides in solution and the same methodology can be applied here.^39^ Both isodesmic self-association (with equal *K* values) and dimerisation models have been used to analyse aggregation processes,^40,41^ each providing related self-association constants.^42^ The changes in chemical shift of NH1 in 1b, 2b and 3 were fitted to a dimerisation model.^43^ Dimerisation constants (*K*_dim_) were calculated for each compound by iterative curve fitting, which confirmed stronger aggregation as OPE length increased (entries 1–3 in Table 1).

To support these observations and to confirm that the internal H-bonds remain intact during self-aggregation, we also performed variable temperature (VT) ^1^H NMR experiments from 258 to 298 K at different concentrations of 1b, 2b and 3. The effect of temperature on each NH resonance can be quantified by calculating the corresponding temperature coefficient (Δ*δ*/Δ*T*, Table 2 and ESI, Table S1†). Previous studies of short peptides in CDCl_3_ suggest that temperature coefficients close to −3 ppb K^−1^ are indicative of NHs either completely exposed to this solvent or completely shielded, while values substantially higher (in absolute value) suggest the formation of H-bonds of intermediate strength.^30,44,45^

**Table 2:** Δ*δ*/Δ*T* coefficients calculated for 1b, 2b and 3 between 0.1 and 20 mM in CD_2_Cl_2_

Compound	Concentration/mM	Δ*δ*/Δ*T* (ppb K^−1^)		
		NH1	NH2	NH3
1b	0.5	−2		
	20	−4		
2b	0.5	−2	−8	
	20	−12	−15	
3	0.1	−3	−10	−9
	20	−17	−12	−13

Low Δ*δ*/Δ*T* values were observed for the monomer 1b, where NH1 is always exposed to solvent (CD_2_Cl_2_). The low self-association constant of 1b means its NH shows little dependence of Δ*δ*/Δ*T* on concentration (entries 1–2, Table 2). Like 1b, the NH1 resonances of 2b and 3 at low concentrations (≤0.5 mM) showed low Δ*δ*/Δ*T* values, which suggests little formation of intermolecular H-bonds. The values for NH2 and NH3 at this concentration are significantly greater, consistent with intramolecular H-bonds of intermediate strength. Increasing the concentration of 2b and 3 to 20 mM produced little change in the Δ*δ*/Δ*T* coefficients for the intramolecularly H-bonded NHs, suggesting aggregation did not significantly disrupt the intramolecular array (Table 2 and ESI, Table S1†).^45,46^ However more substantial increases in Δ*δ*/Δ*T* for NH1 were observed, consistent with more extensive intermolecular H-bonding at higher concentrations.

### Effect of OPE–SQ capping on polarisation and aggregation

**Fig. 5:**
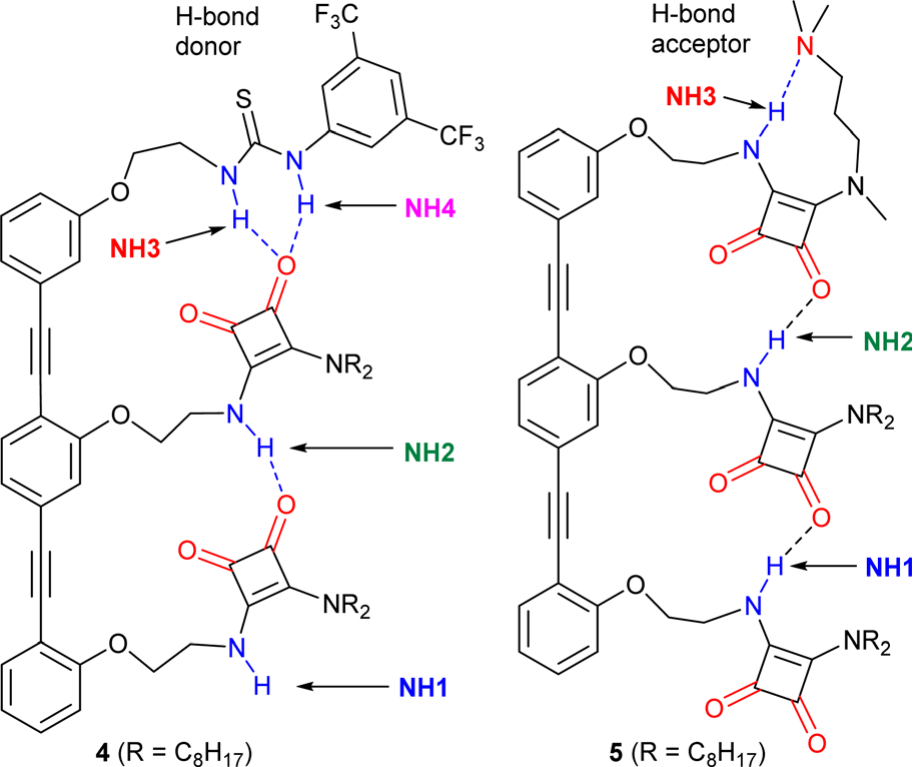
Structures of previously reported capped OPE–SQ derivatives 4 and 5 in *parallel* and *antiparallel* conformations respectively.^1^ The H-bond donor and acceptor groups are labelled in blue and red, respectively.

Trimers 4 and 5 (Fig. 5), reported in a previous study,^1^ offer an insightful contrast to the behaviour of 3. Each capping group controls the orientation of the array; the thiourea in 4 produces a *parallel* orientation whereas the amine in 5 produces an *antiparallel* orientation.^1^ A further effect of adding these groups, a strong H-bond donor in the case of 4 and strong H-bond acceptor in the case of 5, is to cap one of the “sticky” ends of the SQ array. It could then be expected that OPE–SQ aggregation would be suppressed, similar to the effect of the *tert*-butyl group in the systems reported by Hunter.^23^

The crystal structures of 4 and 5 show the effect of capping on intermolecular interactions.^1^ OPE–SQ 4 is the only crystallised conjugate that does not self-assemble in a head-to-tail fashion, which reflects the very poor H-bond acceptor character of the thiourea cap. Instead, terminal NH1 forms an intermolecular H-bond with an SQ carbonyl not involved in the intramolecular H-bonding network (carbonyl 2, shown in Fig. 1a), leading to zig-zag chains (Fig. S44†). In contrast, the solid-state structure of 5 reveals the underlying strength of head-to-tail intermolecular aggregation. In CD_2_Cl_2_ solution, 5 forms an intramolecular NH3⋯N(amine) hydrogen bond in a nine-membered ring (Fig. 5), so does not have an NH able to interact intermolecularly.^1^ However in the solid-state this nine-membered ring is lost and replaced with an intermolecular end-to-end hydrogen bond, which in combination with favourable packing interactions with the *n*-octyl chains is sufficient to overcome the strong intramolecular NH_3_⋯N H-bond observed in solution (Fig. S45†). The head-to-tail SQ network formed by 5 in the solid-state may indicate that the enthalpic gain arising from strong cooperative polarisation along SQ chains that span molecules helps to overcome the relatively strong NH3⋯N(amine) H-bond.

The exposure of the NH1 of 4 to solvent is supported by a DMSO-*d*_6_ titration, which afforded a binding constant of 43 M^−1^ (Table 1, Fig. S10 and S12†). This value, together with the fact that compound 4 shows the highest chemical shift for the interior NH2 of all compounds studied (7.2 ppm, Fig. S5†), suggests that the strong H-bond donor character of the thiourea group in 4 may further polarise the NHs in the array and produce a more robust intramolecular H-bonding network. In contrast, titration of 5 with the H-bond acceptor DMSO-*d*_*6*_ afforded a binding constant of 11 M^−1^ (Table 1 and Fig. S11, S12†), which is lower than those obtained with 3 and 4. This value is consistent with all the NH H-bond donors being involved in intramolecular H-bonds. Nonetheless, intramolecular cooperative NH polarisation is still present, reflected by the high chemical shift of NH2 in compound 5 (7.0 ppm, Fig. S6†) compared to NH2 of 2b (6.2 ppm, Fig. 3a).

Despite increased polarisation in 4 and 5, the weakening of aggregation caused by capping the “sticky” ends is clear in the dilution data. Both 4 and 5 showed significantly lower *K*_dim_ values compared to uncapped trimer 3 (Table 1). The SQ NHs in 4 are even more polarised than in 3 but the H-bonding network is capped with a thiourea group, which is a poor H-bond acceptor. Thus, aggregation of 4 is much weaker than that of 3. On the other hand, OPE–SQ 5 offers an H-bond acceptor (the terminal SQ-carbonyl) but no H-bond donor since all NHs are involved in intramolecular H-bonds. Accordingly, 5 also shows a much lower tendency to self-aggregate than 3.

## Conclusions

A synthetic strategy that produced incrementally longer OPE–SQ conjugates has allowed both inter- and intramolecular cooperative H-bond polarisation to be probed as a function of molecular length.

Each OPE scaffold preorganises the attached SQ units and facilitates the formation of a hydrogen bonded SQ array. Structural matching of the OPE repeat distance with the distance between pairs of hydrogen bonded SQs allows the intramolecular SQ arrays to form without distortion. Solid-state structures also show the SQ arrays continue between OPE–SQ conjugates. There is a very close match between the geometry and distances of inter- and intramolecular H-bonds, which produces positional disorder in the crystals, with lateral displacement by either a half (for the dimer) or a third (for the trimer) of an OPE.

^1^H NMR spectroscopy, binding and dilution studies confirmed that longer OPE–SQ arrays show greater intramolecular cooperative polarisation. This in turn made interactions with other OPE–SQs stronger, which is proposed to extend cooperative polarisation intermolecularly as the SQ arrays bridge between molecules.

These OPE–SQ conjugates may become a valuable new communication motif for molecular devices; amine-capped 5 has been shown to act as a 1.8 nanometre long switchable conformational relay.^1^ OPE–SQs possess several useful characteristics for communication applications, including defined length, rigidity, structural matching between rod and array, interconvertible opposing conformational states, and lengthwise cooperative polarisation. The latter effect may improve the fidelity of information transfer by decreasing the frequency of conformational inversions;^47^ such inversions would break the chain of polarised hydrogen bonds. The structural features that permit SQ arrays to continue intermolecularly between OPE–SQs could offer a path towards much longer information relays that span tens of nanometres.

End-to-end relays of hydrogen bond polarisation have wider significance, for example indicating how remote electrostatic changes might be electronically communicated to the binding site of proteins.^48^ Switchable changes in cooperative polarisation may also provide a method of transferring information (in the place of conformational change).^3,49^ For example, a mechanism to mask/unmask a strong H-bond acceptor or donor at one terminus might act as an electronic signal that is communicated along the array to the far end.

## Author contributions

L. M.-C. and S. J. W. conceived the idea, acquired the funding, administered the project, designed the experiments, analysed the data and wrote the manuscript. L. M.-C. carried out the experimental work. G. F. S. W. and I. J. V.-Y. acquired and analysed X-ray diffraction data. S. J. W. provided resources and supervision.

## Conflicts of interest

There are no conflicts to declare.

## Supplementary Material

SC-015-D4SC04337E-s001

SC-015-D4SC04337E-s002

## Data Availability

The data supporting this article have been included as part of the ESI.† CCDC 2362335, 2362336, 2240191 and 2362337 contain the crystallographic data for 1a, 2a, 2b, and 3, respectively.
