# Supplementary material for: Cooperative intra- and intermolecular hydrogen bonding in scaffolded squaramide arrays
Source: Chem Sci. 2024 Sep 19;15(41):17120–7. doi: 10.1039/d4sc04337e (PMC11428187; doi:10.1039/d4sc04337e)
Supplement: SC-015-D4SC04337E-s001 [file SC-015-D4SC04337E-s001.pdf]

Supplementary Information (SI) for Chemical Science.
This journal is © The Royal Society of Chemistry 2024

Electronic Supplementary Information

Cooperative intra- and intermolecular hydrogen bonding in scaffolded squaramide arrays

Luis Martínez-Crespo,[1,2]* George F. S. Whitehead,[1] Iñigo J. Vitórica-Yrezábal,[1] and Simon J. Webb[1]*

[1] Department of Chemistry, University of Manchester, Oxford Road, Manchester M13 9PL, UK.

[2] Manchester Institute of Biotechnology, University of Manchester, 131 Princess St, Manchester M1 7DN, UK.

Table of contents

1. General experimental information 3
1.1. Abbreviations 4
2. Synthetic procedures 5
3. ^{1}H NMR experiments in CD_2Cl_2 for compounds **1b**, **2b**, **3**, **4** and **5** 10
3.1. Assignment of the ^{1}H NMR spectra in CD_2Cl_2 10
3.2. Titrations with DMSO-d_6 in CD_2Cl_2 16
3.3. Dilution experiments in CD_2Cl_2 22
3.4. VT experiments in CD_2Cl_2 27
4. ^{1}H NMR experiments in $(CD_3)_2CO$ for compounds **1b**, **2b**, **3**, **4** and **5** 34
4.1. Assignment of the ^{1}H NMR spectra in $(CD_3)_2CO$ 34
4.2. Titrations with DMSO-d_6 in $(CD_3)_2CO$ 41
4.3. VT experiments in $(CD_3)_2CO$ 47
5. X-ray crystal structures 49
5.1. Crystallographic data 49
5.2. Crystal structure representations 51
6. NMR spectra (complete) 57
7. References 74

1. General experimental information

All reagents and solvents were obtained from Sigma-Aldrich, Fluorochem, Alfa Aesar and VWR, and were used without further purification unless otherwise stated. Flash chromatography was performed on silica gel (Merck 60H, 40-60 nm, 230–300 mesh). Analytical thin layer chromatography (TLC) was performed on Macherey Nagel alugram SIL G/UV254 TLC sheets and TLC plates were visualised by UV irradiation (254 nm).

NMR spectra were recorded in deuterated solvents using either Brüker AVANCE 400 MHz or Brüker AVANCE 500 MHz spectrometers. Chemical shifts are quoted in parts per million (ppm) and coupling constants (*J*) are quoted in Hz to the nearest 0.5 Hz. ^{1}H NMR spectra were referenced to the residual deuterated solvent peak ($CHDCl_2$: 5.32, DMSO-d_5: 2.50, acetone-d_5: 2.05), unless stated otherwise.[S1] ^{13}C NMR spectra were referenced to the resonance of the solvent (CD_2Cl_2: 53.8, DMSO-d_5: 39.52, $(CD_3)_2CO$: 2.05 ppm).[S1]

High-resolution mass spectra (HRMS) were recorded by staff at the University of Manchester, on a Thermo Q-Exactive and are accurate to ±0.001 Da.

1.1. Abbreviations

- AcOEt: ethyl acetate
- Boc: *tert*-butyloxycarbonyl
- COSY: Correlated Spectroscopy
- DCM: dichloromethane
- DMF: dimethylformamide
- DIPA: *N,N*-diisopropylamine
- DIPEA: *N,N*-diisopropylethylamine
- DMSO: dimethylsulfoxide
- EtOH: ethanol
- Et_2O: diethyl ether
- EXSY: Exchange spectroscopy
- HMBC: Heteronuclear Multiple Bond Correlation
- HRMS: High-resolution mass spectrometry
- HSQC: Heteronuclear Single Quantum Coherence
- MeOH: methanol
- NMR: Nuclear magnetic resonance
- NOESY: Nuclear Overhauser Effect Spectroscopy
- ppm: parts per million
- SQ: squaramide
- TEA: triethylamine
- VT: Variable temperature

2. Synthetic procedures

Compounds **4**, **5** and **15** and precursors **6**-**11** were prepared as reported previously.[S2]
Compounds **1a-b**, **2a-b** and **3** were prepared following Scheme S1.

Scheme S1. Synthesis of compounds **1a-b, 2a-b** and **3**.

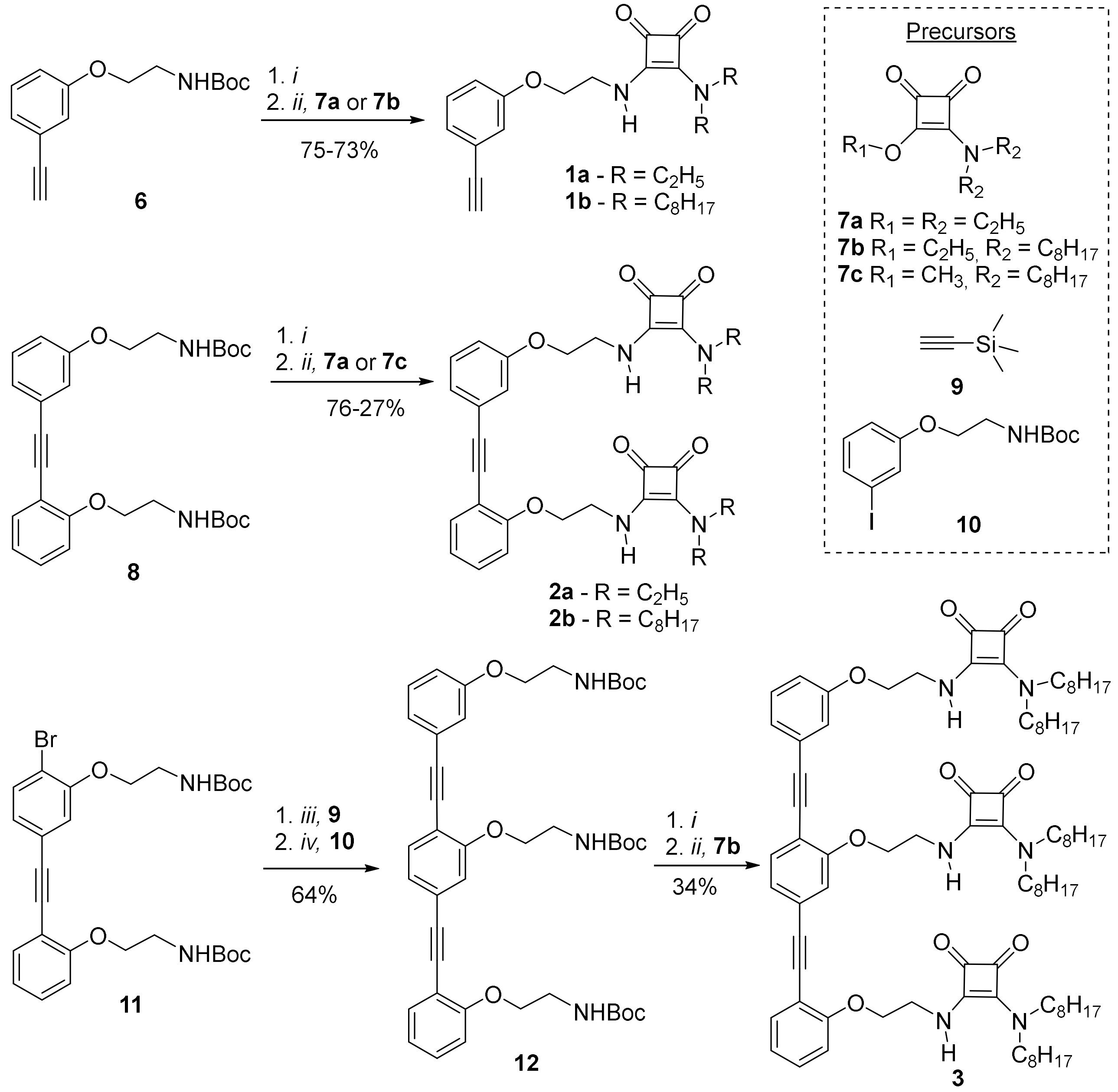

i. DCM-TFA 9-1, r.t., 1-2 h; *ii.* DIPEA or TEA, MeOH or EtOH, r.t., 16-72 h; *iii.* $Pd(PPh_3)_2Cl_2$, CuI, piperidine, 60°C, 2 h; *iv.* $Pd(PPh_3)_2Cl_2$, CuI, DIPA, H_2SiF_6, H_2O, r.t., 72 h.

3-(Diethylamino)-4-((2-(3-ethynylphenoxy)ethyl)amino)cyclobut-3-ene-1,2-dione (**1a**)

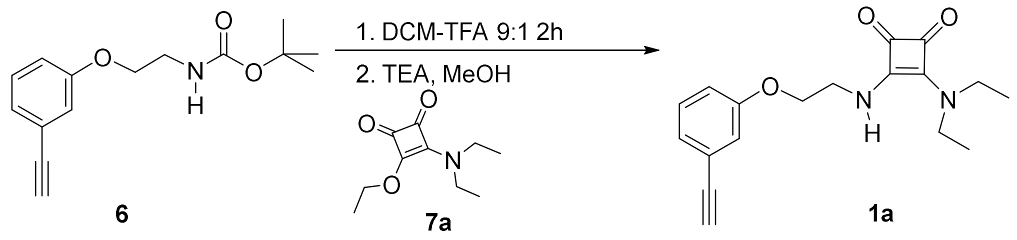

Compound **6** (200 mg, 0.77 mmol) was dissolved in a 9:1 mixture of DCM and TFA (5 mL) and the solution was stirred at room temperature for 2 h. The solvents were removed under reduced pressure. The crude was redissolved in EtOH (1 mL), DIPEA (380 µL, 2.22 mmol) and a solution of **7a** (151 mg, 0.77 mmol) in EtOH (2 mL) were added and the mixture was stirred for 72 h. A solid appeared in suspension, which was separated by filtration. The solution was then stirred for one week. More solid appeared, which was again separated by filtration. The solid fractions were combined and washed with hexane to afford the product as a white solid (180 mg, 75%). **^{1}H NMR** (400 MHz, $(CD_3)_2SO$): δ = 7.72 (t, *J* = 5.3 Hz, 1H), 7.28 (t, *J* = 7.8 Hz, 1H), 7.02 (mult., 3H), 4.17 (s, 1H), 4.14 (t, *J* = 5.3 Hz, 2H), 3.91 (q, *J* = 5.3 Hz, 2H), 3.52 (br, 4H), 1.12 (t, *J* = 7.0 Hz, 1H). **^{13}C NMR** (101 MHz, $(CD_3)_2SO$): δ = 183.2, 182.3, 167.5, 167.4, 158.8, 130.4, 124.8, 123.3, 117.7, 116.5, 83.8, 81.1, 68.1, 43.9, 43.1, 15.5. **HRMS** (ESI+): calcd for $C_{18}H_{20}O_3N_2Na$ $[M+Na]^+$: 335.1366, found: 335.1366.

3-(Dioctylamino)-4-((2-(3-ethynylphenoxy)ethyl)amino)cyclobut-3-ene-1,2-dione (**1b**)

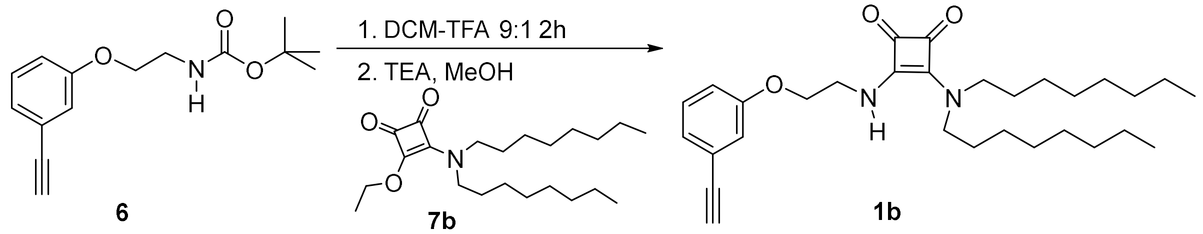

Compound **5** (100 mg, 0.38 mmol) was dissolved in a 9:1 mixture of DCM and TFA (5 mL) and the solution was stirred at room temperature for 2 h. The solvents were removed under reduced pressure. The crude was redissolved in MeOH (0.5 mL), TEA (270 µL, 1.92 mmol) and a solution of **7b** (140 mg, 0.38 mmol) in MeOH (0.5 mL) were added and the mixture was stirred for 72 h. The solvent was removed under reduced pressure and the crude was purified by column chromatography (SiO_2, DCM:AcOEt (85:15)) to afford the product as a white solid (135 mg, 73%). ***R_f***: 0.3 (DCM-AcOEt(15%)). **^{1}H NMR** (400 MHz, CD_2Cl_2): δ = 7.28 (t, *J* = 8.0 Hz, 1H), 7.14 (d, *J* = 7.6 Hz, 1H), 7.06 (s, 1H), 6.95 (d, *J* = 8.3 Hz, 1H), 5.68 (br, 1H), 4.17 (mult., 4H), 3.47 (br, 4H), 3.16 (s, 1H), 1.63 (mult., 4H), 1.28 (mult., 20H), 0.91 (mult., 6H). **^{13}C NMR** (101 MHz, CD_2Cl_2): δ = 183.7, 182.8, 168.2, 166.4, 158.2, 129.6, 125.1, 123.2, 117.8, 115.5, 83.1, 77.2, 67.9, 49.8, 43.6, 31.8, 29.6, 29.3, 29.2, 26.5, 22.6, 13.8. **HRMS** (ESI+): calcd for $C_{30}H_{44}O_3N_2Na$ $[M+Na]^+$: 503.3244, found: 503.3242.

3-(Diethylamino)-4-((2-(2-((3-(2-((2-(diethylamino)-3,4-dioxocyclobut-1-en-1-yl)amino)ethoxy)phenyl)-ethynyl)phenoxy)ethyl)amino)cyclobut-3-ene-1,2-dione (2a)

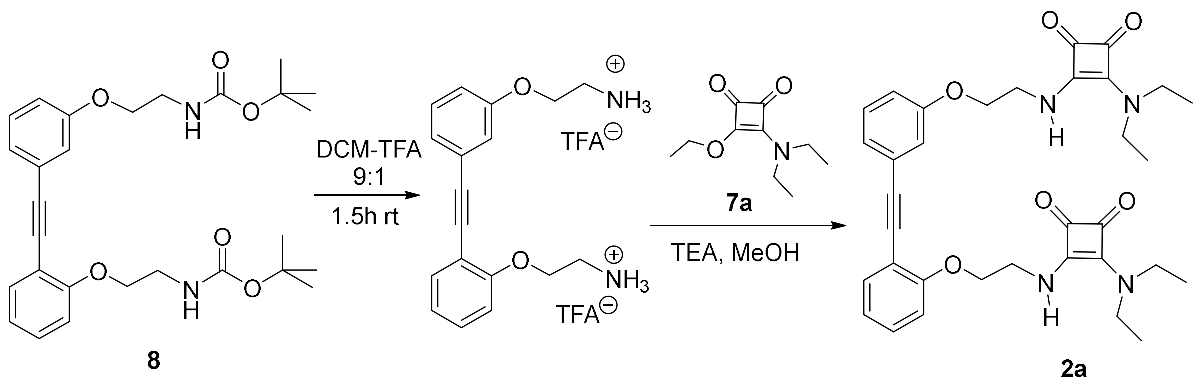

Compound **8** (50 mg, 0.10 mmol) was dissolved in a 9:1 mixture of DCM and TFA (5 mL) and the solution was stirred at room temperature for 1 h. The solvents were removed under reduced pressure. The crude was redissolved in EtOH (0.5 mL), DIPEA (53 µL, 0.22 mmol) and a solution of **7a** (43 mg, 0.22 mmol) in EtOH (0.5 mL) were added and the mixture was stirred for 16 h. The solvent was removed under reduced pressure and the crude washed with MeOH to afford the product as a white solid (16 mg, 27%). **^{1}H NMR** (400 MHz, $(CD_3)_2SO$): δ = 7.75 (t, *J* = 5.8 Hz, 1H), 7.67 (t, *J* = 5.9 Hz, 1H), 7.48 (d, *J* = 7.5 Hz, 1H), 7.38 (t, *J* = 7.9 Hz, 1H), 7.28 (t, *J* = 8.1 Hz, 1H), 7.15 (d, *J* = 8.4 Hz, 1H), 7.06 (mult., 2H), 6.99 (mult., 2H), 4.25 (t, *J* = 5.5 Hz, 2H), 4.18 (t, *J* = 5.5 Hz, 2H), 4.04 (q, *J* = 5.5 Hz, 2H), 3.95 (q, *J* = 5.5 Hz, 2H), 3.49 (br, 8H), 1.13 (t, *J* = 7.1 Hz, 3H), 1.08 (t, *J* = 7.1 Hz, 3H). **HRMS** (ESI+): calcd for $C_{34}H_{38}O_6N_4Na$ $[M+Na]^+$: 621.2684, found: 621.2684.

3-(Dioctylamino)-4-((2-(2-((3-(2-((2-(dioctylamino)-3,4-dioxocyclobut-1-en-1-yl)amino)ethoxy)phenyl)-ethynyl)phenoxy)ethyl)amino)cyclobut-3-ene-1,2-dione (**2b**)

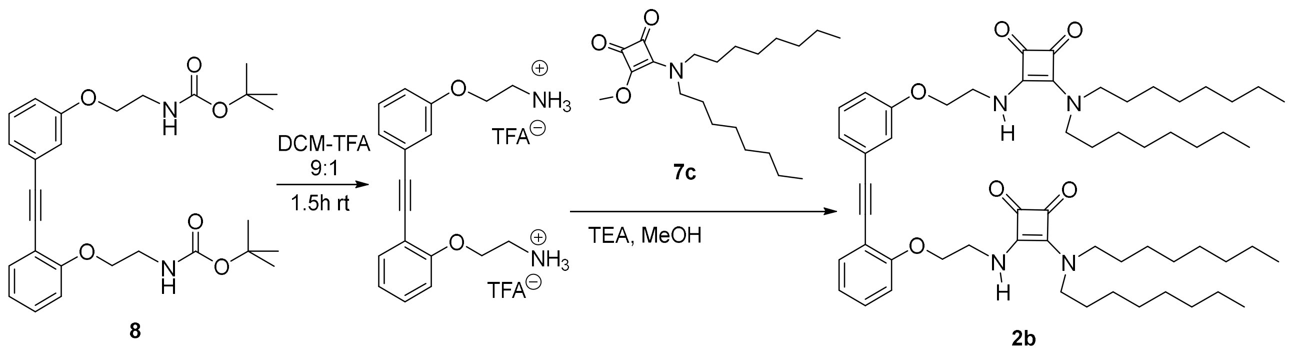

Compound **8** (50 mg, 0.10 mmol) was dissolved in a 9:1 mixture of DCM and TFA (5 mL) and the solution was stirred at room temperature for 1.5 h. The solvents were removed under reduced pressure. The crude was redissolved in MeOH (0.5 mL), TEA (56 µL, 0.40 mmol) and a solution of **7c** (85 mg, 0.24 mmol) in MeOH (0.5 mL) were added and the mixture was stirred for 16 h. The solvent was removed under reduced pressure and the crude was purified by column chromatography (SiO_2, DCM:AcOEt (from 15% to 50% AcOEt)) to afford the product as a white solid (72 mg, 76%). ***R_f***: 0.4 (DCM:AcOEt (25% AcOEt)). **^{1}H NMR** (500 MHz, CD_2Cl_2): δ = 7.41 (d, *J* = 7.6 Hz, 1H), 7.26 (t, *J* = 7.9 Hz, 1H), 7.18 (t, *J* = 7.9 Hz, 1H), 7.00 (d, *J* = 7.8 Hz, 1H), 6.93 (mult., 2H), 6.89 (d, *J* = 8.4 Hz, 1H), 6.80 (d, *J* = 8.4 Hz, 1H), 6.12 (br, 1H), 5.42 (br, 1H), 4.20 (mult., 4H), 4.08 (mult., 4H), 3.37 (br, 8H), 1.51 (mult., 4H), 1.40 (mult., 4H), 1.16 (br, 40H), 0.78 (mult., 12H). **^{13}C NMR** (125 MHz, CD_2Cl_2): δ = 183.4, 183.3, 182.4, 182.0, 186.1, 167.8, 166.9, 166.7, 159.1, 158.5, 133.3, 129.9, 129.5, 124.5, 124.0, 121.3, 117.5, 114.8, 113.0, 93.5, 85.6, 68.4, 67.8, 49.7, 49.5, 43.9, 43.5, 31.8, 31.7, 29.4, 29.3, 29.2, 29.1, 26.4, 22.6, 13.8. **HRMS** (ESI+): calcd for $C_{58}H_{86}O_6N_4Na$ $[M+Na]^+$: 957.644, found: 957.6434.

tert-Butyl (2-(2-((3-(2-((tert-butoxycarbonyl)amino)ethoxy)-4-((3-(2-((tert-butoxycarbonyl)amino)ethoxy)-phenyl)ethynyl)phenyl)ethynyl)phenoxy)ethyl)carbamate (12)

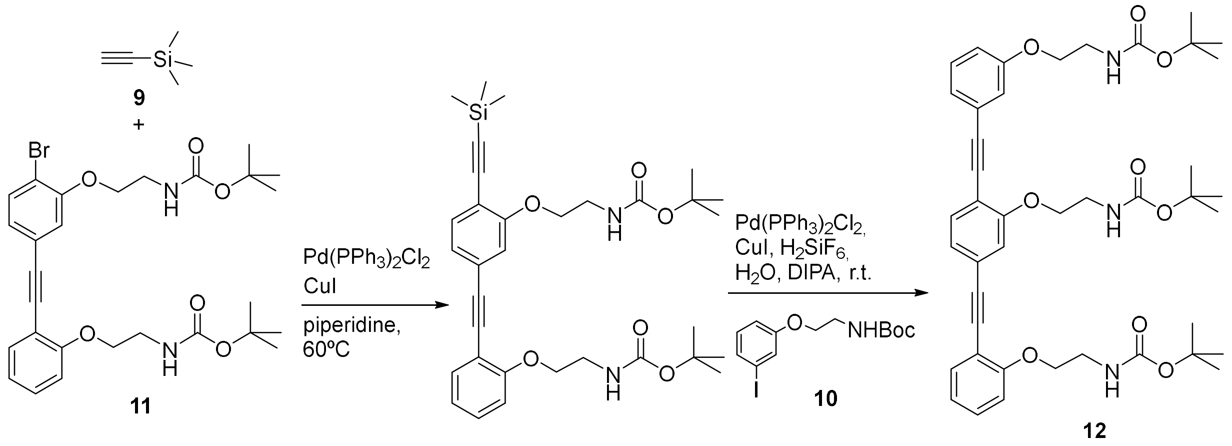

Compound **11** (200 mg, 0.35 mmol), bis(triphenylphosphine)palladium(II) dichloride (49 mg, 0.07 mmol) and copper iodide (10 mg, 0.05 mmol) were placed in a round bottom flask with an inert atmosphere. Anhydrous piperidine (2.5 mL) and trimethylsilylacetylene **9** (0.120 mL, 0.87 mmol) were added and the mixture was stirred for 2 h at 60°C. The solvent was removed under reduced pressure and the resulting crude was dissolved in DIPA (2.5 mL). Then, compound **10** (151 mg, 0.42 mmol), copper iodide (3 mg, 0.02 mmol), an aqueous solution of fluorosilicic acid (0.0015 mL, 0.003 mmol, 34% w/w) and water (0.063 mL, 3.48 mmol) were added and the resulting mixture was stirred for 72 h at room temperature. DCM (5 mL) was added to the mixture and the resulting solution was filtered. The solvents were removed from the filtrate under reduced pressure and the crude was purified by column chromatography (SiO_2, DCM:AcOEt (7.5% AcOEt)) to afford the product as a brown solid (167 mg, 64%). ***R**$_f$*: 0.56 (DCM:AcOEt (10% AcOEt)). **^{1}H NMR** (400 MHz, $(CD_3)_2CO$): δ = 7.50 (d, *J* = 7.6 Hz, 1H), 7.49 (d, *J* = 7.9 Hz, 1H), 7.38 (t, *J* = 7.9 Hz, 1H), 7.32 (t, *J* = 7.9 Hz, 1H), 7.25 (s, 1H), 7.19 (d, *J* = 7.9 Hz, 1H), 7.16 (d, *J* = 7.8 Hz, 1H), 7.12 (mult., 2H), 7.00 (mult., 2H), 6.13 (br, 3H), 4.26 (t, *J* = 5.7 Hz, 2H), 4.18 (t, *J* = 5.7 Hz, 2H), 4.11 (t, *J* = 5.7 Hz, 2H), 3.56 (mult., 4H), 3.49 (q, *J* = 5.7 Hz, 2H), 1.40 (br, 27H). **HRMS** (ESI+): calcd for $C_{43}H_{53}O_9N_3Na$ $[M+Na]^+$: 778.3674, found: 778.3676.

3-(Dioctylamino)-4-((2-(3-((2-(2-((2-(dioctylamino)-3,4-dioxocyclobut-1-en-1-yl)amino)ethoxy)-4-((2-(2-((2-(dioctylamino)-3,4-dioxocyclobut-1-en-1-yl)amino)ethoxy)phenyl)ethynyl)phenyl)ethynyl)phenoxy)-ethyl)amino)cyclobut-3-ene-1,2-dione (3)

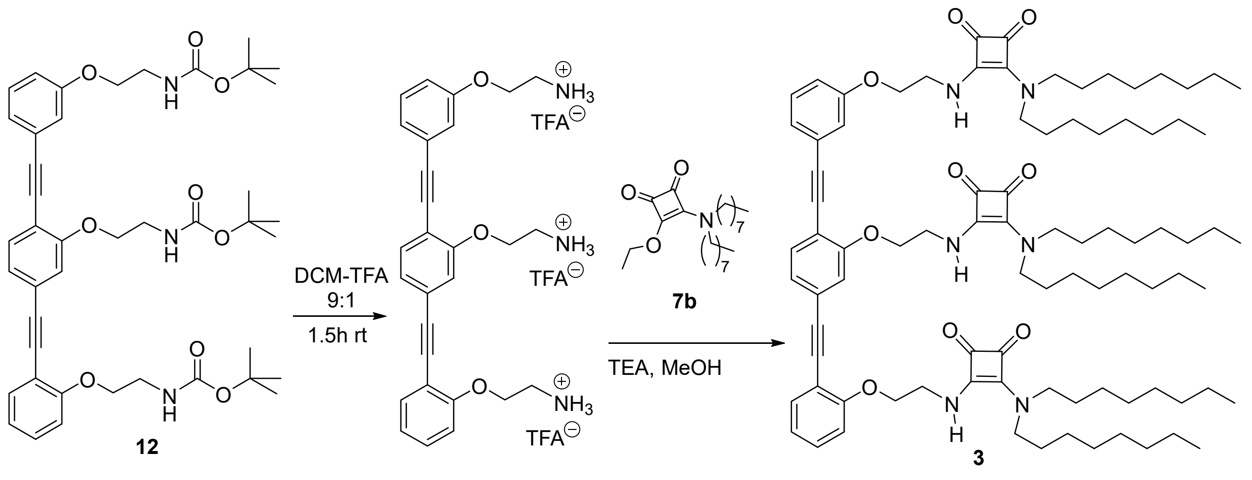

Compound **12** (70 mg, 0.09 mmol) was dissolved in a 9:1 mixture of DCM and TFA (5 mL) and the solution was stirred at room temperature for 1.5 h. The solvents were removed under reduced pressure. The crude was redissolved in MeOH (1.5 mL), DIPEA (100 µL, 0.56 mmol) and **7b** (112 mg, 0.31 mmol) were added and the mixture was stirred for 96 h. The solvent was removed under reduced pressure and the crude was purified

first by column chromatography (SiO_2, DCM-AcOEt (from 30% to 50% AcOEt)) and then by recrystallisation from acetone to afford the product as a white solid (45 mg, 34%). ***R_f***: 0.2 (DCM:AcOEt (30% AcOEt)). **^{1}H NMR** (500 MHz, CD_2Cl_2): δ = 7.50 (d, *J* = 7.6 Hz, 1H), 7.41 (d, *J* = 7.8 Hz, 1H), 7.34 (t, *J* = 8.0 Hz, 1H), 7.23 (t, *J* = 7.9 Hz, 1H), 7.15 (s, 1H), 7.08 (mult., 3H), 7.01 (t, *J* = 7.5 Hz, 1H), 6.99 (d, *J* = 8.8 Hz, 1H), 6.87 (mult., 2H), 6.76 (t, *J* = 5.6 Hz, 1H), 5.69 (t, *J* = 5.6 Hz, 1H), 4.39 (t, *J* = 5.3 Hz, 2H), 4.29 (mult., 6H), 4.22 (br, 2H), 4.16 (t, *J* = 5.5 Hz, 1H), 3.42 (br, 12H), 1.52 (br), 1.23 (br, 60H), 0.85 (mult., 18H). **^{13}C NMR** (125 MHz, CD_2Cl_2): δ = 183.5, 183.3, 183.1, 182.3, 182.3, 181.7, 168.0, 167.8, 167.6, 167.4, 167.3, 167.1, 159.3, 159.1, 158.6, 133.6, 132.4, 130.1, 129.4, 124.6, 124.4, 124.0, 123.8, 121.2, 117.5, 115.3, 115.2, 113.1, 112.7, 95.3, 93.3, 87.4, 85.6, 68.3, 67.8, 67.7, 49.6, 49.5, 49.4, 44.0, 43.8, 43.4, 31.9, 31.8, 29.4, 29.4, 29.3, 29.2, 29.2, 26.5, 26.4, 22.7, 22.6, 13.9. **HRMS** (ESI+): calcd for $C_{88}H_{128}O_9N_6Na$ $[M+Na]^+$: 1435.9635, found: 1435.9643.

3. ^1H NMR experiments in CD_2Cl_2 for compounds 1b, 2b, 3, 4 and 5

The conformational and self-assembly properties of compounds **1b**, **2b**, **3**, **4** and **5** dissolved in CD_2Cl_2 were studied by ^1H NMR spectroscopy.

The spectra registered in pure CD_2Cl_2 were referenced to the signal of $CDHCl_2$ at 5.32 ppm.

The spectra registered in presence of DMSO-*d*$_6$ were referenced with the signal of TMS at 0 ppm, because the signal of $CDHCl_2$ was significantly affected during the DMSO-*d*$_6$ titrations (see section 3.3).

3.1. Assignment of the ^1H NMR spectra in CD_2Cl_2

The ^1H NMR spectra of compounds **1b** and **15** were easily assigned based on the multiplicity and chemical shifts of the signals observed (Figure S1 and Figure S2).

The ^1H NMR spectra of compounds **2b**, **3**, **4** and **5** were assigned based on the multiplicity and chemical shifts of the signals observed, as well as the COSY and NOESY spectra (from Figure S3 to Figure S6).

- Each aromatic ring from the *oligo*-phenylene ethynylene rigid-rods showed a characteristic pattern on the aromatic region of the diagonal of the COSY spectra, which permitted the assignment of the corresponding signals.
- The signals from each CH_2CH_2NH system were connected from COSY cross-peaks.
- Each CH_2CH_2NH system was connected to an aromatic ring of the *oligo*-phenylene ethynylene rigid-rods based on NOESY cross-peaks, which permitted the unequivocal assignment of all the CH_2CH_2NH signals.
- The signals of the C_2H_5, C_8H_{17} and $C_3H_6NH(CH_3)_2$ squaramide substituents showed characteristic multiplicities and chemical shifts, and showed the expected COSY cross-peaks.
- The signals of the CH_3 squaramide substituents and the 3,5-$C_6H_3(CF_3)_2$ thiourea substituents appeared as singlets and showed characteristic chemical shifts.

The spectra were assigned using numbers for the NH signals (1-4), capital letters with a subindex for the aromatic signals from the *oligo*-phenylene ethynylene rigid-rods (A-$C_{1\text{-}4}$) and lowercase letters for the rest of the CH signals (a-n).

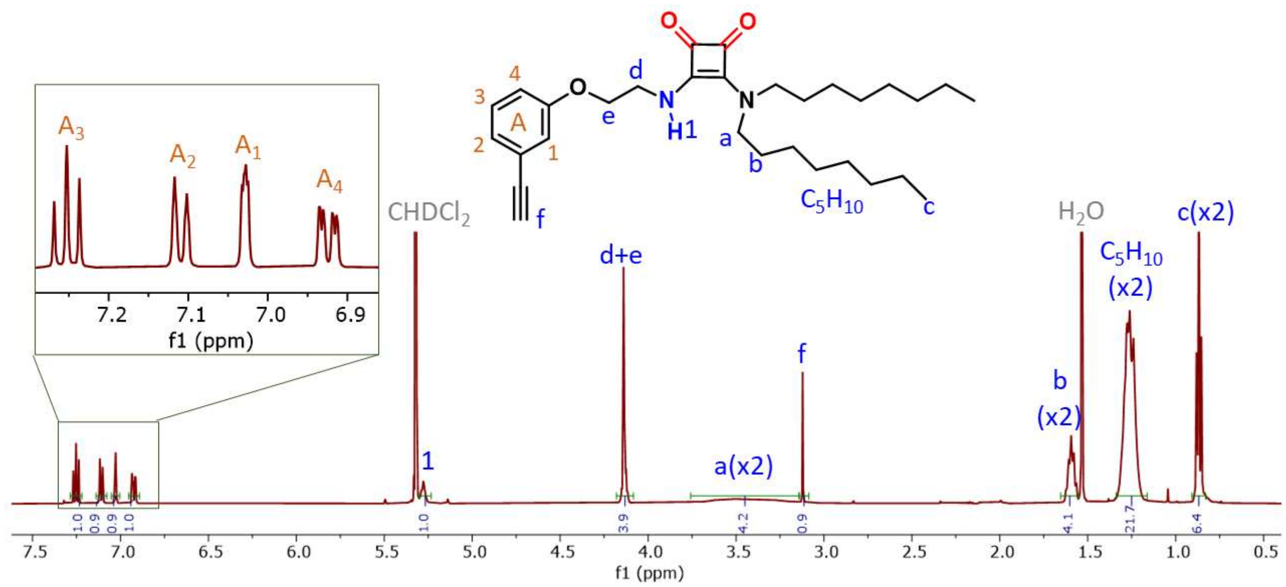

Figure S1. Assigned ^{1}H NMR spectrum (500 MHz, 298 K, CD_2Cl_2) of compound **1b** (2.5 mM).

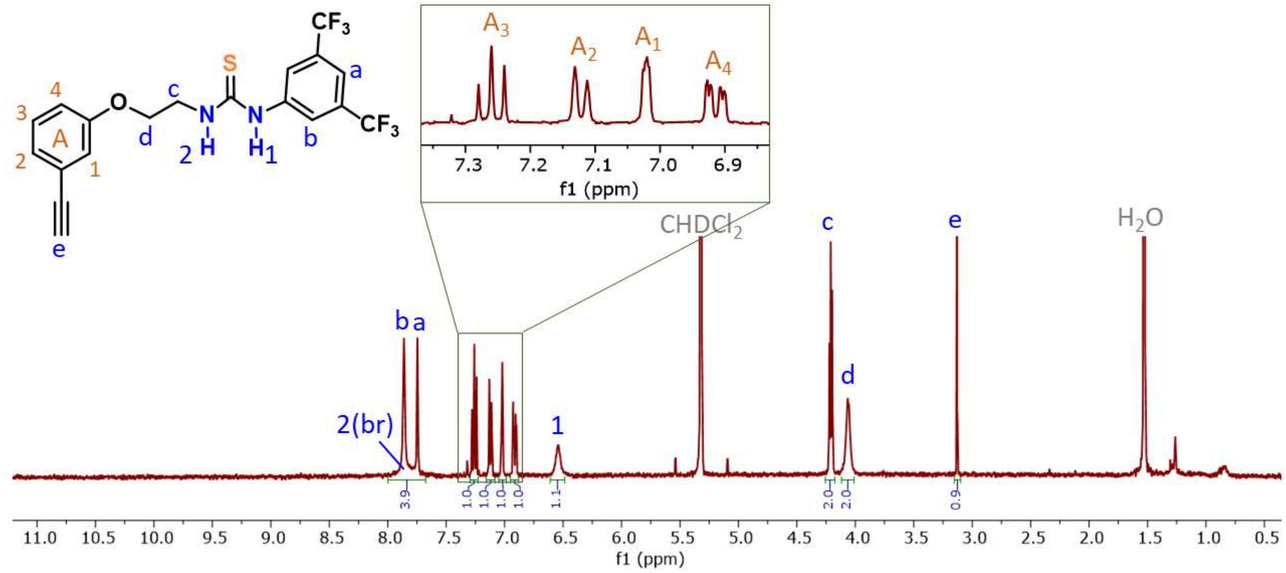

Figure S2. Assigned ^{1}H NMR spectrum (400 MHz, 298 K, CD_2Cl_2) of compound **15** (2.5 mM).

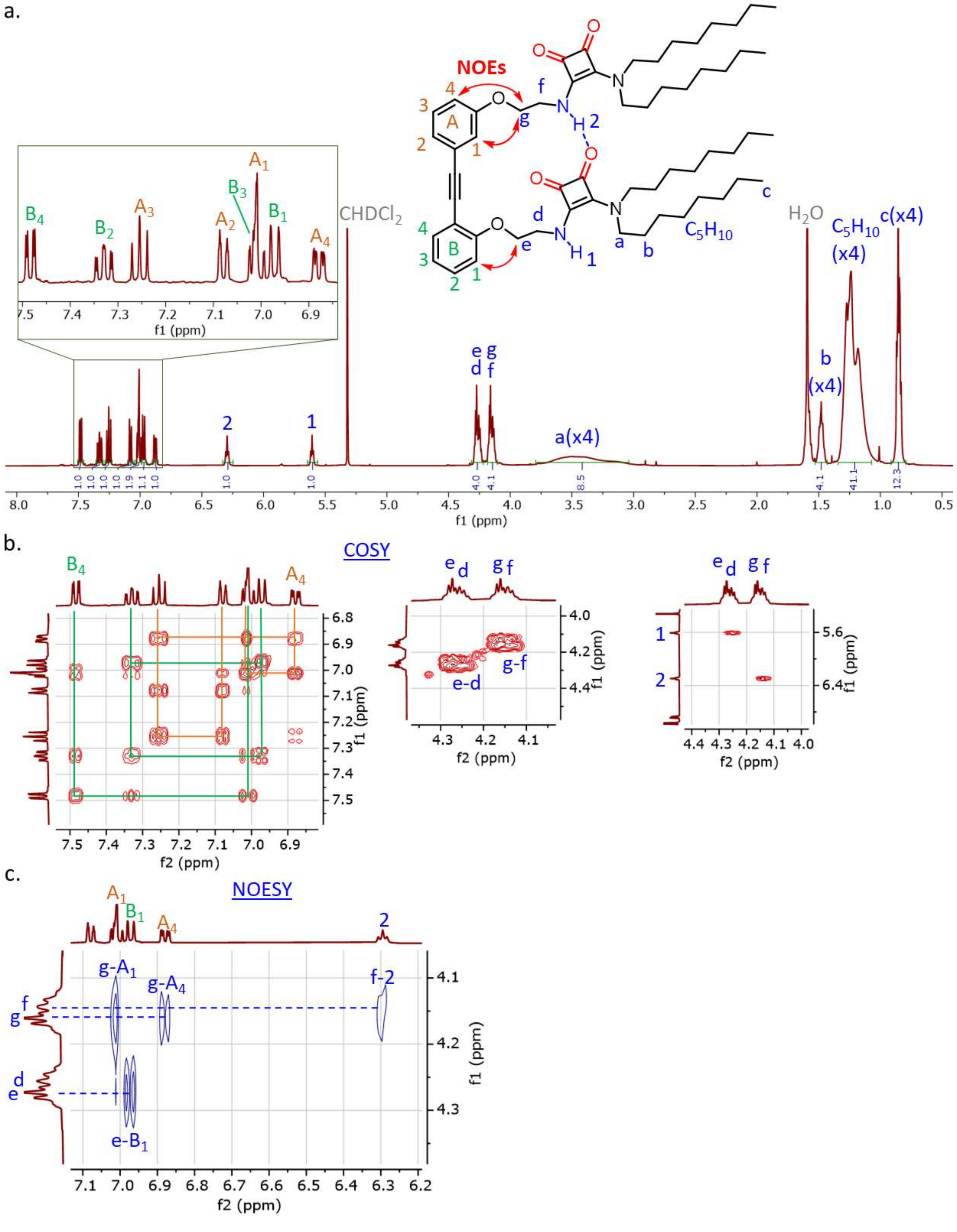

Figure S3. Assigned ^{1}H NMR spectrum (500 MHz, 298 K, CD_2Cl_2) of compound **2b** (10 mM) (a) and selected regions of the COSY (b) and NOESY (c) spectra.

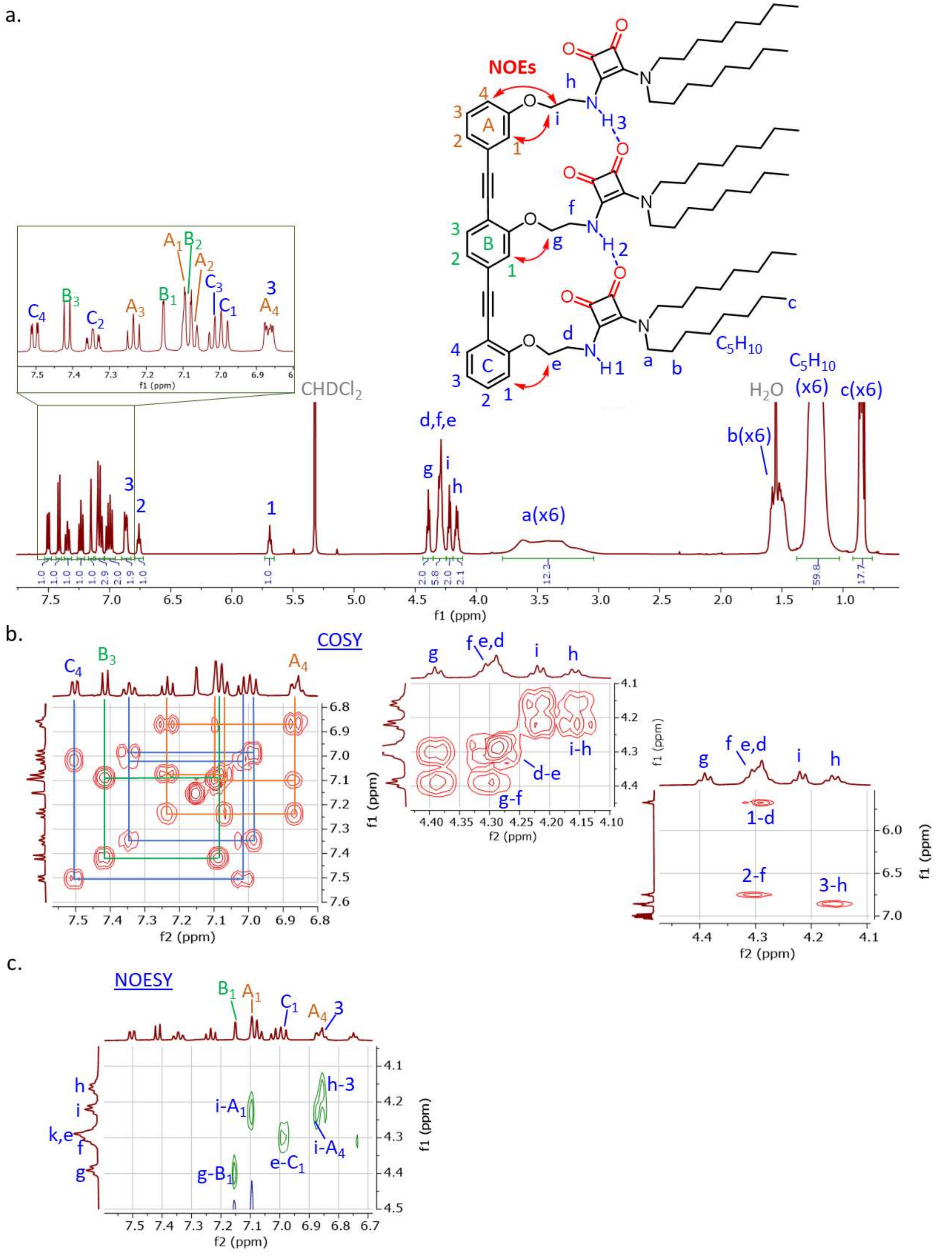

Figure S4. Assigned ^{1}H NMR spectrum (500 MHz, 298 K, CD_2Cl_2) of compound **3** (2.5 mM) (a) and selected regions of the COSY (b) and NOESY (c) spectra.

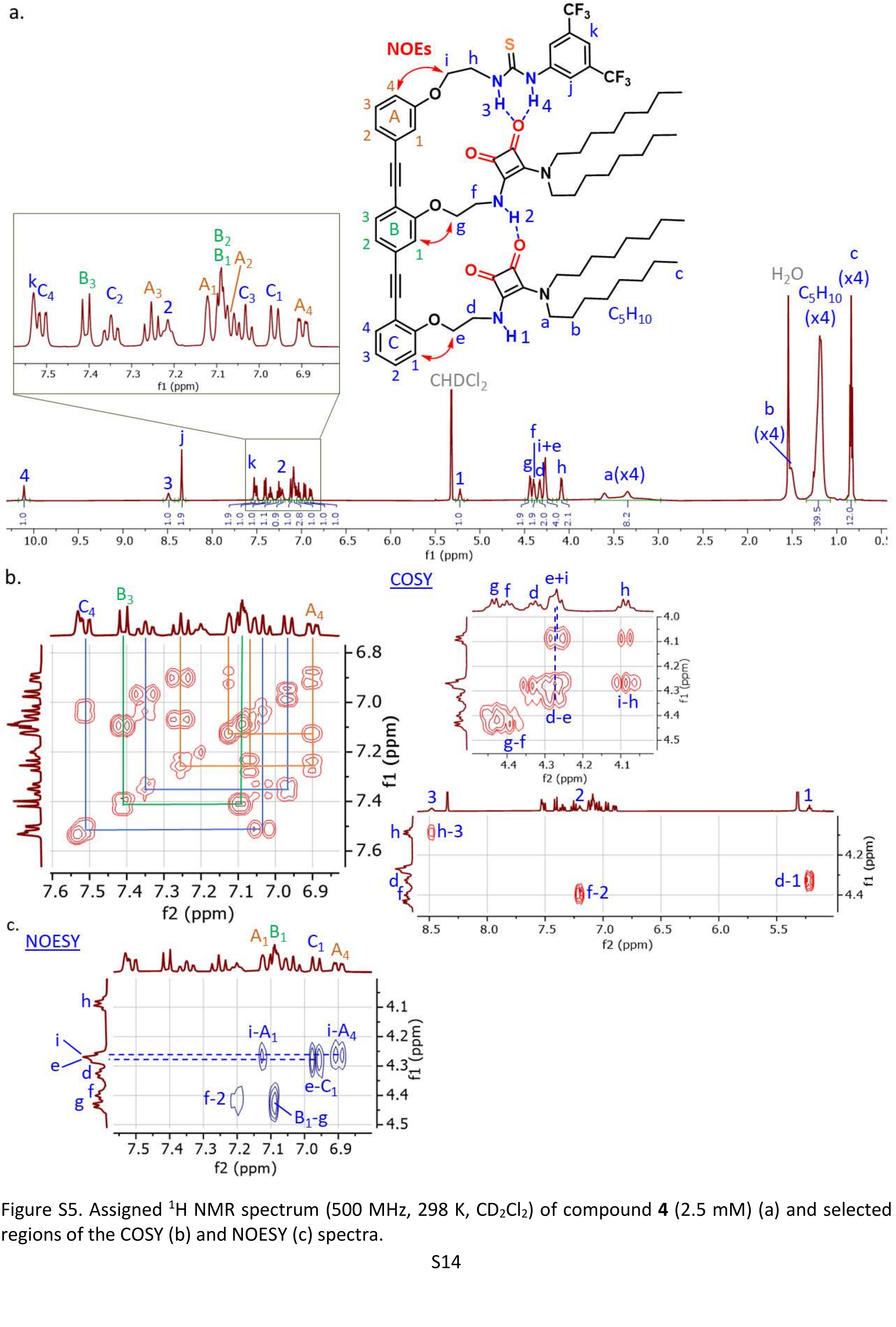

Figure S5. Assigned ^{1}H NMR spectrum (500 MHz, 298 K, CD_2Cl_2) of compound **4** (2.5 mM) (a) and selected regions of the COSY (b) and NOESY (c) spectra.

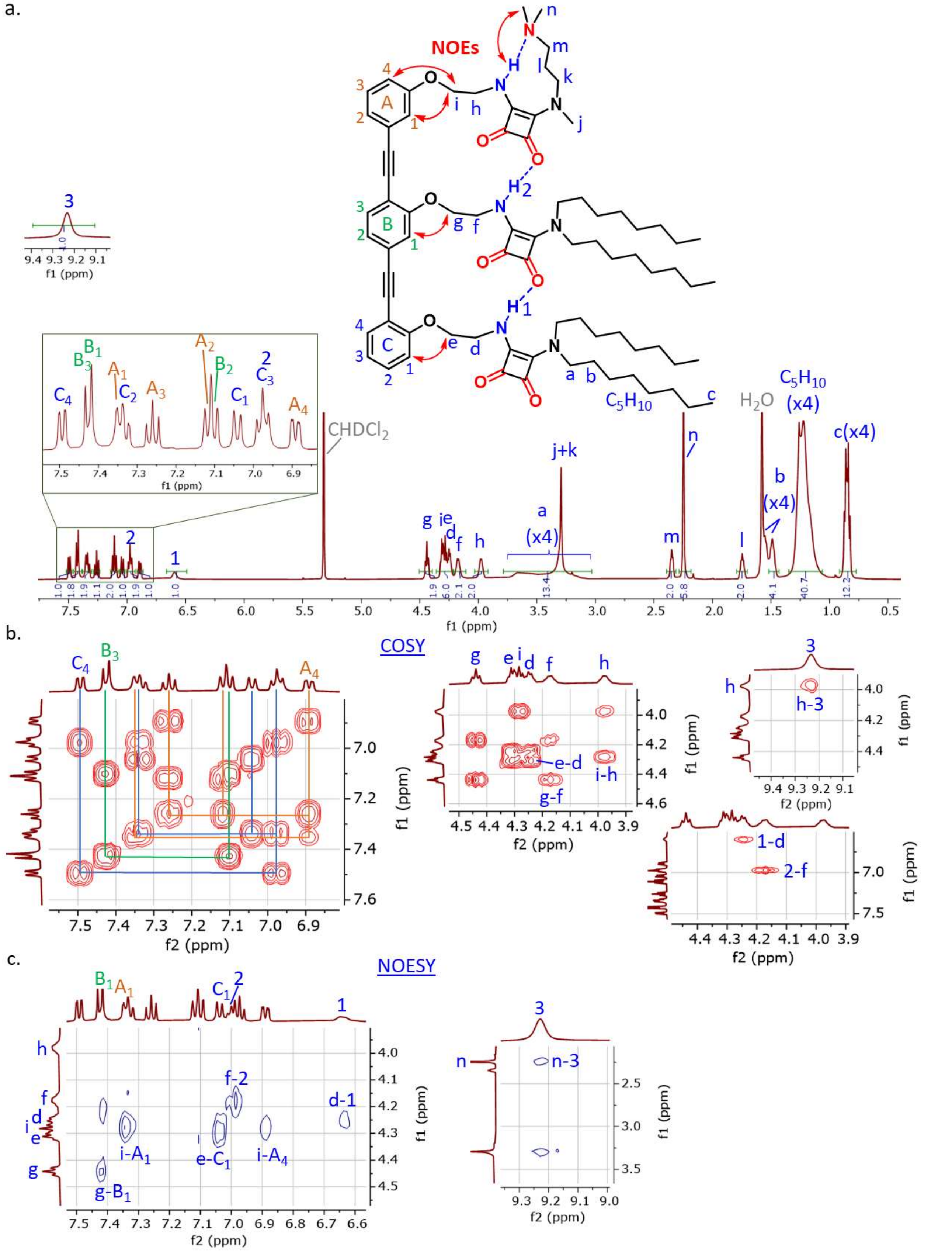

Figure S6. Assigned ^{1}H NMR spectrum (500 MHz, 298 K, CD_2Cl_2) of compound **5** (2.5 mM) (a) and selected regions of the COSY (b) and NOESY (c) spectra.

3.2. Titrations with DMSO-d_6 in CD_2Cl_2

The effect of DMSO on the different NH signals was studied by adding increasing amounts of DMSO-d_6 into solutions of the compounds in CD_2Cl_2 at 298 K. Solutions of compounds **1b**, **2b**, **3**, **4** and **5** (2.5 mM, 0.6 mL) were prepared in CD_2Cl_2 and the ^1H NMR spectra were recorded. Then aliquots of DMSO-d_6 were added to the NMR tube and ^1H NMR spectra were recorded after each addition (from Figure S7 to Figure S11).

Additions of DMSO-d_6 : 5, 5, 10, 10, 10, 20, 30, 30, 60 and 60 µL.

In certain cases, the chemical shifts of the NH signals in presence of a given amount of DMSO-d_6 were confirmed with COSY experiments.

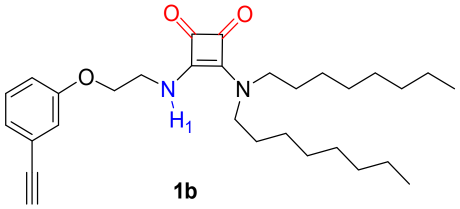

1b

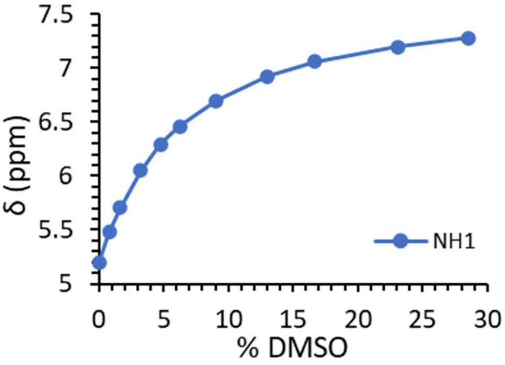

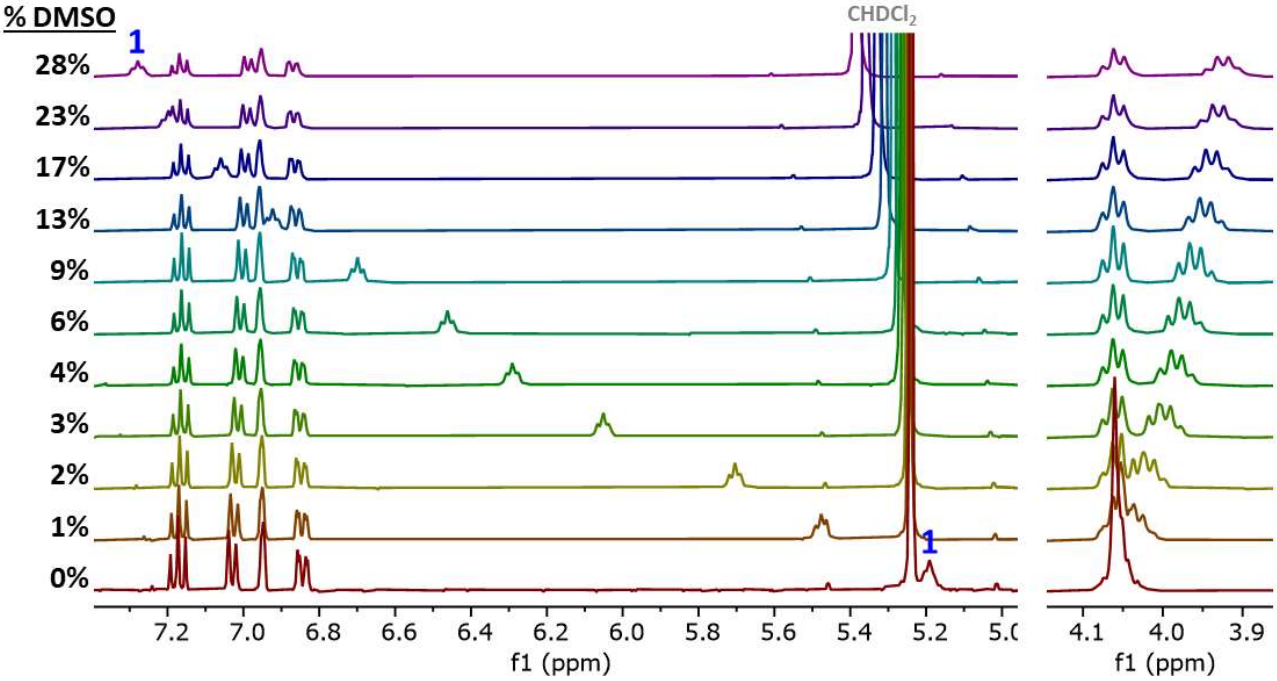

Figure S7. Selected regions of the ^1H NMR spectra (400 MHz, 298 K, CD_2Cl_2) from the titration of compound **1b** (initially 2.5 mM) with DMSO-d_6. The proportion of DMSO-d_6 is indicated as % v/v.

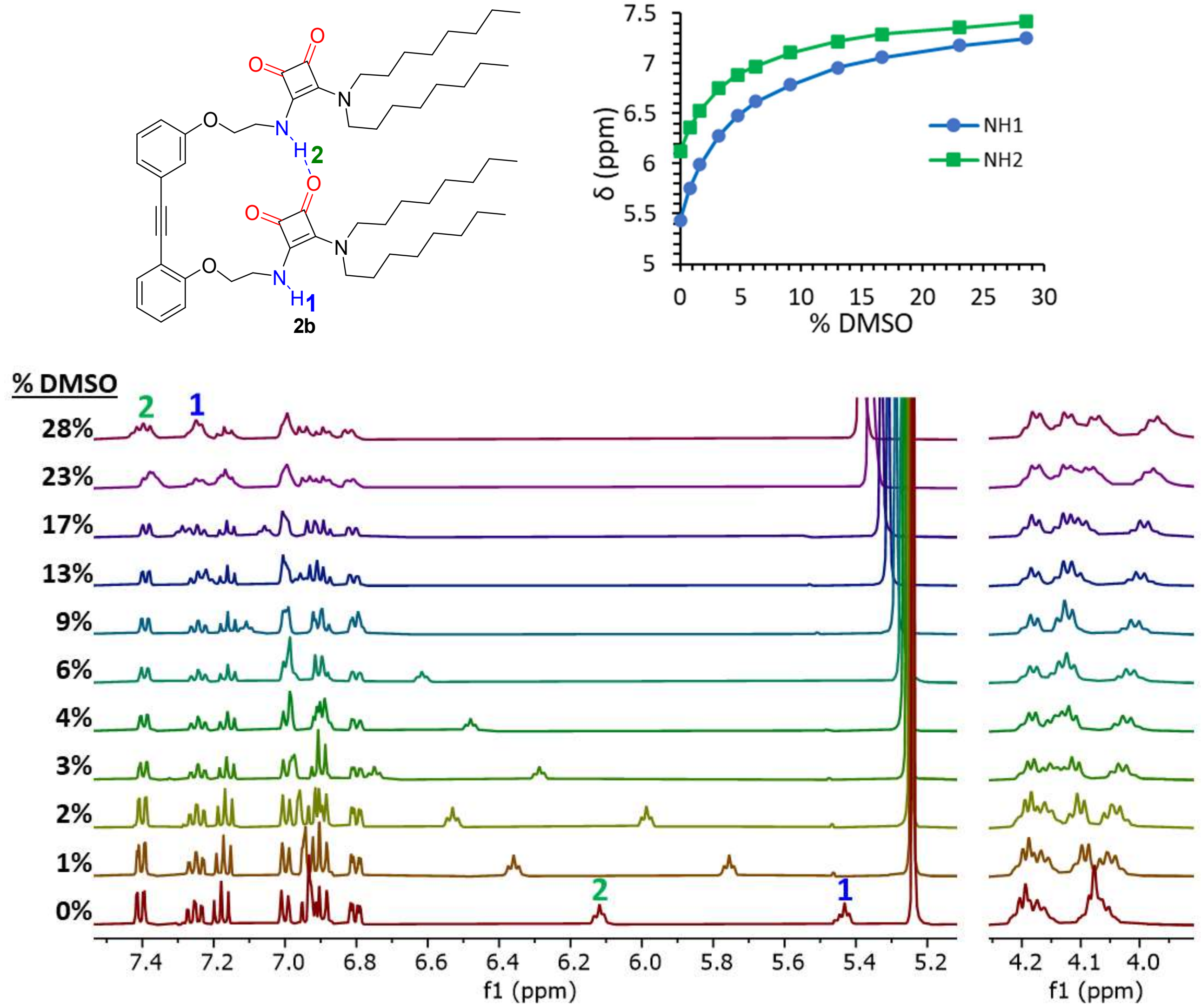

Figure S8. Selected region of the ^{1}H NMR spectra (400 MHz, 298 K, CD_2Cl_2) from the titration of compound **2b** (initially 2.5 mM) with DMSO-d_6. The proportion of DMSO-d_6 is indicated as % v/v.

a.

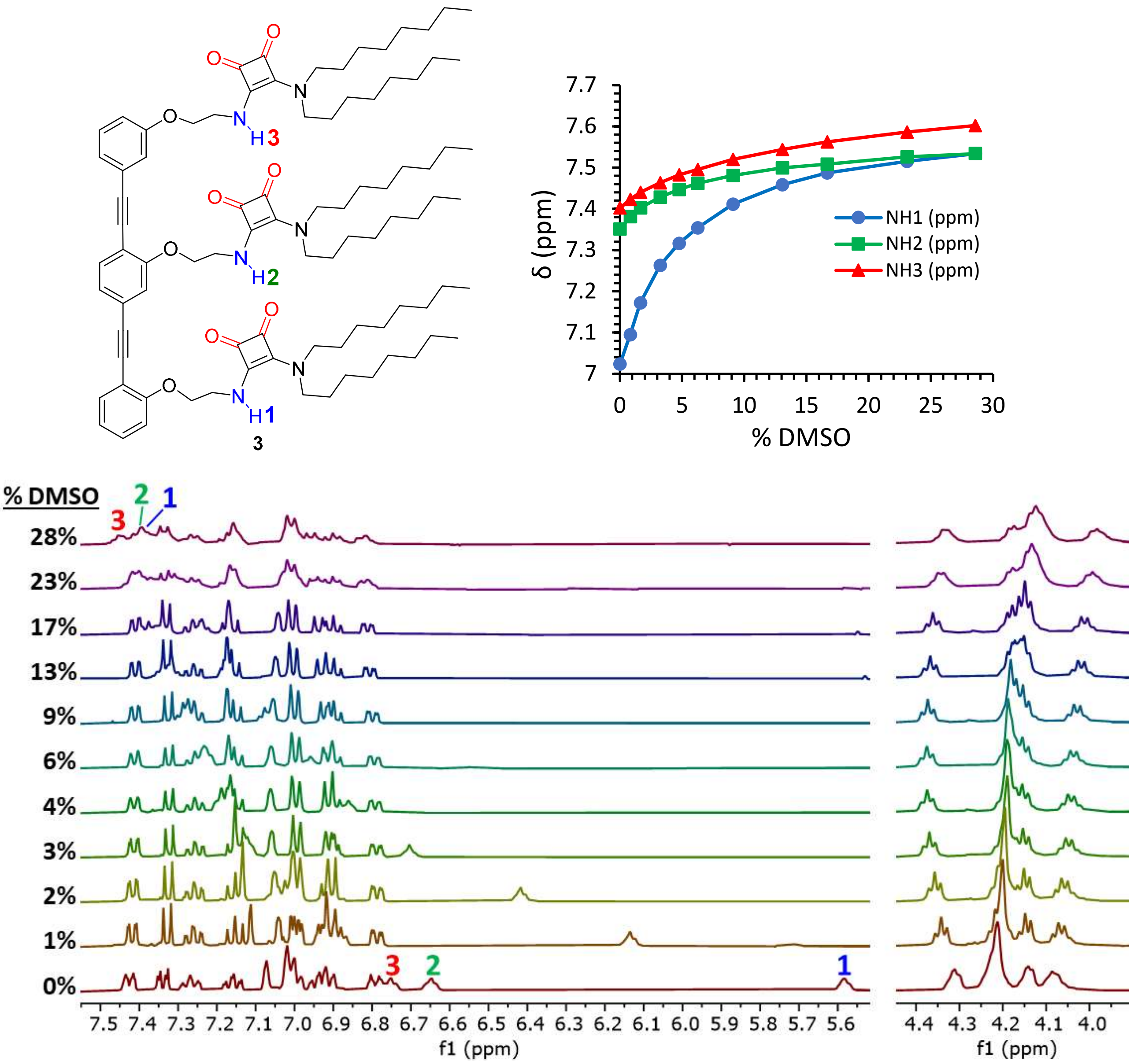

b. 28% DMSO

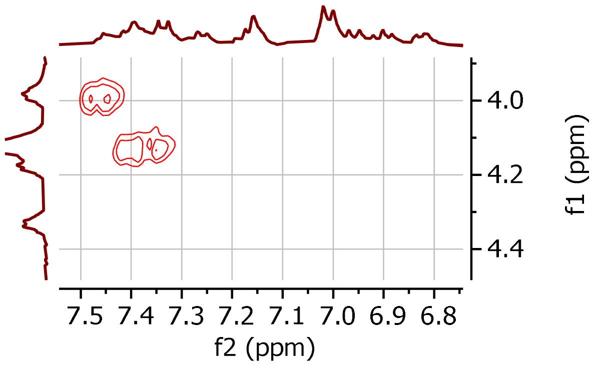

Figure S9. a. Selected region of the ^{1}H NMR spectra (400 MHz, 298 K, CD_2Cl_2) from the titration of compound **3** (initially 2.5 mM) with DMSO-d_6. The proportion of DMSO-d_6 is indicated as % v/v. b. Selected region of the COSY spectrum in presence of 28% DMSO-d_6 (the CH_2-NH cross-peaks reveal the chemical shifts of the NH signals).

a.

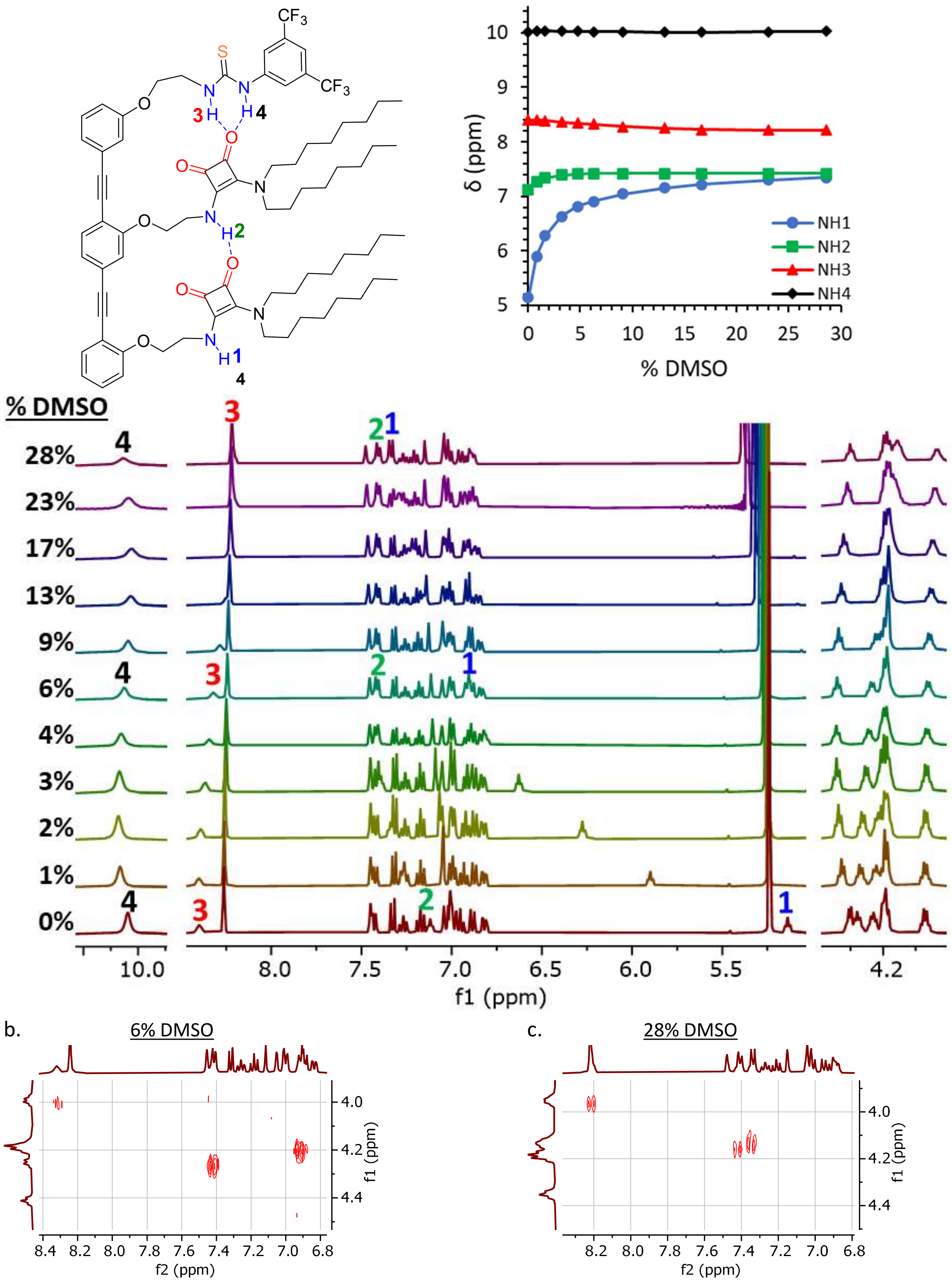

Figure S10. a. Selected region of the ^{1}H NMR spectra (400 MHz, 298 K, CD_2Cl_2) from the titration of compound **4** (initially 2.5 mM) with DMSO-d_6. The proportion of DMSO-d_6 is indicated as % v/v. b-c. Selected regions of the COSY spectrum in presence of 6% DMSO-d_6 (b) and 28% DMSO-d_6 (c) (the CH_2-NH cross-peaks reveal the chemical shifts of the NH signals).

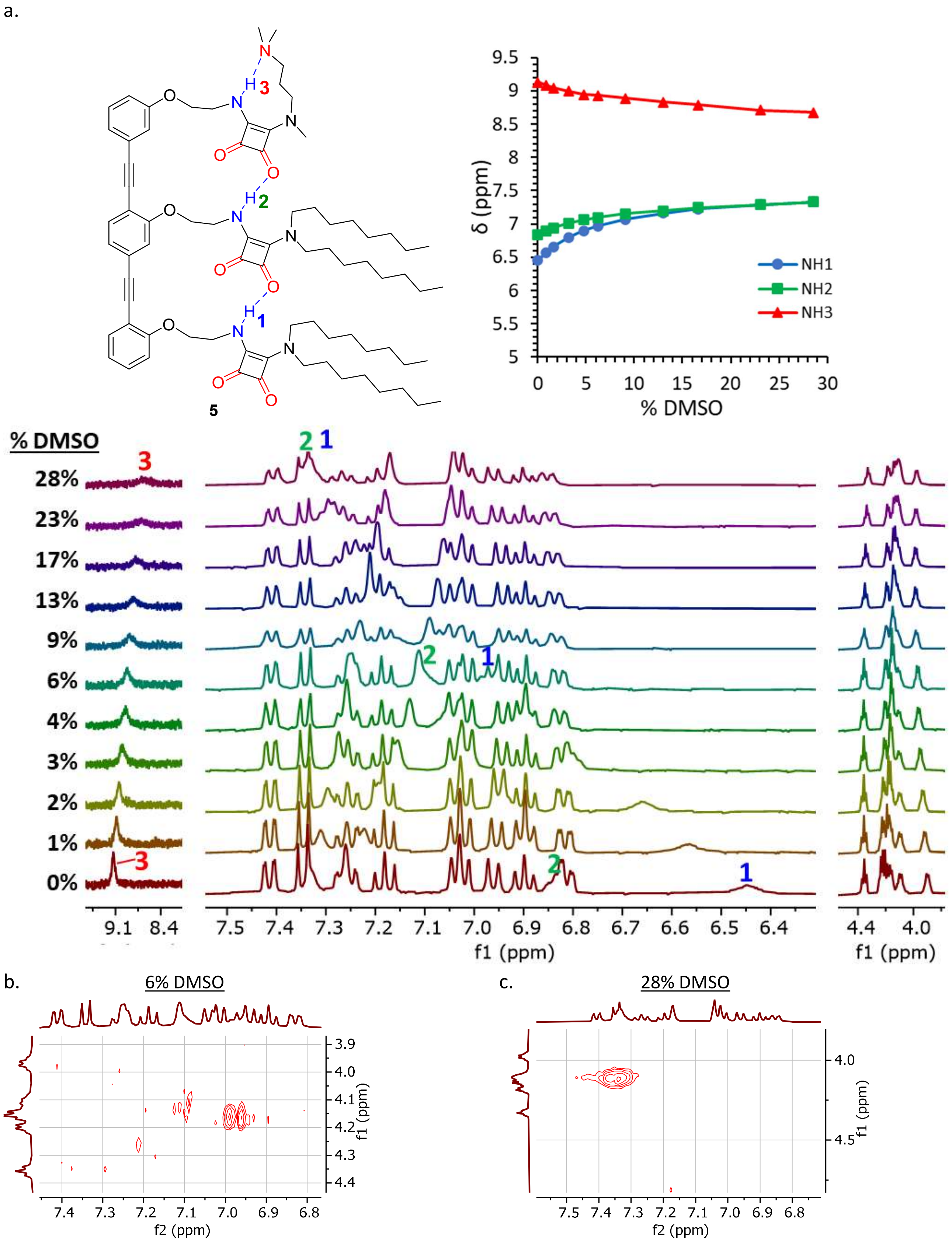

Figure S11. Selected region of the ^{1}H NMR spectra (400 MHz, 298 K, CD_2Cl_2) from the titration of compound **5** (initially 2 mM) with DMSO-d_6. The proportion of DMSO-d_6 is indicated as % v/v. b-c. Selected regions of the COSY spectrum in presence of 6% DMSO-d_6 (b) and 28% DMSO-d_6 (c) (the CH_2-NH cross-peaks reveal the chemical shifts of the NH signals).

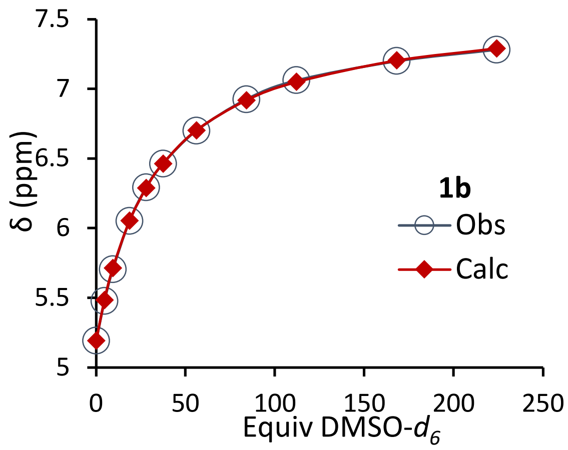

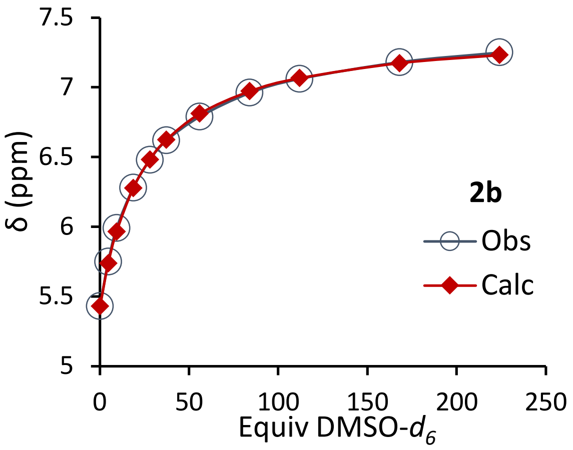

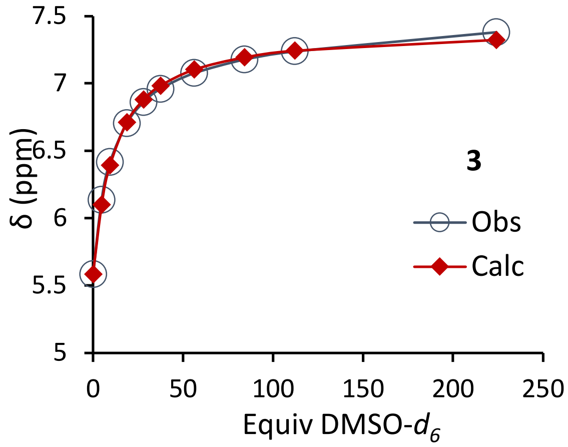

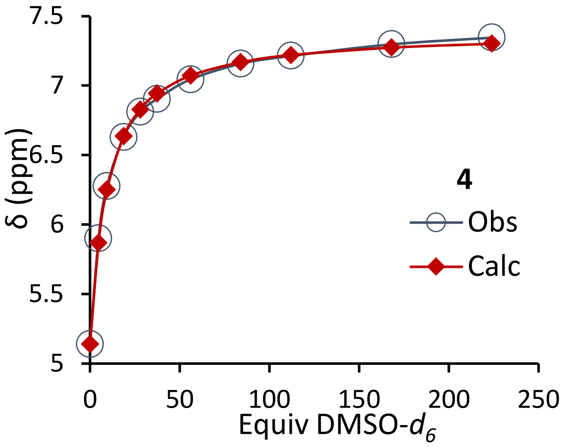

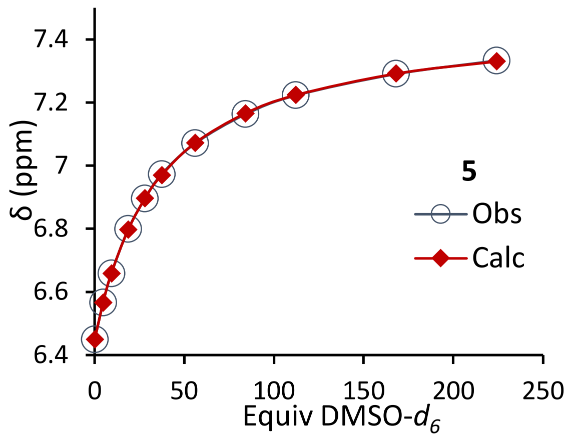

Compound	K (M^{-1})
**1b**	11.5 ± 0.1
**2b**	15.4
**3**	35.0
**4**	45.7 ± 1.6
**5**	10.6

Figure S12. Variation of the chemical shift of NH1 during the titrations of **1b**, **2b**, **3**, **4** and **5** (2.5 mM) with DMSO-d_6 at 298 K in CD_2Cl_2. Observed (obs) and calculated (calc) binding curves. The binding constants, shown in the table, were obtained from the fitting to a 1:1 binding model with http://app.supramolecular.org/bindfit/.[S3] The titrations of **1b** and **4** were performed twice to prove that the differences between the binding constants obtained are larger than the experimental uncertainty. The experimental errors are indicated as the standard deviation from two independent experiments

3.3. Dilution experiments in CD_2Cl_2

a.

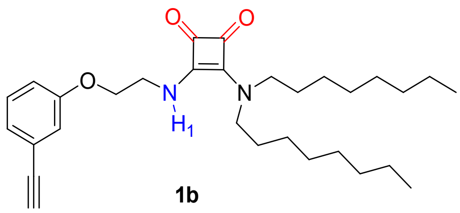

1b

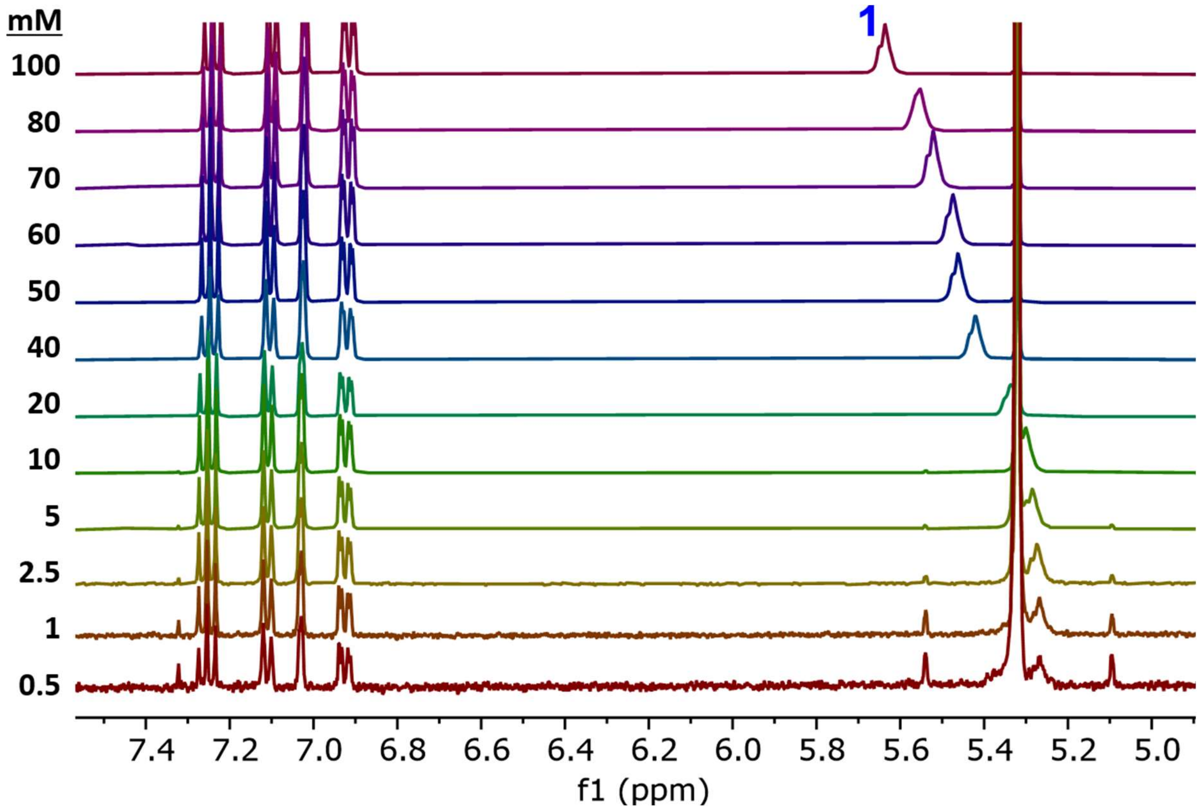

b.

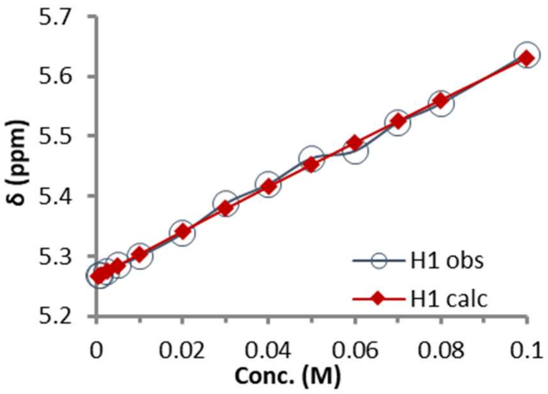

Figure S13. a. Selected region of the ^{1}H NMR spectra (400 MHz, 298 K, CD_2Cl_2) of compound **1b** at different concentrations. b. Observed (obs) and calculated (calc) curves from the fitting to a dimerization model with http://app.supramolecular.org/bindfit/. K_{dim} = 0.28 M^{-1}.

a.

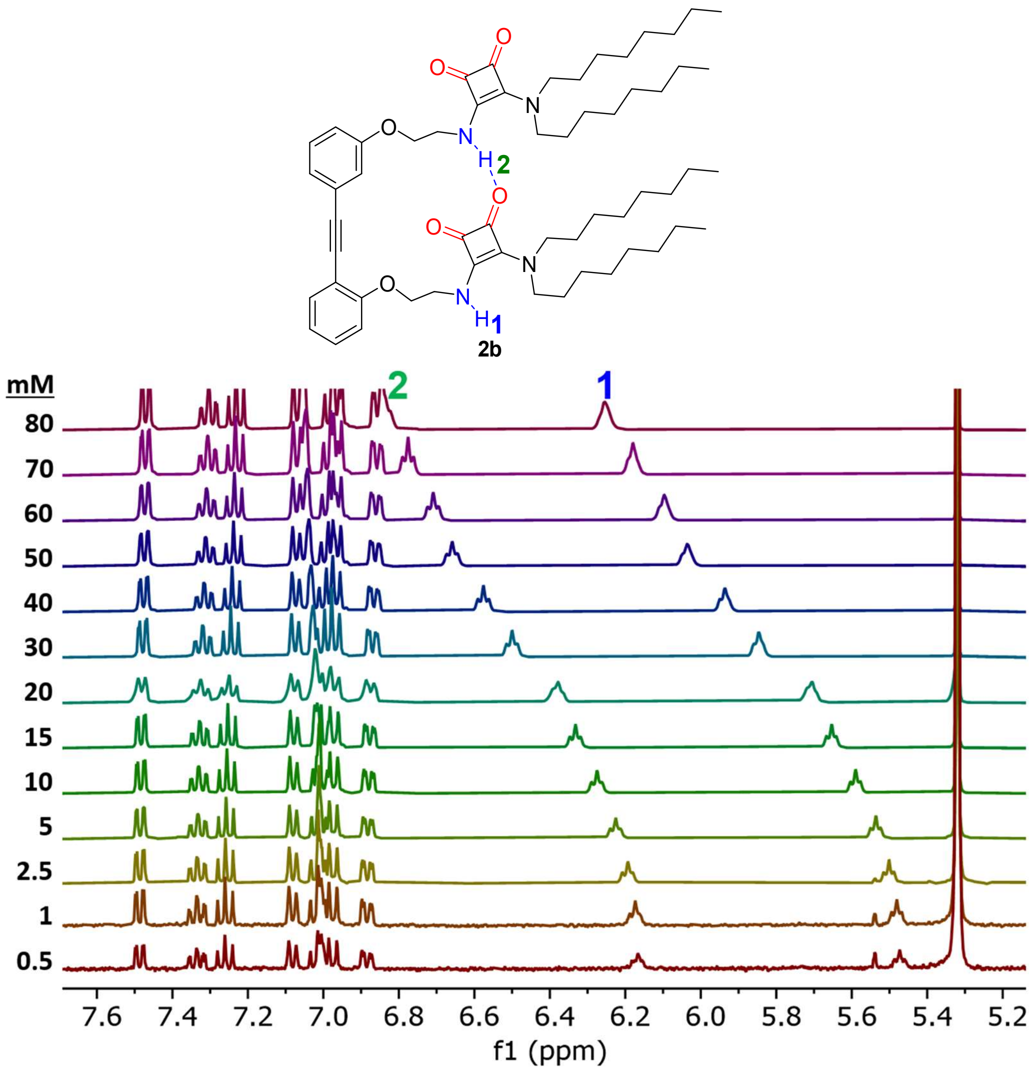

b.

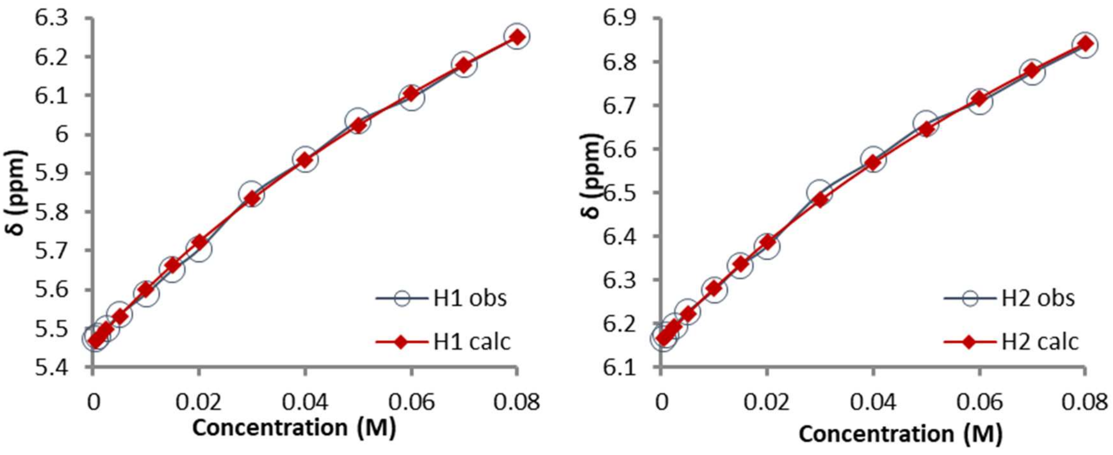

Figure S14. a. Selected region of the ^{1}H NMR spectra (400 MHz, 298 K, CD_2Cl_2) of compound **2b** at different concentrations. b. Observed (obs) and calculated (calc) curves from the fitting to a dimerization model with http://app.supramolecular.org/bindfit/. K_{dim} = 3.62 M^{-1}.

a.

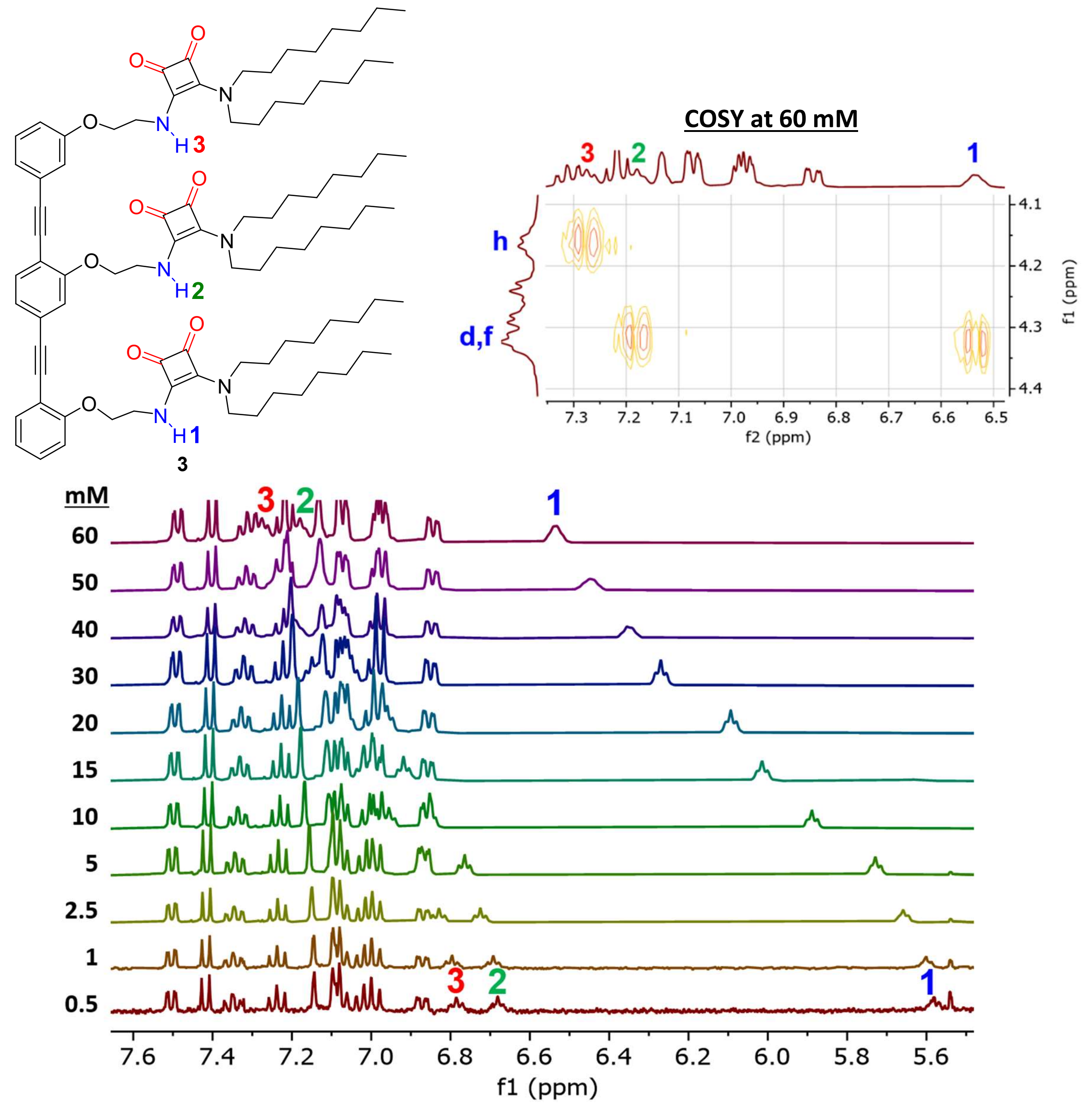

b.

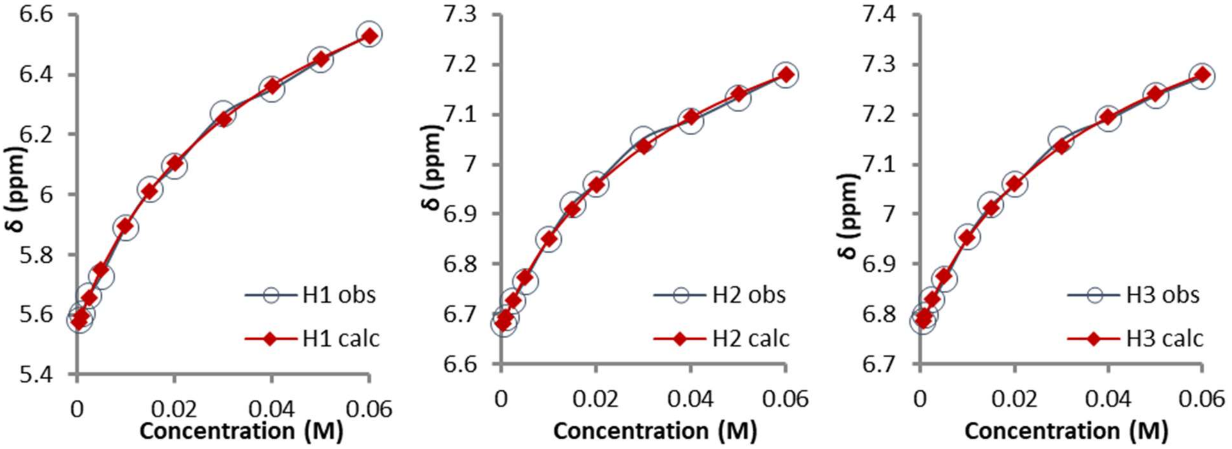

Figure S15. a. Selected region of the ^{1}H NMR spectra (400 MHz, 298 K, CD_2Cl_2) of compound **3** at different concentrations. b. Observed (obs) and calculated (calc) curves from the fitting to a dimerization model with http://app.supramolecular.org/bindfit/. K_{dim} = 19.91 M^{-1}.

a.

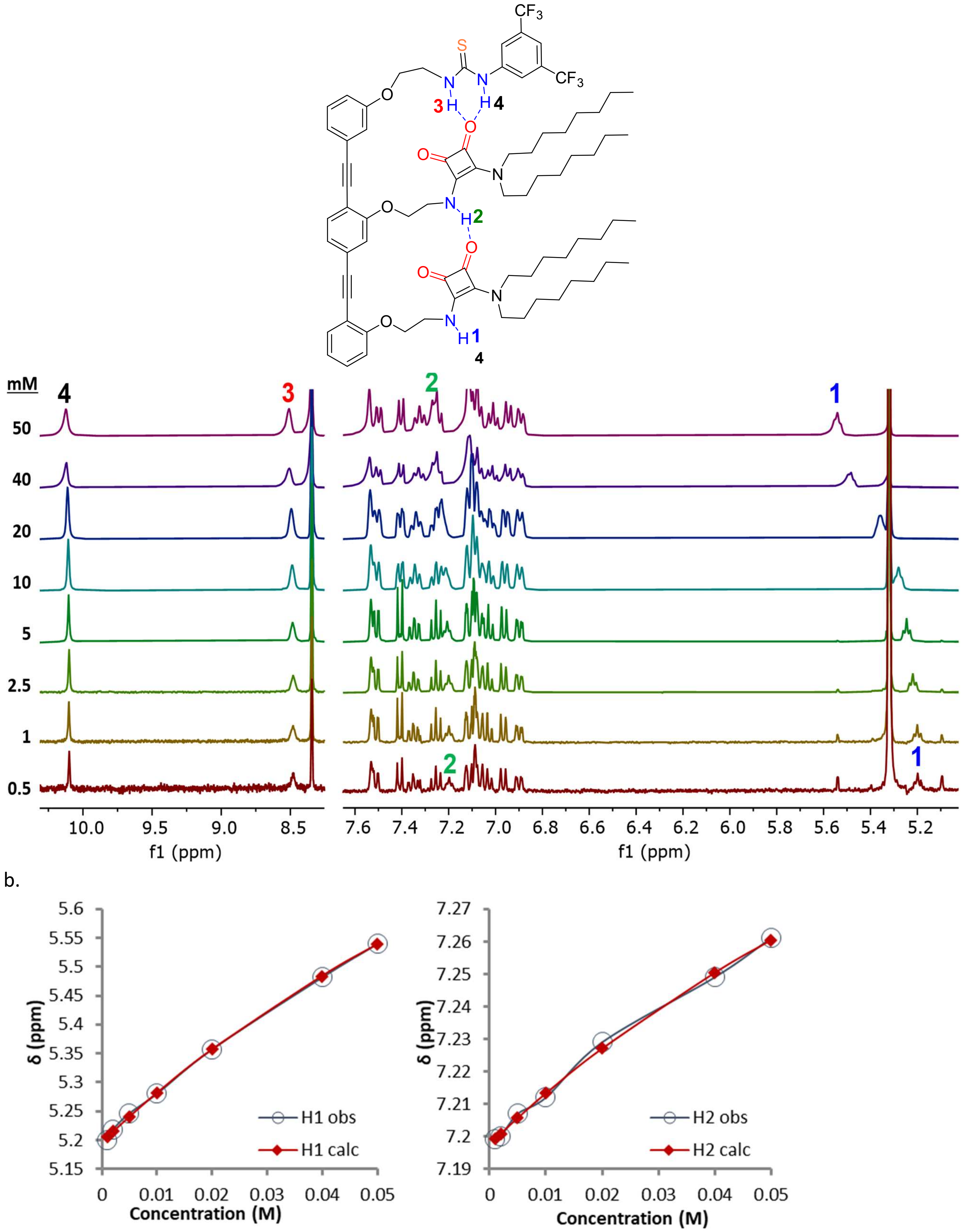

Figure S16. a. Selected region of the ^{1}H NMR spectra (400 MHz, 298 K, CD_2Cl_2) of compound **4** at different concentrations. b. Observed (obs) and calculated (calc) curves from the fitting to a dimerization model with http://app.supramolecular.org/bindfit/. K_{dim} = 3.60 M^{-1}.

a.

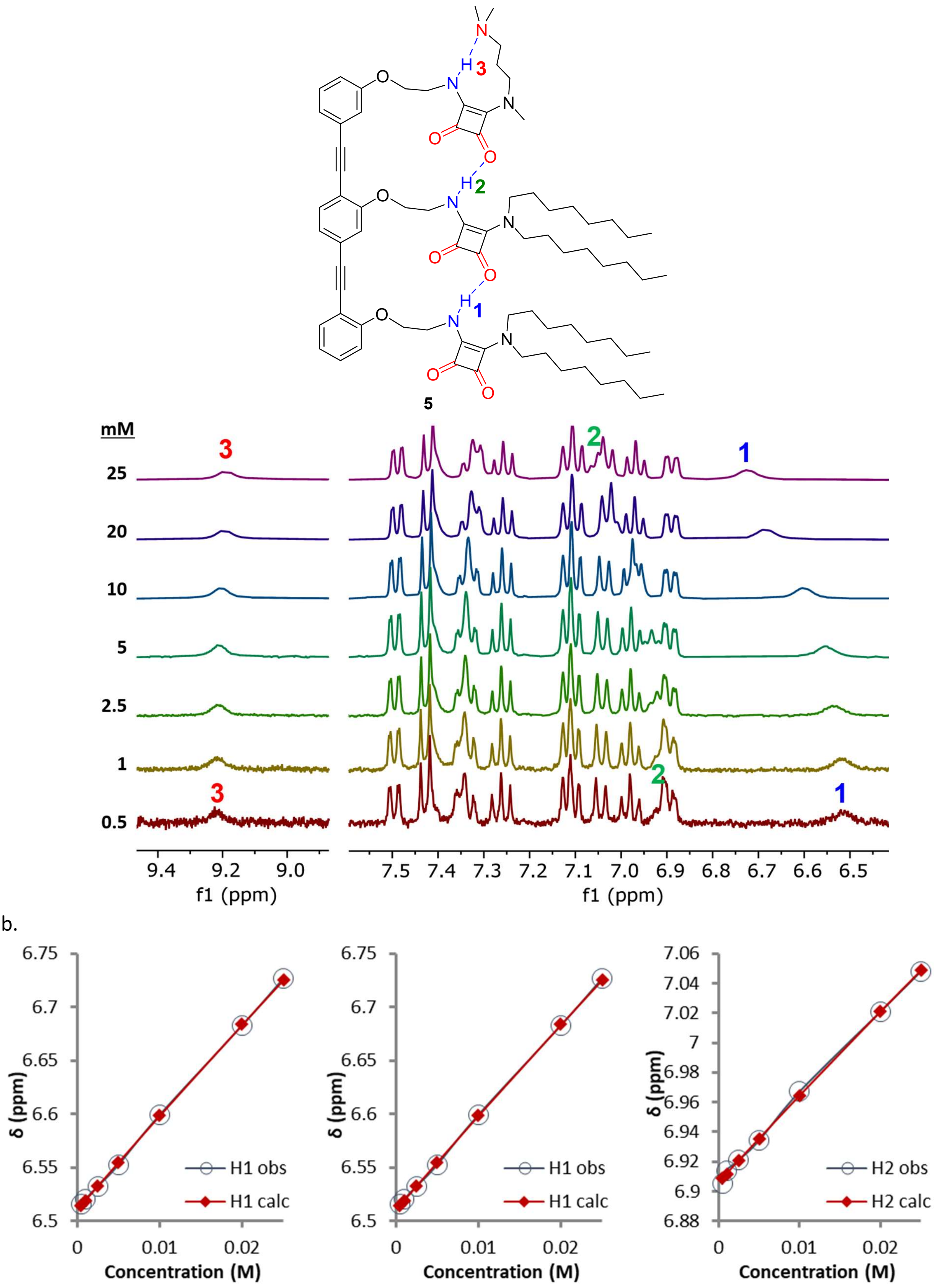

Figure S17. a. Selected region of the ^{1}H NMR spectra (400 MHz, 298 K, CD_2Cl_2) of compound **5** at different concentrations. b. Observed (obs) and calculated (calc) curves from the fitting to a dimerization model with http://app.supramolecular.org/bindfit/. K_{dim} = 0.75 M^{-1}.

3.4. VT experiments in CD_2Cl_2

For the VT experiments, 600 µL of solutions of either **1b**, **2b**, **3**, **4** or **5** (2.5 mM) were transferred into NMR tubes, and the ^{1}H NMR spectra were measured at different temperatures (from 298 down to 250 K).

All the NH signals showed a linear dependence of the temperature, and the corresponding temperature coefficients (Δδ/ΔT) were calculated from the chemical shifts at 298 and 258 K, according to equation (1).

$$\Delta\delta/\Delta T = \frac{\delta_{298} - \delta_{258}}{298 - 258} \qquad (1)$$

In the cases where the NH signals overlapped with aromatic signals, their chemical shifts at low temperature were confirmed with COSY experiments (data not shown).

Δδ/ΔT coefficients (Table S1) close to −3 ppb/K are indicative of NHs either completely exposed to the solvent or completely shielded, while values substantially higher (in absolute value, |Δδ/ΔT|) suggest the formation of H-bonds of intermediate strength. Moreover, for NHs forming H-bonds, similar coefficients at different concentrations indicate the formation of intramolecular H-bonds, while coefficients that significantly depend on the concentration indicate participation on intermolecular H-bonds.

Table S1: Δδ/ΔT coefficients (from 298 to 250 K).

**Compound**	**Concentration**	**Δδ/ΔT (ppb/K)**			
		**NH1**	**NH2**	**NH3**	**NH4**
**1b**	*50 mM*	-7			
	20 mM	-4			
	2.5 mM	-3			
	0.5 mM	-2			
**2b**	*20 mM*	-12	-15		
	2.5 mM	-3	-9		
	0.5 mM	-2	-8		
**3**	*20 mM*	-17	-12	-13	
	2.5 mM	-9	-12	-12	
	0.5 mM	-5	-11	-10	
	0.1 mM	-3	-10	-9	
**4**	*20 mM*	-7	-7	-4	-3
	2.5 mM	-2	-6	-4	-3
	0.5 mM	0	-6	-4	-3
**5**	*25 mM*	-19	-16	-10	
	2.5 mM	-19	-17	-11	
	0.5 mM	-19	-17	-11	

The NHs of compounds **1b**, **2b** and **3** are all susceptible to form H-bonds in certain conditions. The |Δδ/ΔT| value of the NH of compound **1b** slightly increases with concentration, which is indicative of the formation of weak intermolecular H-bonds. This compound showed little aggregation, so the concentration had to be increased to 50 mM to obtain a Δδ/ΔT indicative of significant aggregation.

In the case of compound **2b**, the $\Delta\delta/\Delta T$ of NH1 depends more on concentration than that of NH2, which indicates that NH1 forms intermolecular H-bonds and NH2 forms intramolecular H-bonds probably reinforced with aggregation.

In the case of compound **3**, the $\Delta\delta/\Delta T$ of NH1 depends more on concentration than those of NH2 and NH3, which indicates that NH1 forms intermolecular H-bonds while NH2 and NH3 form intramolecular H-bonds. Due to strong aggregation by this compound, a concentration as low as 0.1 mM was required to observe negligible aggregation (*i.e.,* $|\Delta\delta/\Delta T(NH1)| \leq 3$ ppb/K).

Compound **4** afforded low $|\Delta\delta/\Delta T|$ values for the four NHs, which could come from completely exposed or strongly shielded NHs. The chemical shifts (δ) of the signals allowed differentiation between these two options. The δ of NH2 is much higher than that of NH1, and the δ of NH3 and NH4 are also substantially higher than those of the reference thiourea **15** (Figure S2), which indicates that NH2, NH3 and NH4 are involved in a strong intramolecular H-bonding network and NH1 is completely exposed to the solvent. This is further confirmed from the observation that NH1 is the only signal showing a $|\Delta\delta/\Delta T|$ that increases with concentration (due to weak intermolecular H-bonding at high concentration).

In the case of compound **5**, high $\Delta\delta/\Delta T$ values that do not depend on the concentration indicate that the three NHs are involved in intramolecular H-bonds.

The $\Delta\delta/\Delta T$ values of NH1 at 20 mM also indicate that aggregation of the compounds increases in the order **1b** < **4** < **2b** < **3** (**5** is not considered because NH1 forms an intramolecular H-bond).

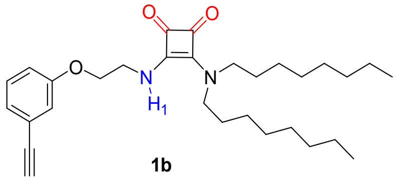

1b

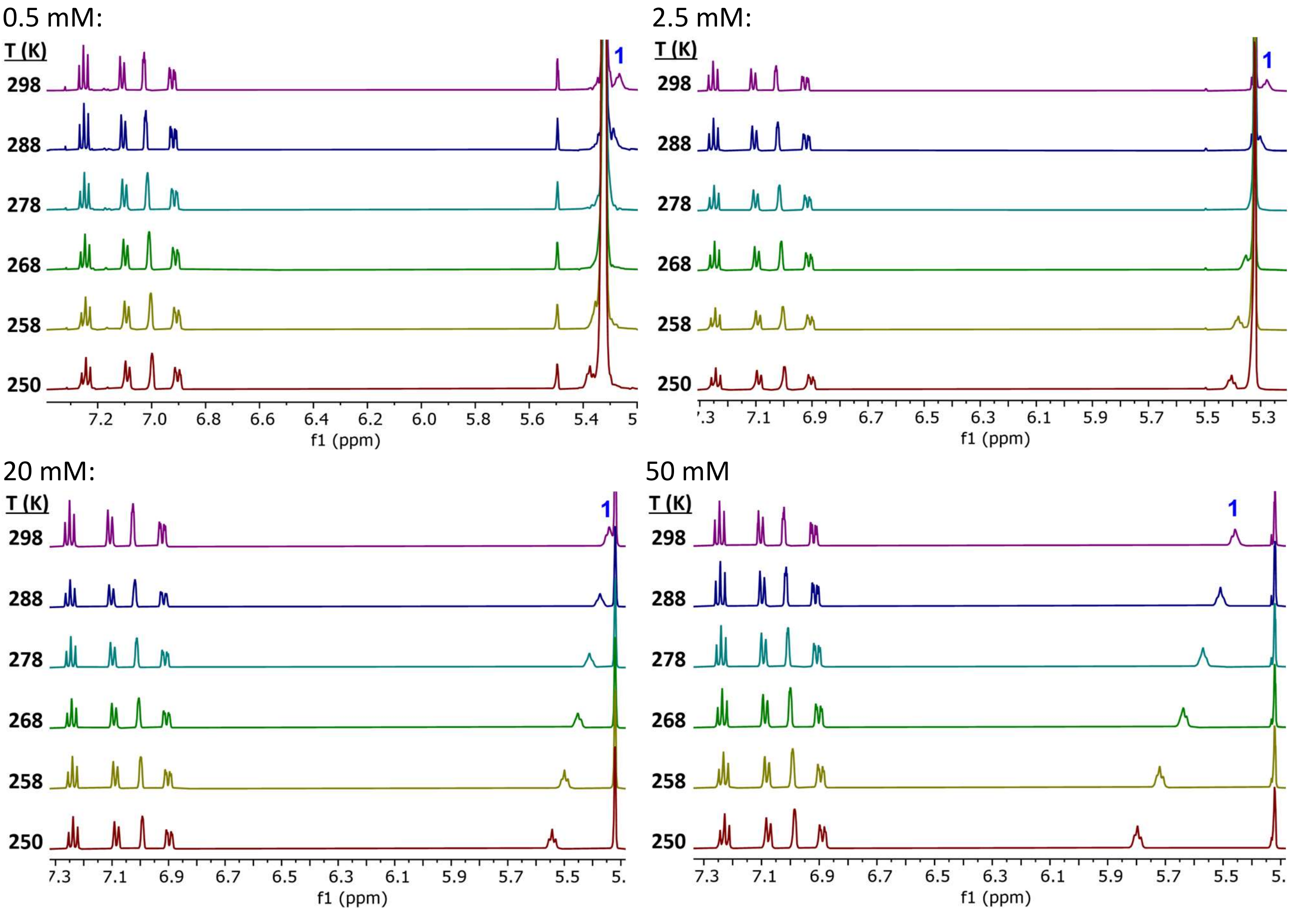

Figure S18. Selected regions of the ^{1}H NMR spectra (500 MHz, CD_2Cl_2) of compound **1b** at different temperatures, for four different concentrations.

0.5 mM:

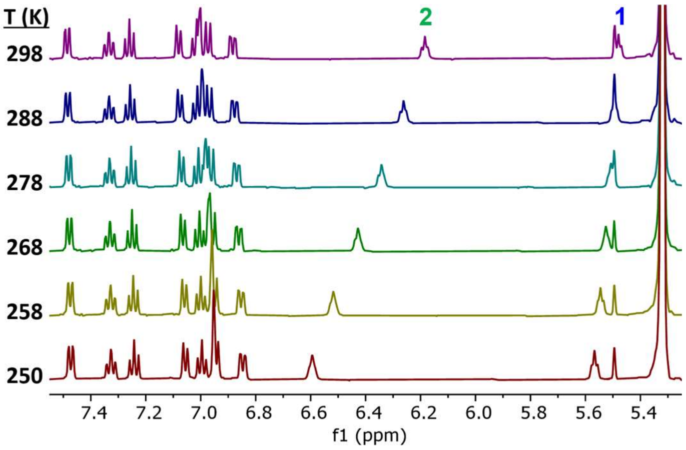

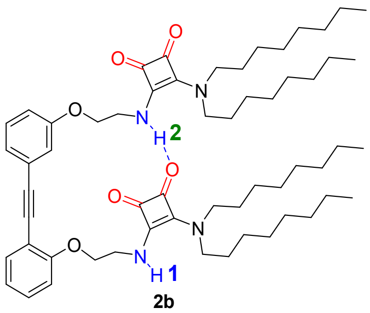

2b

2.5 mM:

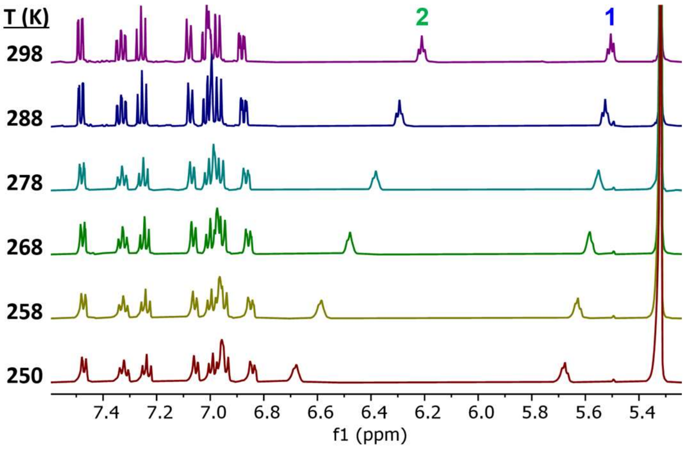

20 mM:

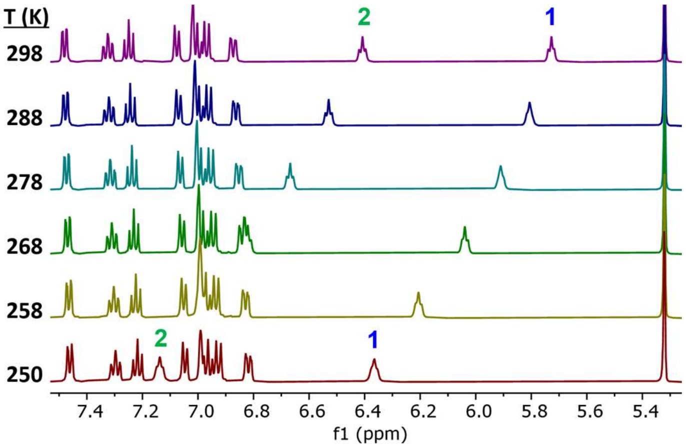

Figure S19. Selected regions of the ^{1}H NMR spectra (500 MHz, CD_2Cl_2) of compound **2b** at different temperatures, for three different concentrations.

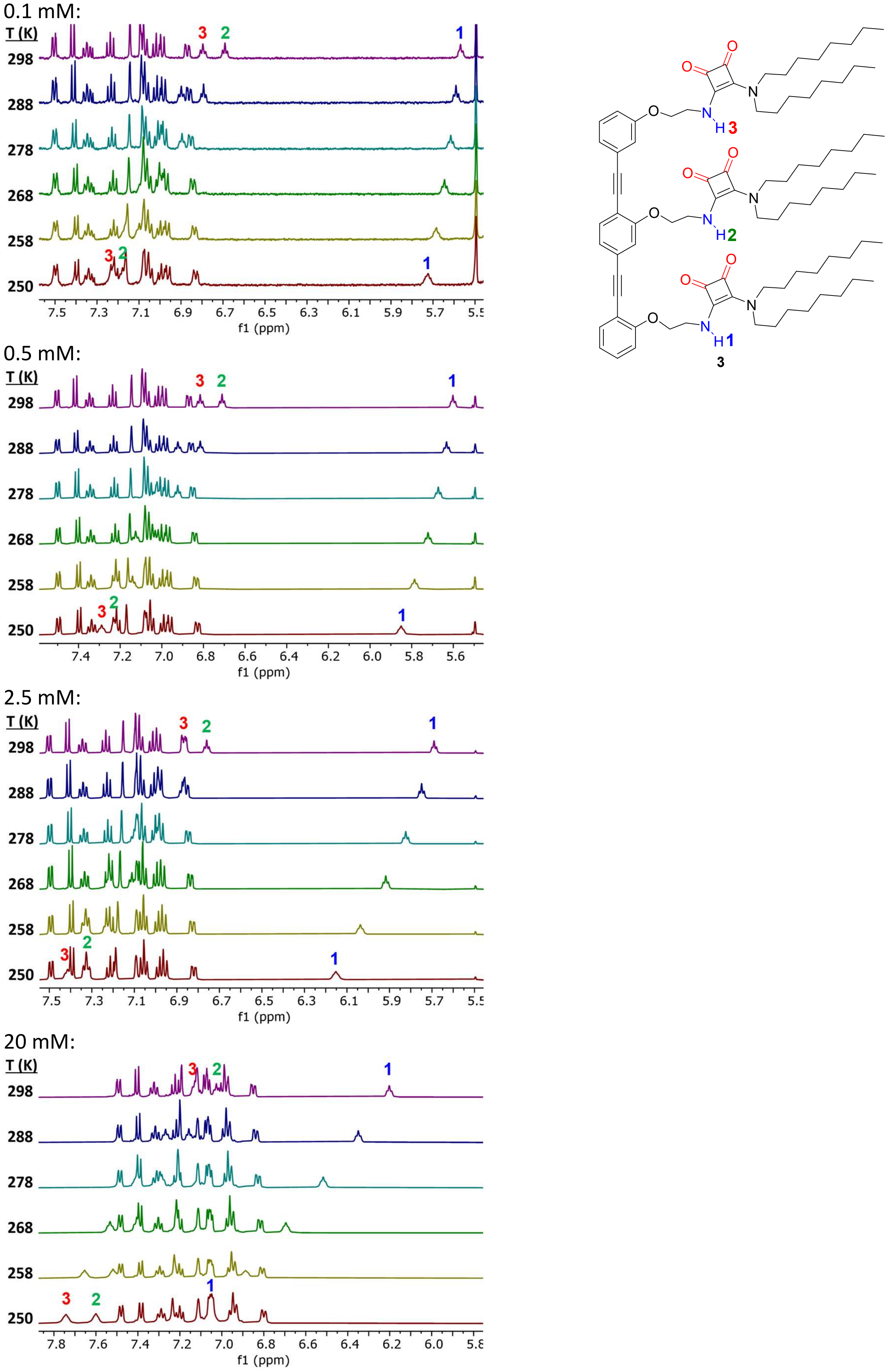

Figure S20. Selected regions of the ^{1}H NMR spectra (500 MHz, CD_2Cl_2) of compound **3** at different temperatures, for four different concentrations.

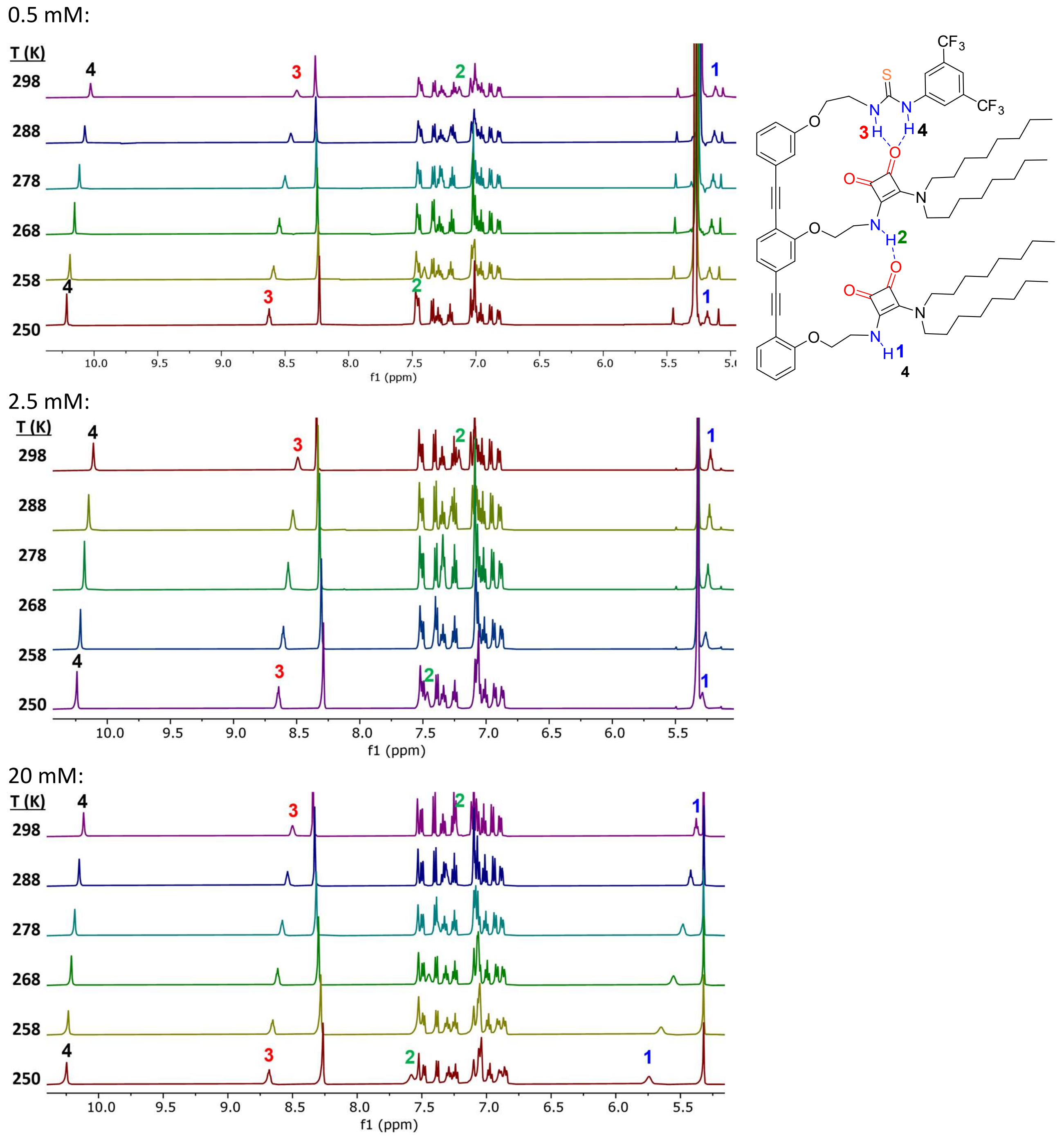

Figure S21. Selected regions of the ^1H NMR spectra (500 MHz, CD_2Cl_2) of compound **4** at different temperatures, for three different concentrations.

0.5 mM:

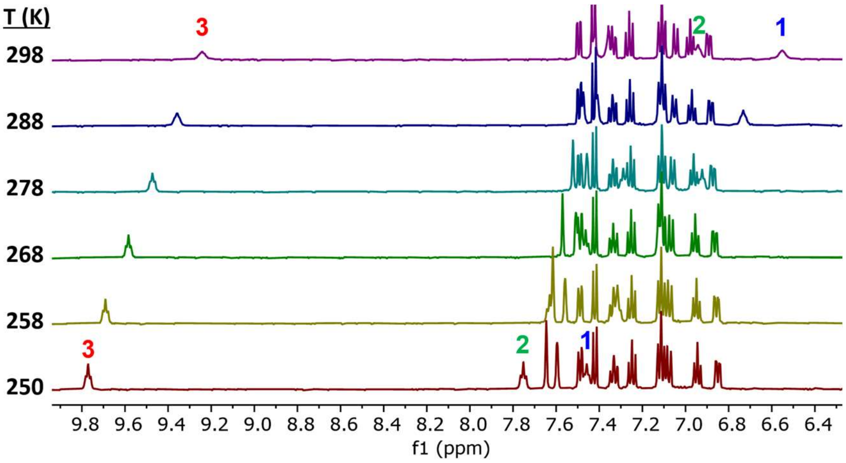

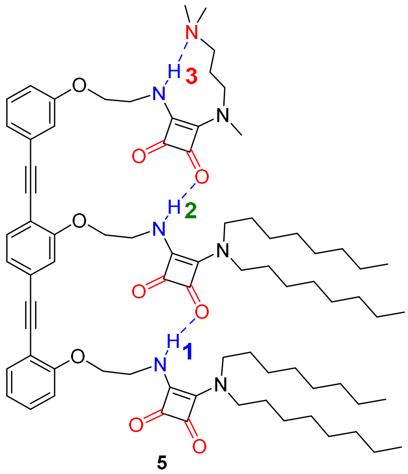

5

2.5 mM:

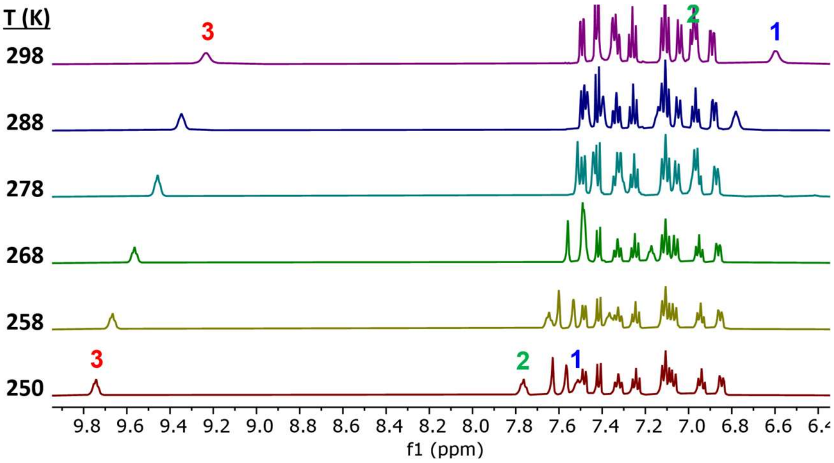

25 mM:

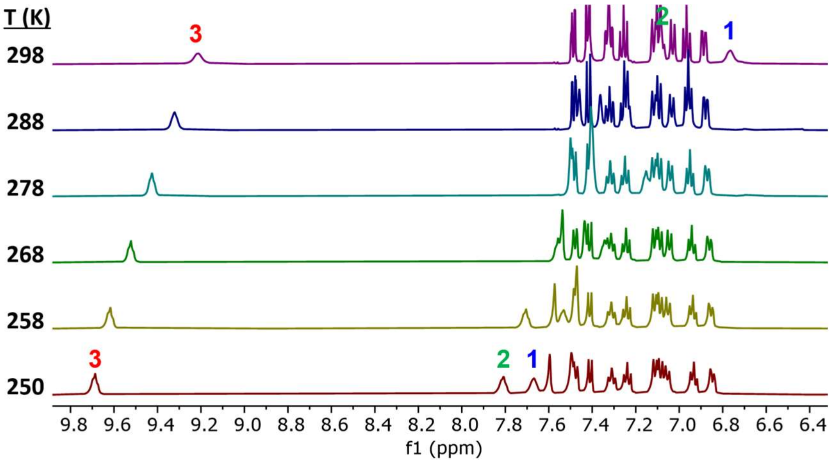

Figure S22. Selected regions of the ^{1}H NMR spectra (500 MHz, CD_2Cl_2) of compound **5** at different temperatures, for three different concentrations.

4. ^{1}H NMR experiments in $(CD_3)_2CO$ for compounds 1b, 2b, 3, 4 and 5

The conformational properties of compounds **1b**, **2b**, **3**, **4** and **5** dissolved in $(CD_3)_2CO$ were studied by ^{1}H NMR spectroscopy. The spectra registered in pure $(CD_3)_2CO$ were referenced to the signal of $(CD_2H)(CD_3)CO$ at 2.05 ppm.

Conformational analysis (Figure S23): For compounds **1b**, **2b**, **3** and **4**, the higher chemical shift of NH2 compared to NH1 reveals the formation of a *parallel* intramolecular H-bonding network, with NH1 exposed and NH2 bound. Similarly, NH3 of compound **3** is also involved in the intramolecular H-bonding network. In the case of compound **4**, the chemical shifts of signals NH3 and NH4 are noticeably higher than the analogous NH signals of the reference compound **15**, revealing that the thiourea unit of **4** is intramolecularly hydrogen bonded to the adjacent SQ. This explains why compound **4** shows the highest chemical shift for NH2, since the thiourea moiety probably induces the strongest H-bonding network. The conformation of **5** is less clear, however.

Intramolecular cooperativity: The binding constants of the compounds with DMSO-d_6 were obtained from the titrations by fitting of the change in the NH1 signals to a 1:1 binding model (Figure S34). These show the same trend in NH1 acidity as observed in CD_2Cl_2 (**4** > **3** > **2b** > **1**), but in $(CD_3)_2CO$ the differences between the *K* values for each compound are much lower. Since acetone is a more polar solvent than DCM (stronger H-bond acceptor), it leads to weaker intramolecular H-bonding networks and, as a consequence, the intramolecular cooperativity effects are weaker in this solvent.

4.1. Assignment of the ^{1}H NMR spectra in $(CD_3)_2CO$

The ^{1}H NMR spectra of compounds **1b** and **15** were easily assigned based on the multiplicity and chemical shifts of the signals observed.

The ^{1}H NMR spectra of compounds **2b**, **3**, **4** and **5** were assigned based on the multiplicity and chemical shifts of the signals observed, as well as the COSY and NOESY spectra.

- Each aromatic ring from the *oligo*-phenylene ethynylene rigid-rods showed a characteristic pattern on the aromatic region of the diagonal of the COSY spectra, which permitted the assignment of the corresponding signals.
- The signals from each CH_2CH_2NH system were connected from COSY cross-peaks.
- Each CH_2CH_2NH system was connected to an aromatic ring of the *oligo*-phenylene ethynylene rigid-rods based on NOESY cross-peaks, which permitted the unequivocal assignment of all the CH_2CH_2NH signals.
- The signals of the C_2H_5, C_8H_{17} and $C_3H_6NH(CH_3)_2$ squaramide substituents showed characteristic multiplicities and chemical shifts, and showed the expected COSY cross-peaks.
- The signals of the CH_3 squaramide substituents and the 3,5-$C_6H_3(CF_3)_2$ thiourea substituents appeared as singlets and showed characteristic chemical shifts.

The spectra were assigned using numbers for the NH signals (1-4), capital letters with a subindex for the aromatic signals from the *oligo*-phenylene ethynylene rigid-rods (A-$C_{1\text{-}4}$) and lowercase letters for the rest of CH signals (a-n).

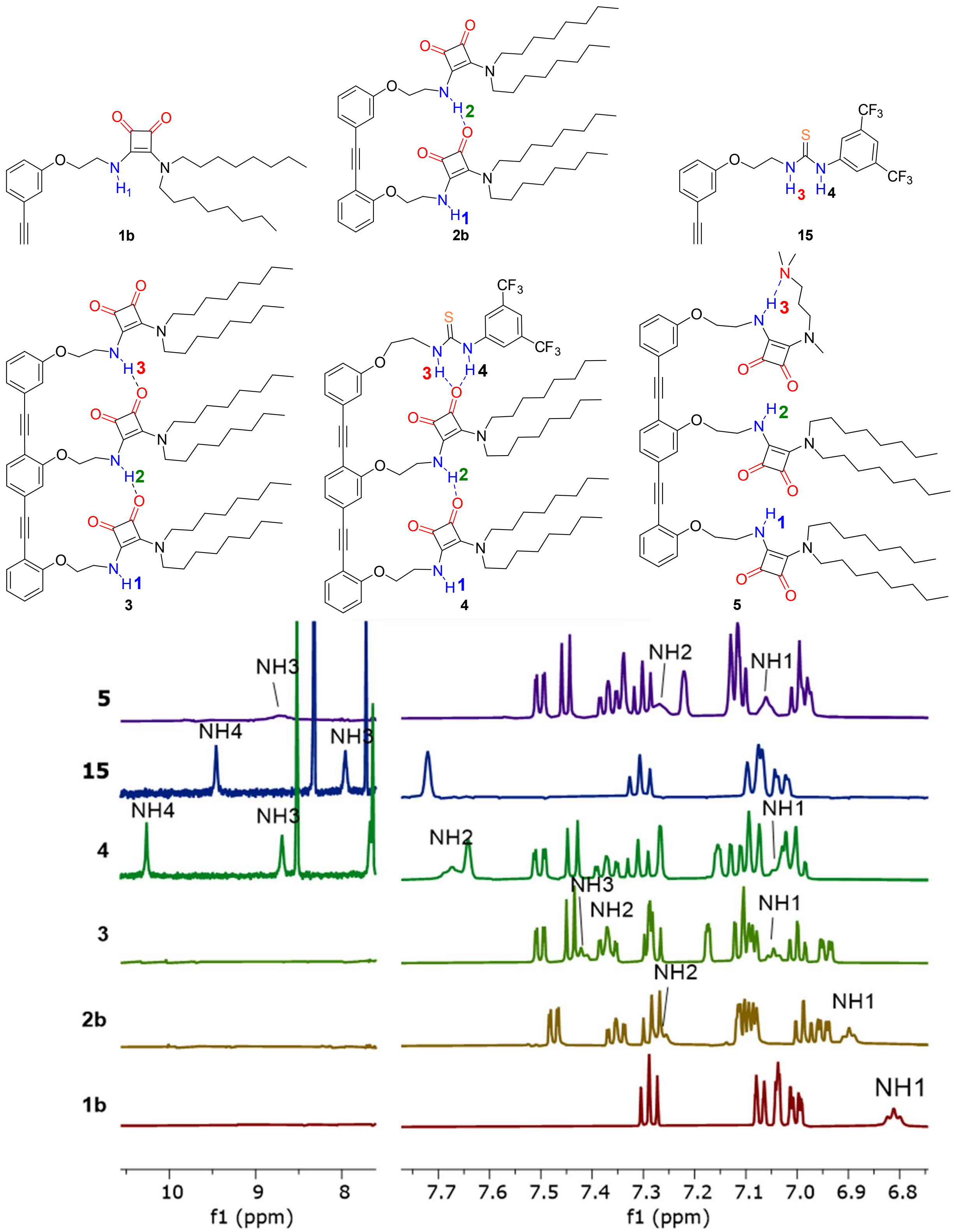

Figure S23. Selected regions of the ^{1}H NMR spectra (500 MHz, 298 K, $(CD_3)_2CO$) of compounds **1b**, **2b**, **3**, **4** and **5** (2 mM).

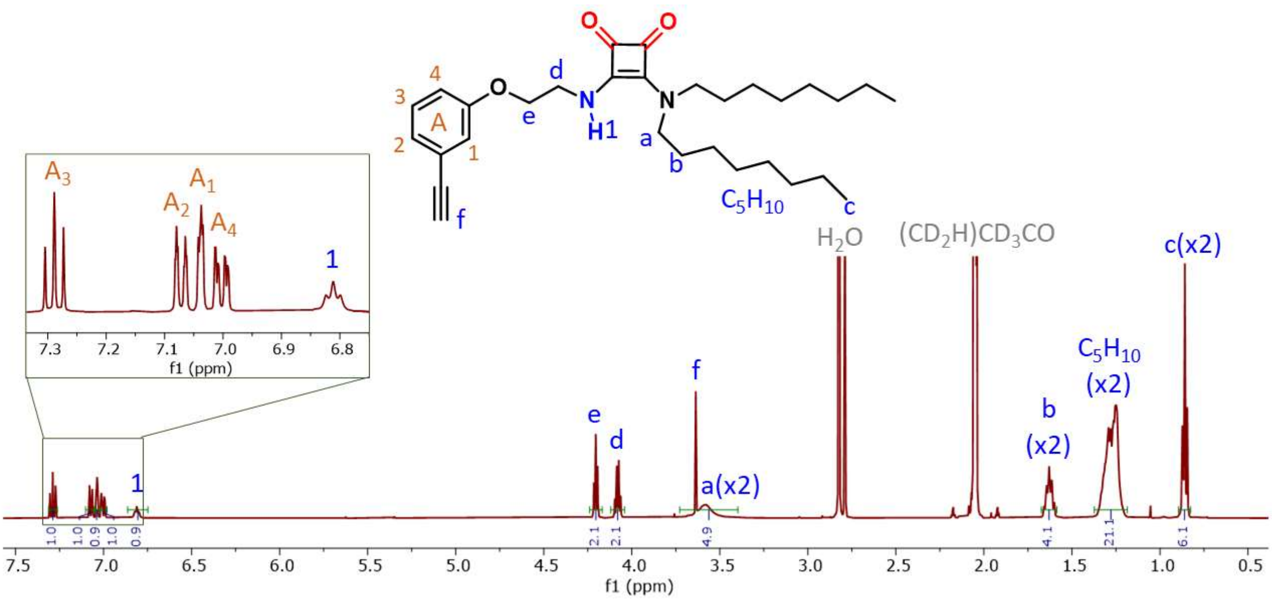

Figure S24. Assigned ^{1}H NMR spectrum (500 MHz, 298 K, $(CD_3)_2CO$) of compound **1b** (2.5 mM).

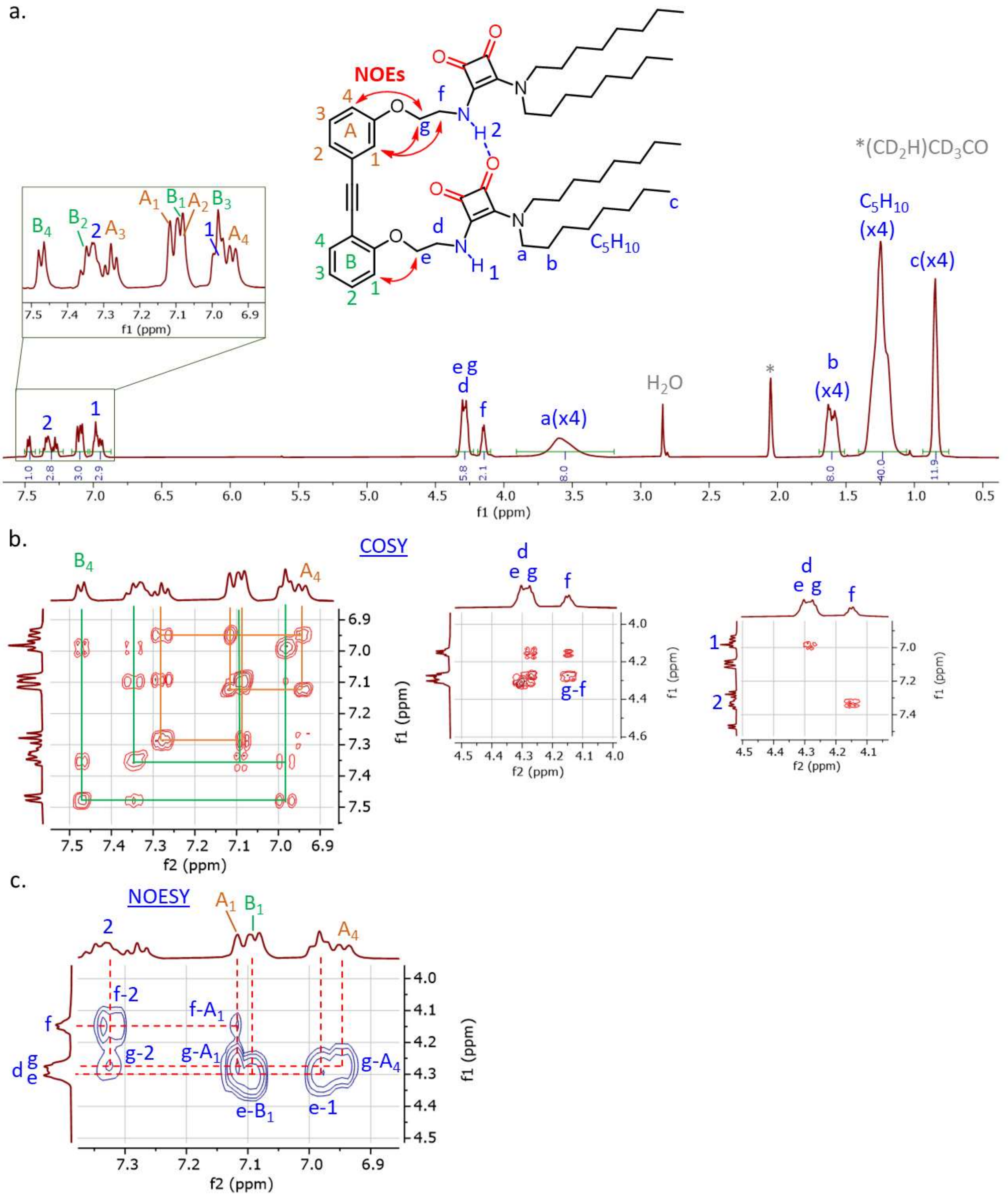

Figure S25. Assigned ^{1}H NMR spectrum (500 MHz, 298 K, $(CD_3)_2CO$) of compound **2b** (10 mM) (a) and selected regions of the COSY (b) and NOESY (c) spectra.

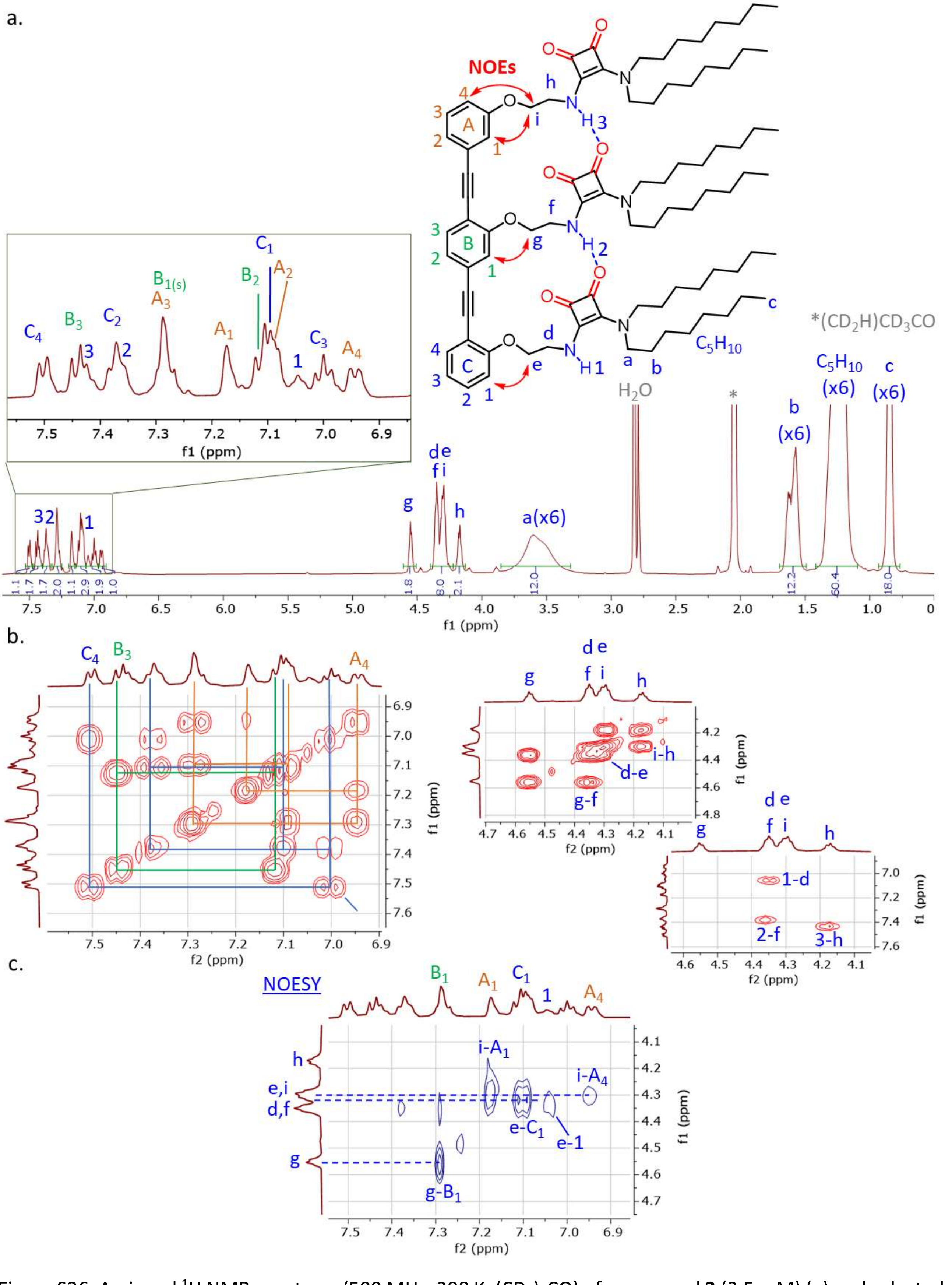

Figure S26. Assigned ^{1}H NMR spectrum (500 MHz, 298 K, $(CD_3)_2CO$) of compound **3** (2.5 mM) (a) and selected regions of the COSY (b) and NOESY (c) spectra.

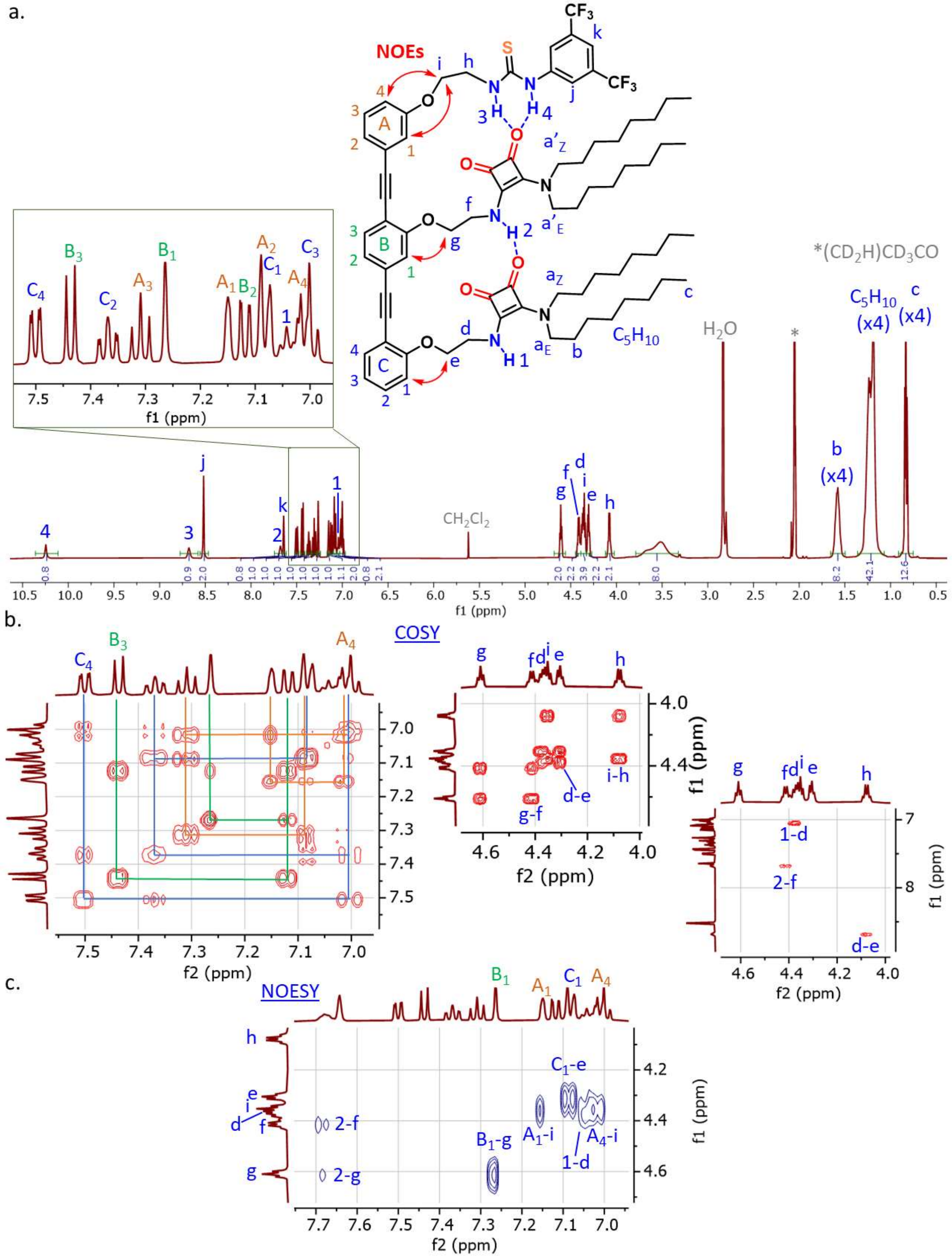

Figure S27. Assigned ^{1}H NMR spectrum (500 MHz, 298 K, $(CD_3)_2CO$) of compound **4** (2.5 mM) (a) and selected regions of the COSY (b) and NOESY (c) spectra.

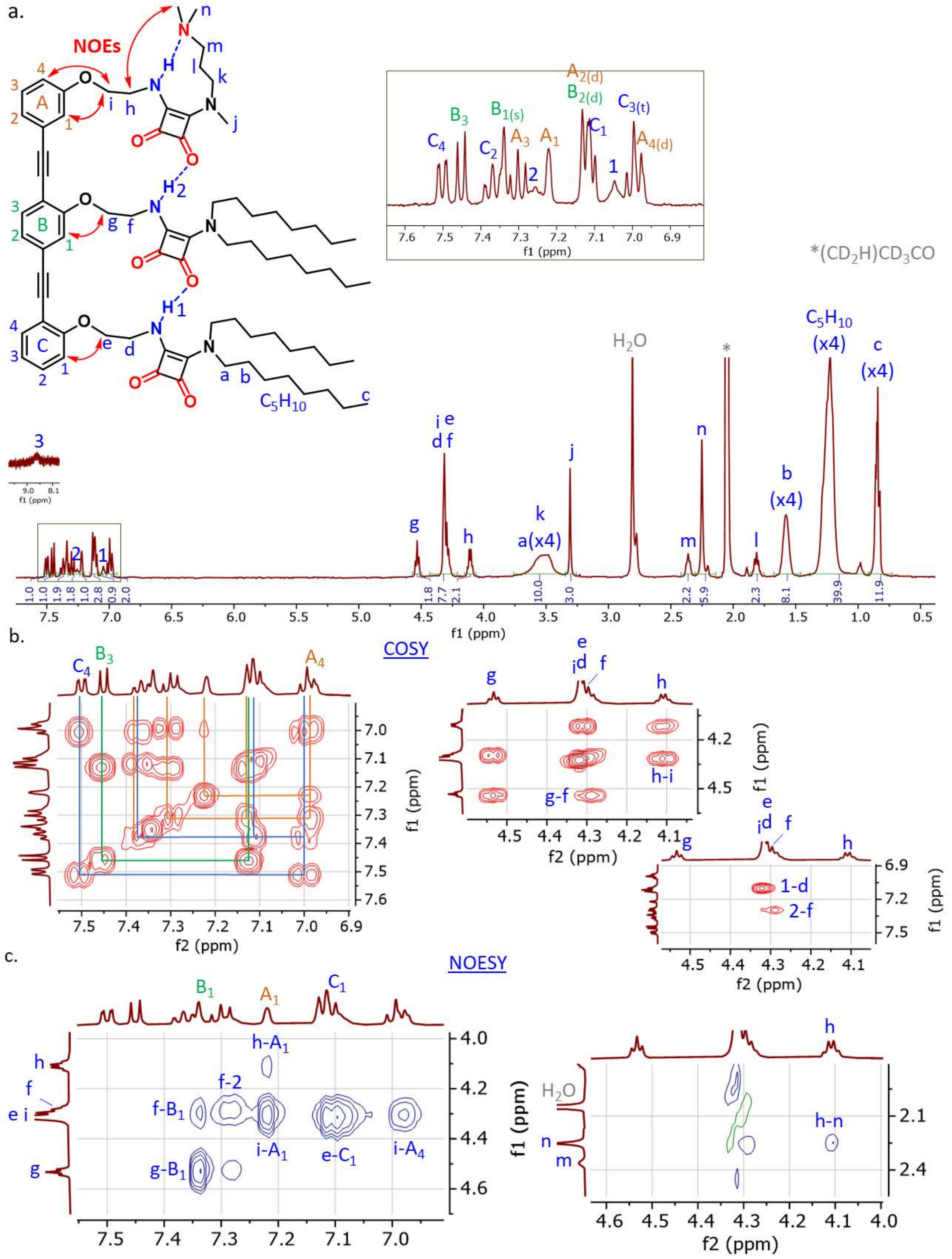

Figure S28. Assigned ^{1}H NMR spectrum (500 MHz, 298 K, $(CD_3)_2CO$) of compound **5** (2.5 mM) (a) and selected regions of the COSY (b) and NOESY (c) spectra.

4.2. Titrations with DMSO-d_6 in $(CD_3)_2CO$

The effect of DMSO on the different NH signals was studied by adding increasing amounts of DMSO-d_6 into solutions of the compounds in CD_2Cl_2 at 298 K. Solutions of compounds **1b**, **2b**, **3**, **4** and **5** (2.5 mM, 0.6 mL) were prepared in CD_2Cl_2 and the ^1H NMR spectra were recorded. Then aliquots of DMSO-d_6 were added to the NMR tube and ^1H NMR spectra were recorded after each addition.

Additions of DMSO-d_6 : 5, 5, 10, 10, 10, 20, 30, 30, 60 and 60 µL.

In certain cases, the chemical shifts of the NH signals in presence of a concrete amount of DMSO were confirmed with COSY experiments.

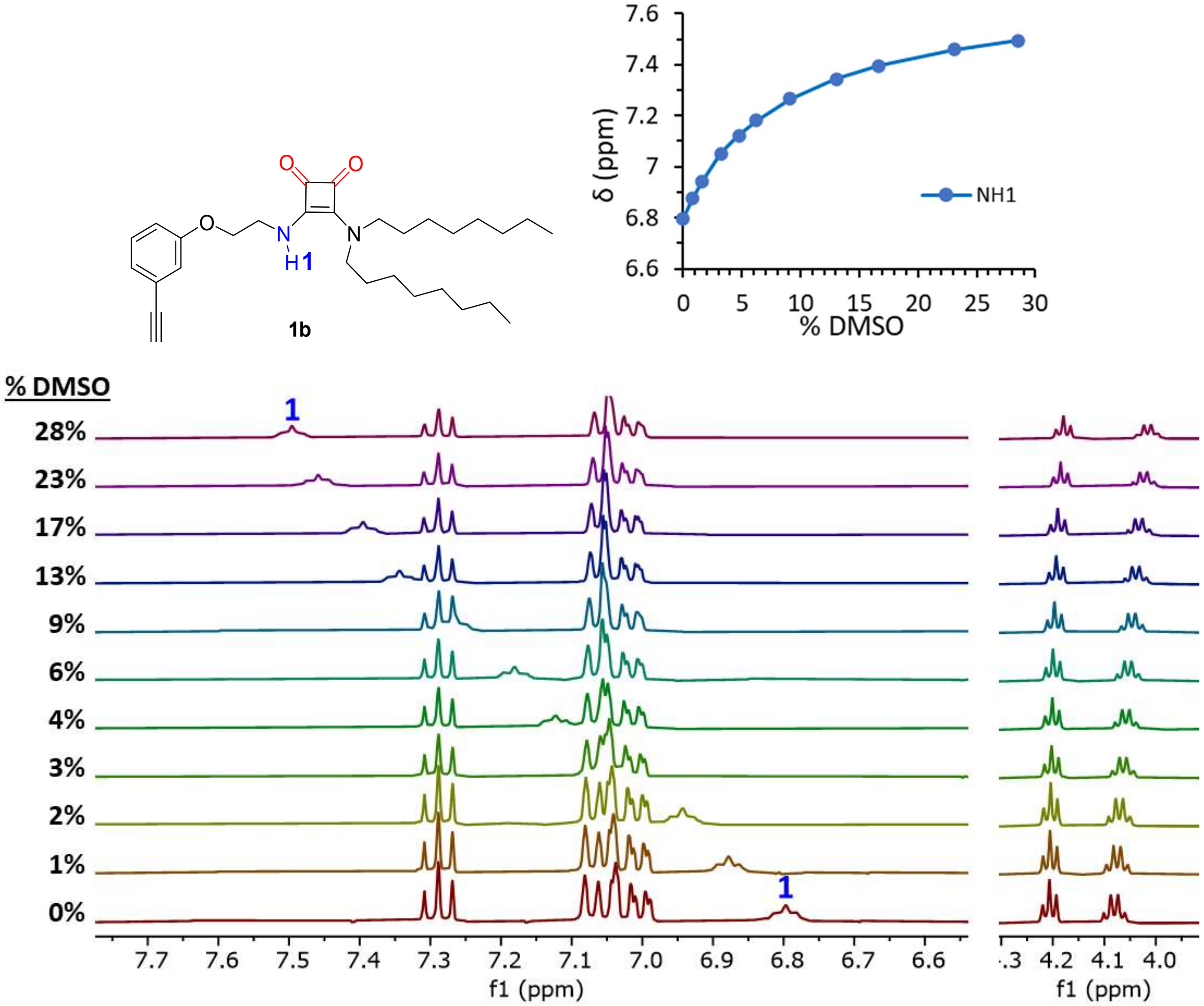

Figure S29. Selected region of the ^1H NMR spectra (400 MHz, 298 K, $(CD_3)_2CO$) from the titration of compound **1b** (initially 2 mM) with DMSO-d_6. The proportion of DMSO-d_6 is indicated as % v/v.

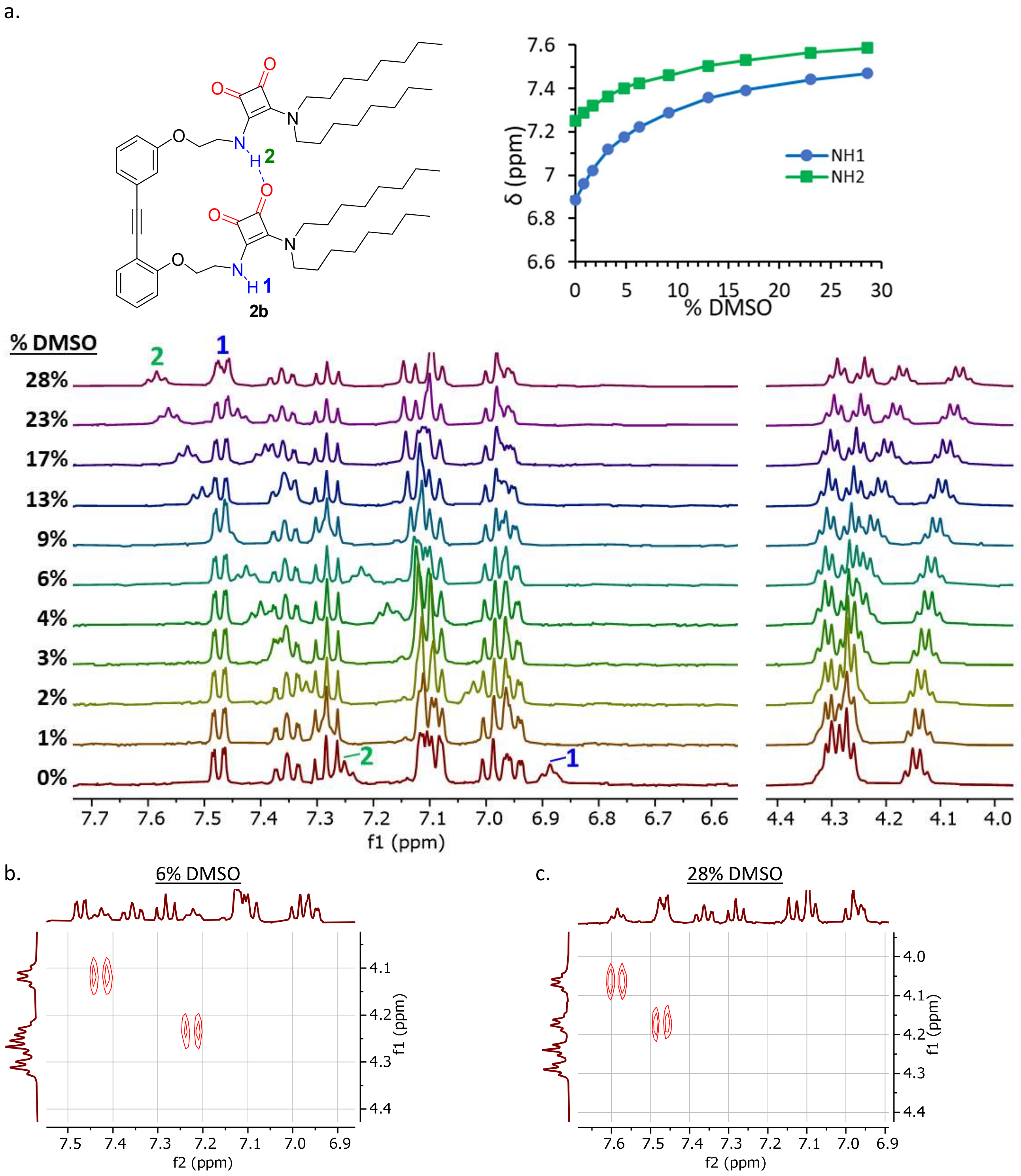

Figure S30. a. Selected region of the ^{1}H NMR spectra (400 MHz, 298 K, $(CD_3)_2CO$) from the titration of compound **2b** (initially 2 mM) with DMSO-d_6. The proportion of DMSO-d_6 is indicated as % v/v. b-c. Selected regions of the COSY spectrum in presence of 6% DMSO-d_6 (b) and 28% DMSO-d_6 (c) (the CH_2-NH cross-peaks reveal the chemical shifts of the NH signals).

a.

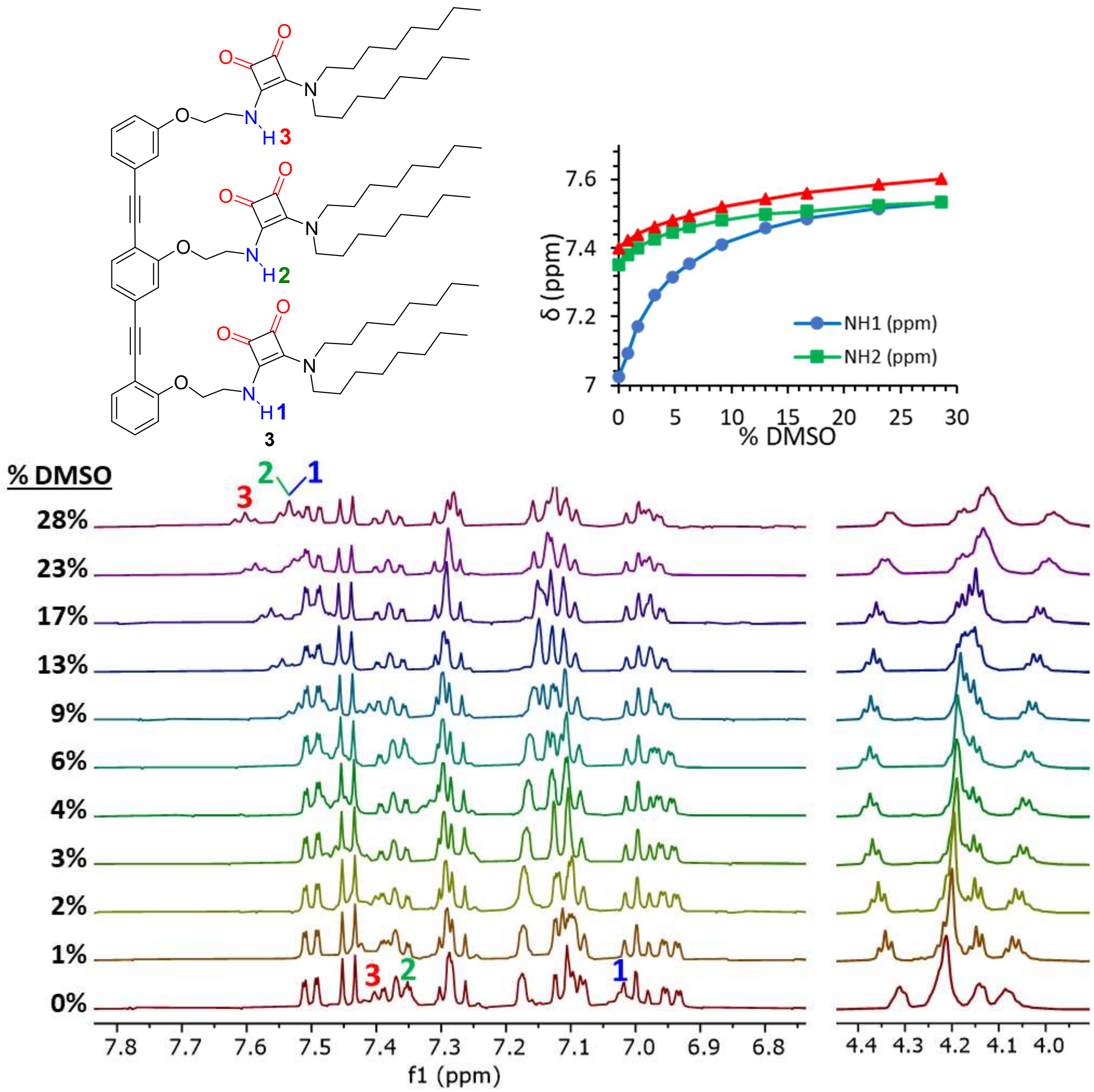

b.

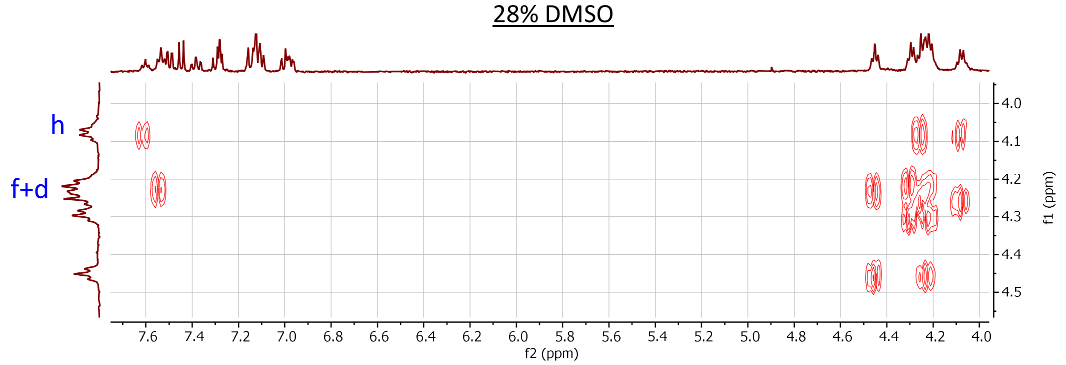

Figure S31. a. Selected region of the ^{1}H NMR spectra (400 MHz, 298 K, $(CD_3)_2CO$) from the titration of compound **3** (initially 2 mM) with DMSO-d_6. The proportion of DMSO-d_6 is indicated as % v/v. b. Selected region of the COSY spectrum in presence of a 28% DMSO-d_6.

a.

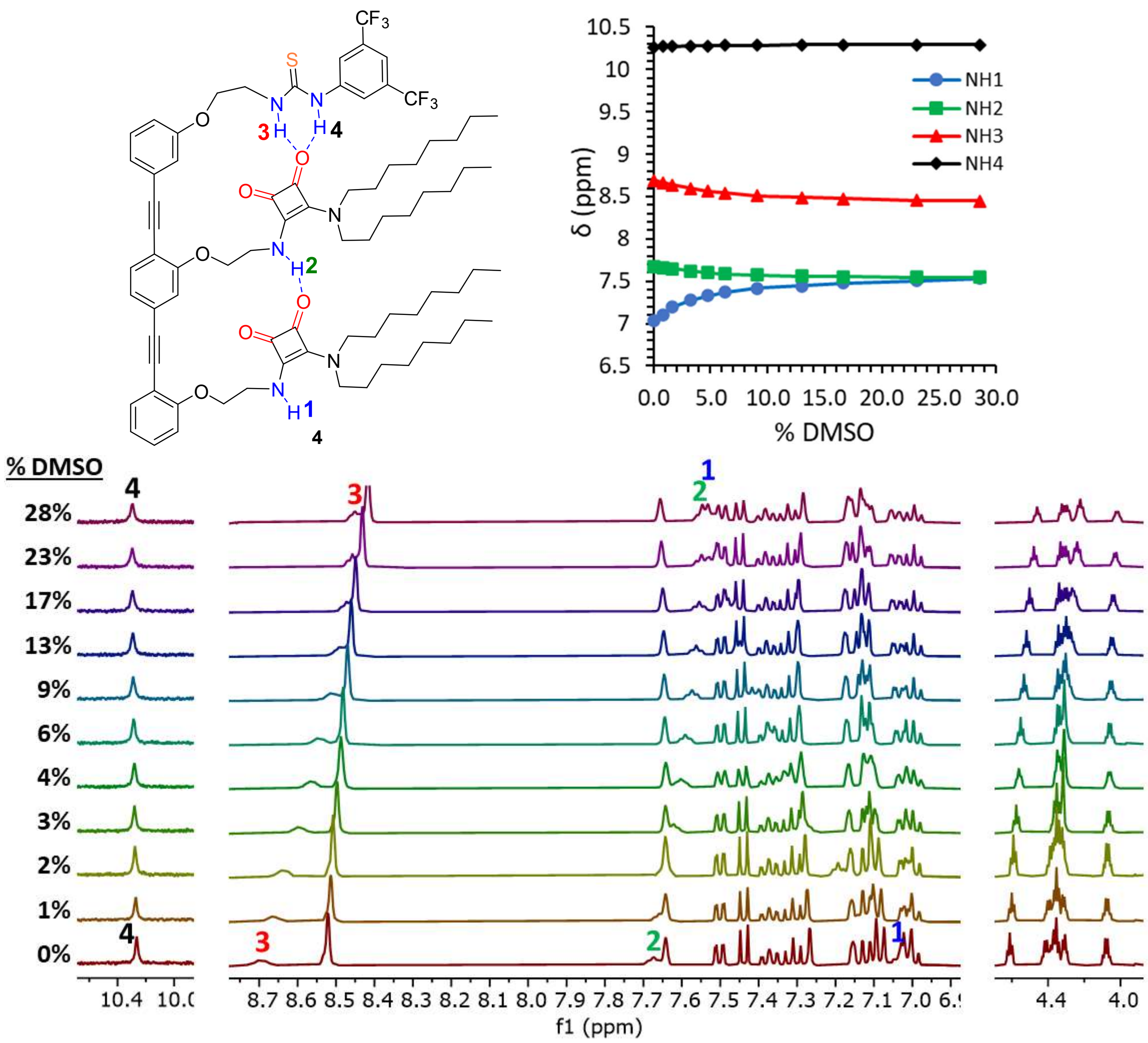

b.

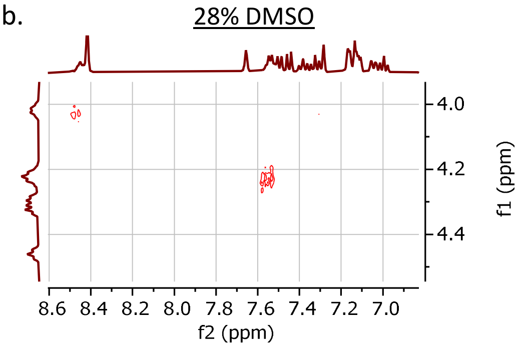

Figure S32. Selected region of the ^{1}H NMR spectra (400 MHz, 298 K, $(CD_3)_2CO$) from the titration of compound **4** (initially 2 mM) with DMSO-d_6. The proportion of DMSO-d_6 is indicated as % v/v. b. Selected region of the COSY spectrum in presence of 28% DMSO-d_6 (the CH_2-NH cross-peaks reveal the chemical shifts of the NH signals).

a.

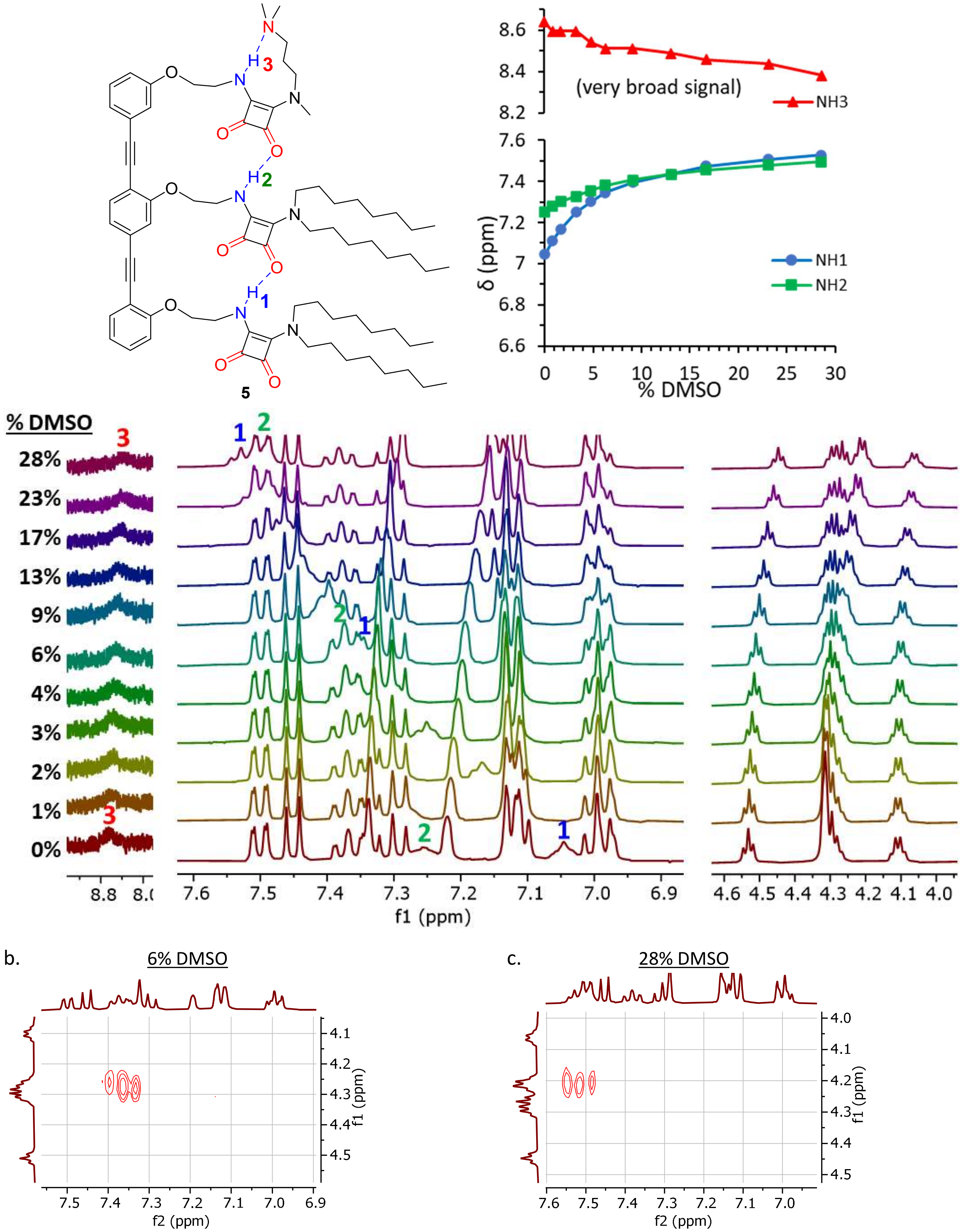

Figure S33. a. Selected region of the ^{1}H NMR spectra (400 MHz, 298 K, $(CD_3)_2CO$) from the titration of compound **5** (initially 2 mM) with DMSO-d_6. The proportion of DMSO-d_6 is indicated as % v/v. b-c. Selected regions of the COSY spectrum in presence of 6% DMSO-d_6 (b) and 28% DMSO-d_6 (c) (the CH_2-NH cross-peaks reveal the chemical shifts of the NH signals).

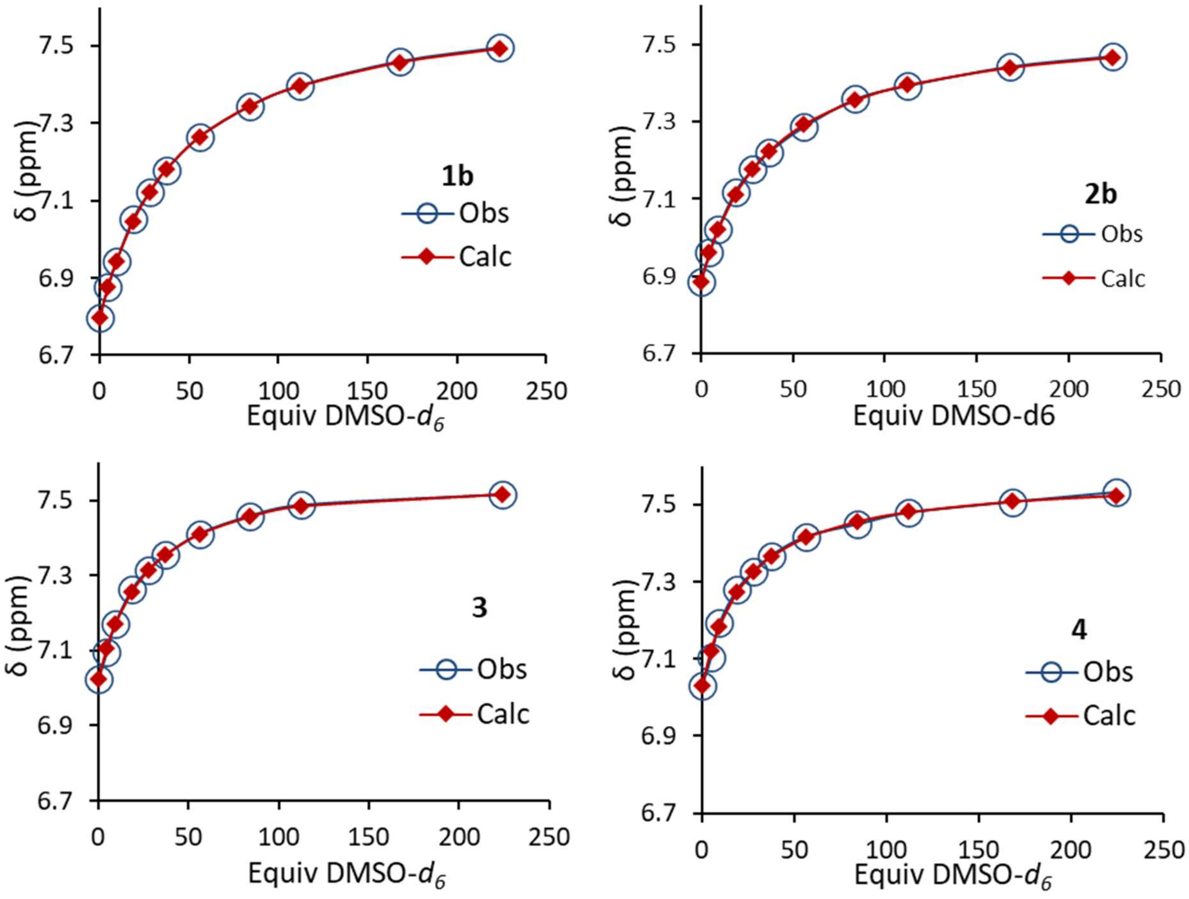

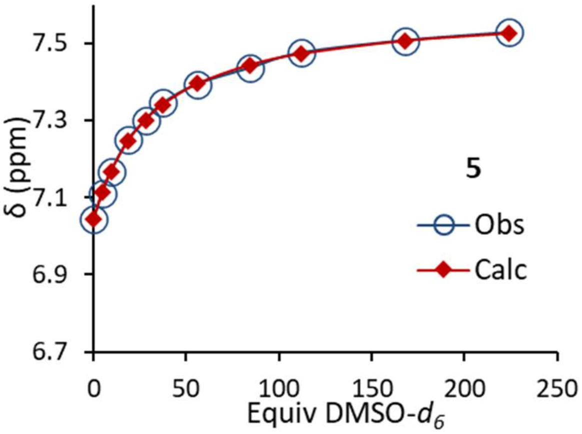

Compound	*K* (M^{-1})
**1b**	8.6
**2b**	10.2
**3**	14.4
**4**	16.7
**5**	12.1

Figure S34. Variation of the chemical shift of NH1 during the titrations of **1b**, **2b**, **3**, **4** and **5** (2.5 mM) with DMSO-d_6 at 298 K in $(CD_3)_2CO$. The binding constants, shown in the table, were obtained from the fitting to a 1:1 binding model with http://app.supramolecular.org/bindfit/ (these values are indicative of the relative acidity of NH1). c. Observed (obs) and calculated (calc) binding curves.

4.3. VT experiments in $(CD_3)_2CO$

VT experiments (at 2 mM) yielded temperature coefficients between -5 and -8 ppb/K (Table S2). Only in NH3 of **5** a slightly higher coefficient is observed (-10 ppb/K), due to the formation of the intramolecular H-bond with the amino group. Since the ^{1}H NMR spectra in $(CD_3)_2CO$ strongly suggest that some NHs form intramolecular H-bond and others are completely exposed to the solvent, we conclude that VT coefficients cannot differentiate between different types of H-bonding in this solvent.

Table S2: Temperature coefficients ($\Delta\delta/\Delta T$) calculated for the temperature range 298-258 K.

Compound	NH1	NH2	NH3	NH4
**1b**	-0.006			
**2b**	-0.007	-0.008		
**3**	-0.006	-0.006	-0.008	
**4**	-0.007	-0.006	-0.006	-0.005
**5**	-0.006	-0.005	-0.010	

$$\Delta\delta/\Delta T = \frac{\delta_{298} - \delta_{258}}{298 - 258} \quad (1)$$

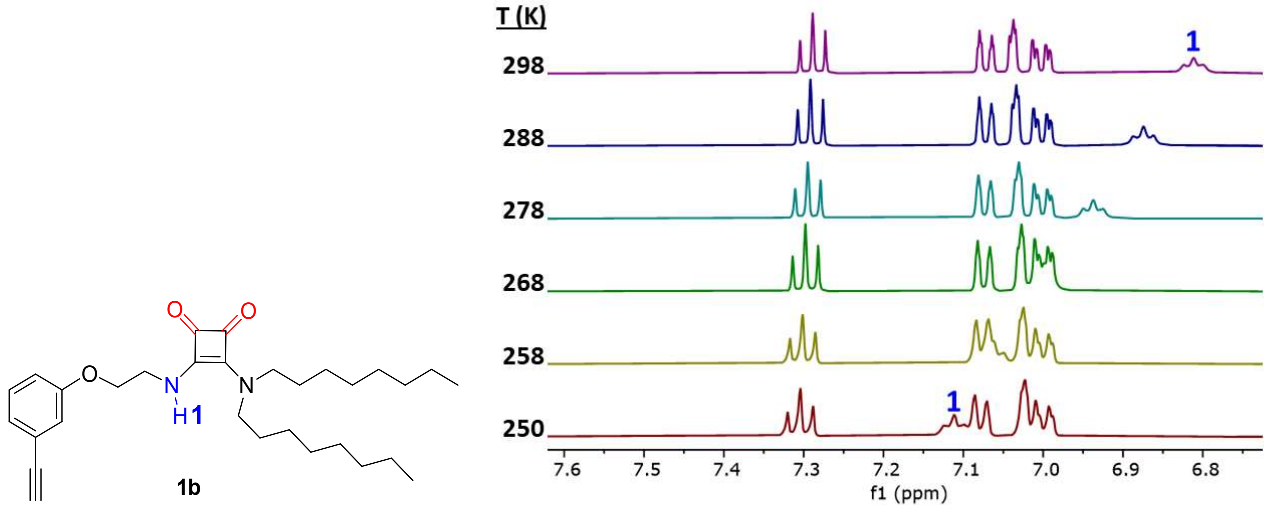

Figure S35. Selected regions of the ^{1}H NMR spectra (500 MHz, $(CD_3)_2CO$) of compound **1b** (2 mM) at different temperatures.

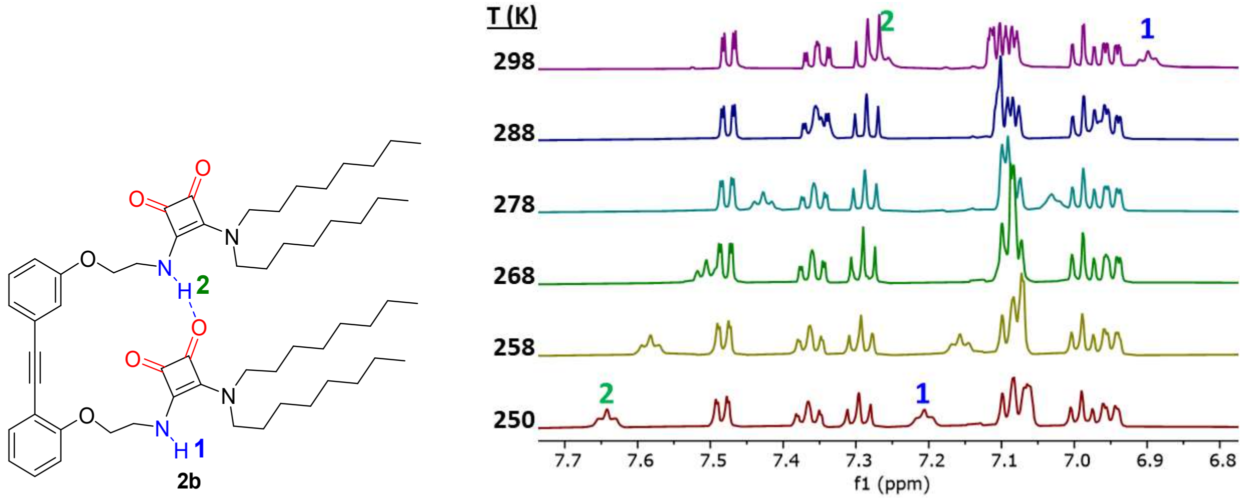

Figure S36. Selected regions of the ^{1}H NMR spectra (500 MHz, $(CD_3)_2CO$) of compound **2b** (2 mM) at different temperatures.

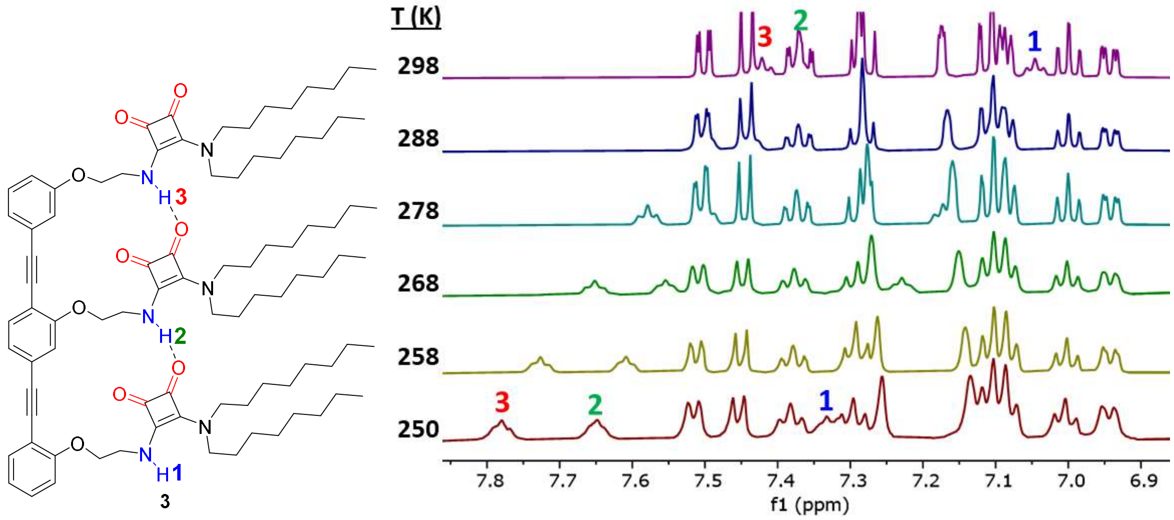

Figure S37. Selected regions of the ^{1}H NMR spectra (500 MHz, $(CD_3)_2CO$) of compound **3** (2 mM) at different temperatures.

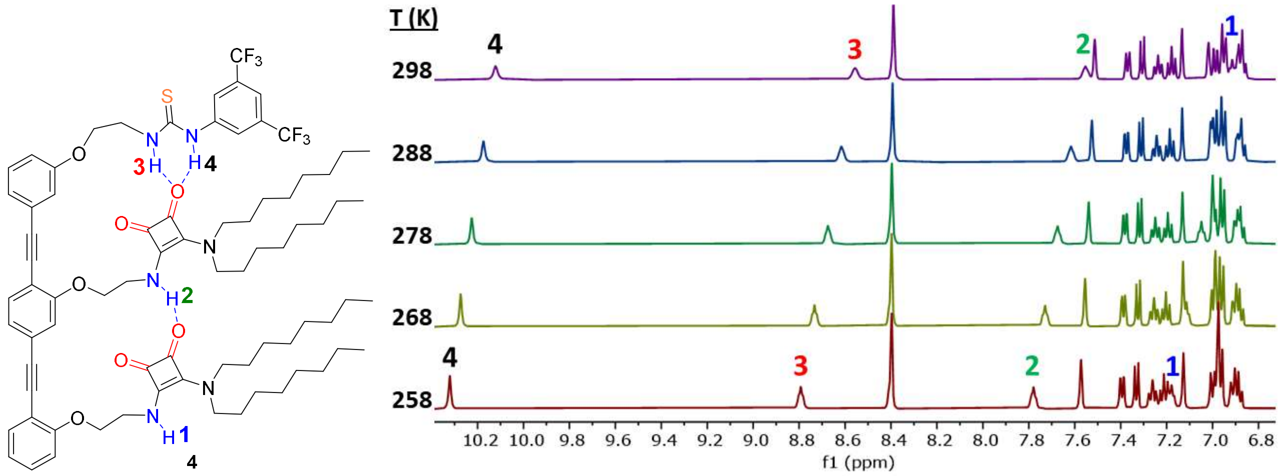

Figure S38. Selected regions of the ^{1}H NMR spectra (500 MHz, $(CD_3)_2CO$) of compound **4** (2 mM) at different temperatures.

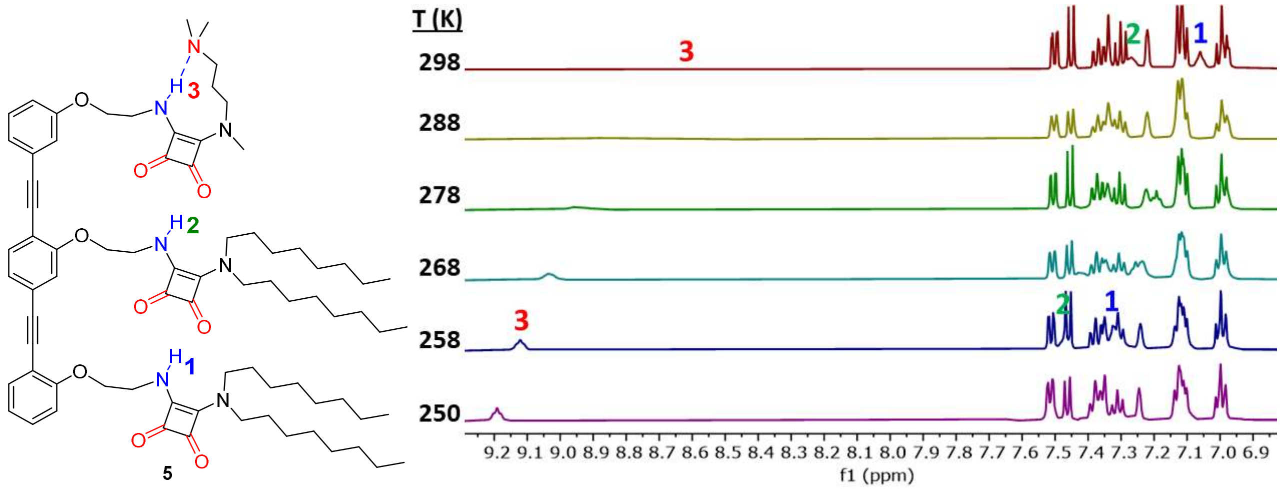

Figure S39. Selected regions of the ^{1}H NMR spectra (500 MHz, $(CD_3)_2CO$) of compound **5** (2 mM) at different temperatures.

5. X-ray crystal structures

5.1. Crystallographic data

X-ray diffraction data for compounds **4** and **5** have been published previously.[S2]

Data collection: X-ray diffraction data for compounds **1a**, **2a** and **3** were collected using a dual wavelength Rigaku FR-X rotating anode diffractometer using CuKα (λ = 1.54184 Å) radiation, equipped with an AFC-11 4-circle kappa goniometer, VariMAX™ microfocus optics, a Hypix-6000HE detector and an Oxford Cryosystems 800 plus nitrogen flow gas system, at temperatures of 240K, 100K and 100K, respectively. X-ray diffraction data for compound **2b** were collected using a Rigaku Supernova microfocus sealed tube diffractometer using Mo-Kα (0.71073 Å) radiation, equipped with a 4-circle kappa goniometer, X-ray focussing optics, an Eos CCD detector and an Oxford Cryosystems 700 nitrogen flow gas system, at a temperature of 100 K. Data were collected and reduced using CrysAlisPro v43.[S4] Absorption correction was performed using empirical methods (SCALE3 ABSPACK) based upon symmetry-equivalent reflections combined with measurements at different azimuthal angles.

Crystal structure determination and refinements: The crystal structures were solved and refined against all F^2 values using the SHELX and Olex2 suite of programmes.[S5,S6] All non-hydrogen atoms were refined anisotropically. Hydrogen atoms were placed in calculated positions and refined using idealised geometries and assigned fixed isotropic displacement parameters.

In the case of **2a**, **2b** and **3**, crystallographic disorder was present, with crystallographic translation along the “alkyne axis” of the OPE. On initial solution, the electron density appears to be polymeric, with an apparent infinite chain of SQ-phenylalkyne fractions. Free refining the occupancies of the alkyne moieties in the models for **2a** and **2b** gave values close to 50% (partial occupancy 0.527(9) and 0.505(8) for **2a**; 0.512(18) and 0.453(18) for **2b**). Free refining the occupancies of the alkyne moiety in the models for **3** gave values close to 67% (partial occupancy 0.635(2) and 0.656(10)). These partial occupancies were constrained when refining the model, such that the alkyne occupancies were 50% and 67% for those sites, respectively, and the sum of the refined SQ moieties was 100%. In order to ensure the correct connectivity for each model, the structures were modelled such that each atomic position was split into multiple parts (**2a** and **2b**, two parts; **3** three parts), with these parts refined to have equivalent atomic coordinates and atomic displacement parameters. Bonds were then added between neighbouring symmetry equivalent positions in such a way that the dimeric and trimeric molecular units were complete to ensure that the bonding in the model was correct. Crystals of **2b** were weakly diffracting and the data were truncated to the observable diffraction limit.

Despite the crystallographic translation in **2a**, there is a close match between its structure and that of **5**.[S2] Both are in an *antiparallel* conformation, with 6.9 Å between the SQ units in each, and both compounds show end-to-end hydrogen bonding to form indefinitely long SQ arrays. However, because the third SQ of **5** is different to the preceding two, no crystallographic translation is possible for **5**. It has internal H-bonds of 3.004(5) Å (N···O) and 2.868(5) Å (N···O) length along with an 2.927(5) Å long (N···O) intermolecular H-bond. These three values are in good agreement with the average H-bond distance calculated for **2a** (average N···O distance of 2.888(2) Å).

CCDC 2362335, 2362336, 2240191 and 2362337 contain the supplementary crystallographic data for **1a**, **2a**, **2b**, and **3**, respectively. These data can be obtained free of charge via https://www.ccdc.cam.ac.uk/structures/ (or from the Cambridge Crystallographic Data Centre, 12 Union Road, Cambridge CB21EZ, UK; Tel: (+44)1223-336-408; or deposit@ccdc.cam.ac.uk).

Table S3 Crystallographic data

Identification code	**1a**	**2a**	**2b**	**3**
Empirical formula	$C_{18}H_{20}N_2O_3$	$C_{17}H_{19}N_2O_3$	$C_{58}H_{86}N_4O_6$	$C_{88}H_{128}N_6O_9$
Formula weight	312.36	299.34	935.30	1414.01
Temperature/K	234.99(10)	100.01(10)	100.00(10)	100.00(10)
Crystal system	monoclinic	triclinic	triclinic	triclinic
Space group	$P2_1/c$	P-1	P-1	P-1
a/Å	11.3402(3)	6.8442(5)	6.9824(4)	6.9566(4)
b/Å	12.6373(3)	9.2753(7)	12.5511(8)	12.5527(9)
c/Å	11.9364(2)	13.0948(9)	16.1115(12)	16.0926(12)
α/°	90	109.896(7)	83.062(6)	83.262(6)
β/°	95.2625(18)	95.771(6)	79.463(6)	79.447(6)
γ/°	90	104.353(7)	87.661(5)	87.758(6)
Volume/$Å^3$	1703.38(7)	741.50(10)	1377.73(16)	1371.75(17)
Z	4	2	1	0.666667
$\rho_{calc}g/cm^3$	1.218	1.341	1.127	1.141
μ/mm^{-1}	0.678	0.755	0.072	0.572
F(000)	664.0	318.0	510.0	513.0
Crystal size/mm^3	0.133 × 0.114 × 0.041	0.08 × 0.04 × 0.03	0.42 × 0.35 × 0.26	0.106 × 0.037 × 0.006
Radiation	Cu Kα (λ = 1.54184)	Cu Kα (λ = 1.54184)	Mo Kα (λ = 0.71073)	Cu Kα (λ = 1.54184)
2Θ range for data collection/°	7.83 to 151.552	7.332 to 150.902	6.666 to 42.07	5.622 to 151.748
Reflections collected	9522	5680	6330	11685
Independent reflections	3451<br>R_{int} = 0.0254<br>R_{sigma} = 0.0363	2875<br>R_{int} = 0.0352,<br>R_{sigma} = 0.0581]	2958<br>R_{int} = 0.0550<br>R_{sigma} = 0.0794	5363<br>R_{int} = 0.0645<br>R_{sigma} = 0.0975
Data/restraints/parameters	3451/0/215	2875/0/212	2958/0/316	5363/219/310
Goodness-of-fit on F^2	1.081	1.070	1.030	1.043
Final R indexes [I>=2σ (I)]	R_1 = 0.0392,<br>wR_2 = 0.1061	R_1 = 0.0503,<br>wR_2 = 0.1323	R_1 = 0.0560<br>wR_2 = 0.1364	R_1 = 0.0643<br>wR_2 = 0.1638
Final R indexes [all data]	R_1 = 0.0472<br>wR_2 = 0.1109	R_1 = 0.0643<br>wR_2 = 0.1404	R_1 = 0.0761<br>wR_2 = 0.1513	R_1 = 0.1063<br>wR_2 = 0.1886
Largest diff. peak/hole / e $Å^{-3}$	0.20/-0.17	0.27/-0.23	0.24/-0.23	0.31/-0.25

5.2. Crystal structure representations

a.

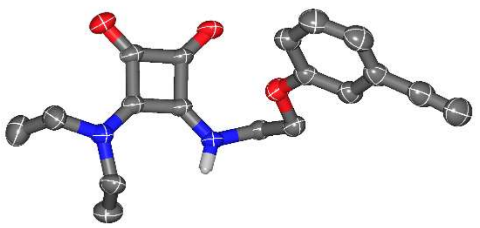

b

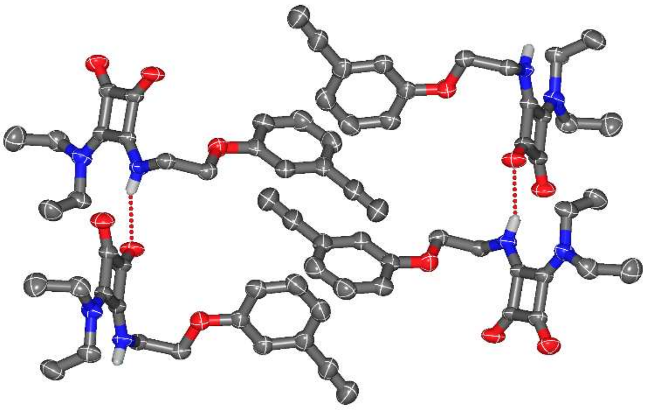

c.

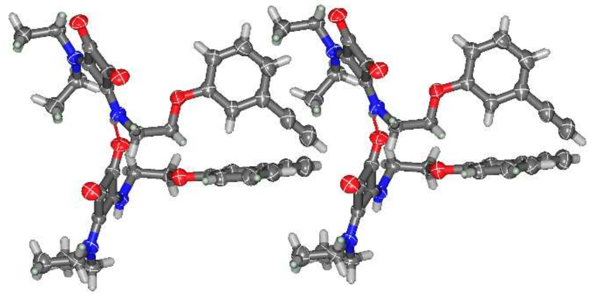

Figure S40. a. ORTEP representation of the asymmetric unit (a) and a pair of head-to-tail dimers (b) of compound **1a** (thermal ellipsoids set at 50% probability). c. Representation of the CH-π interactions between SQ units of different head-to-tail chains of **1a**.

a.

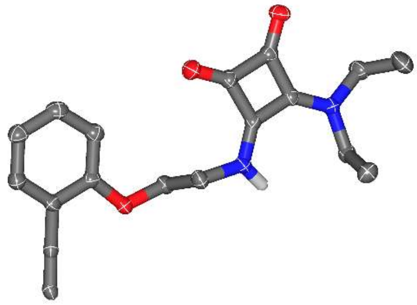

b.

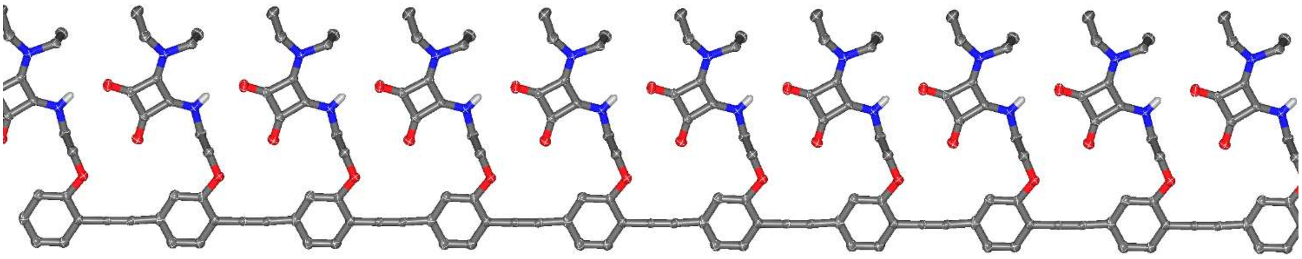

c.

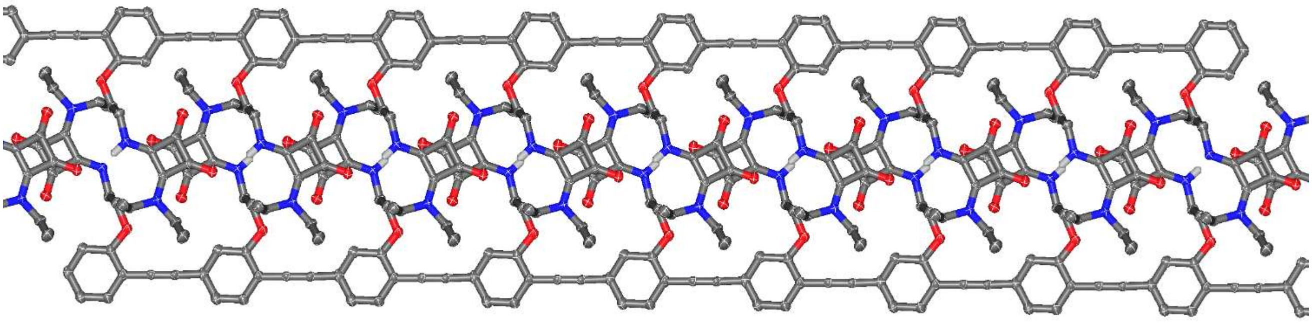

Figure S41. a. ORTEP representation of the asymmetric unit of compound **2a** (thermal ellipsoids set at 50% probability). b. Representation of **2a** to show the translocation of **2a** by half a molecule (50% occupancy) in indefinitely extended head-to-tail chains. c. Representation of the packing of **2a** as head-to-tail chains with opposing macrodipoles.

a.

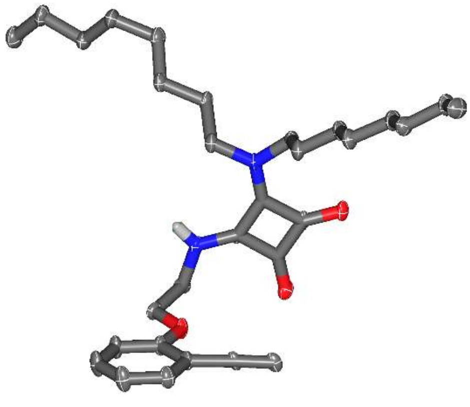

b.

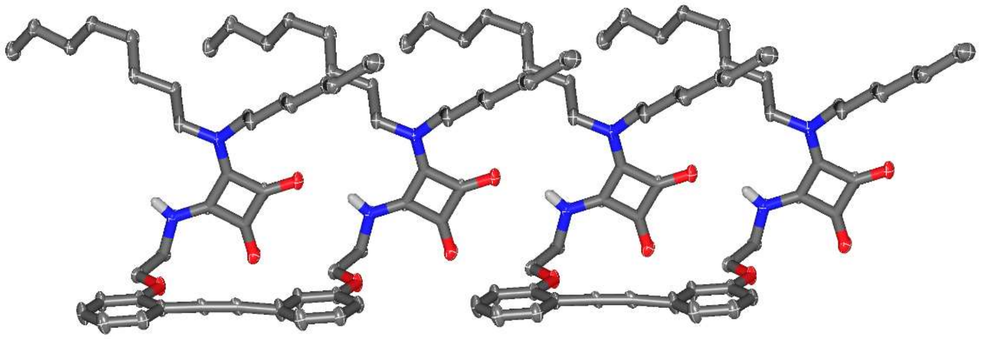

c.

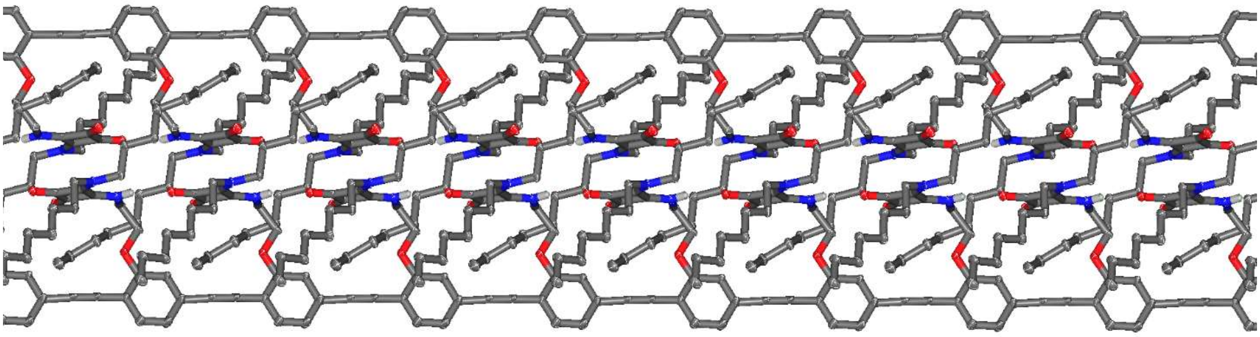

Figure S42. a. ORTEP representation of the asymmetric unit of compound **2b** (thermal ellipsoids set at 50% probability). b. Representation of a section of the packing of **2b** showing a head-to-tail dimer unit. c. Representation of the packing of **2b** as indefinitely extended head-to-tail chains with opposing macrodipoles.

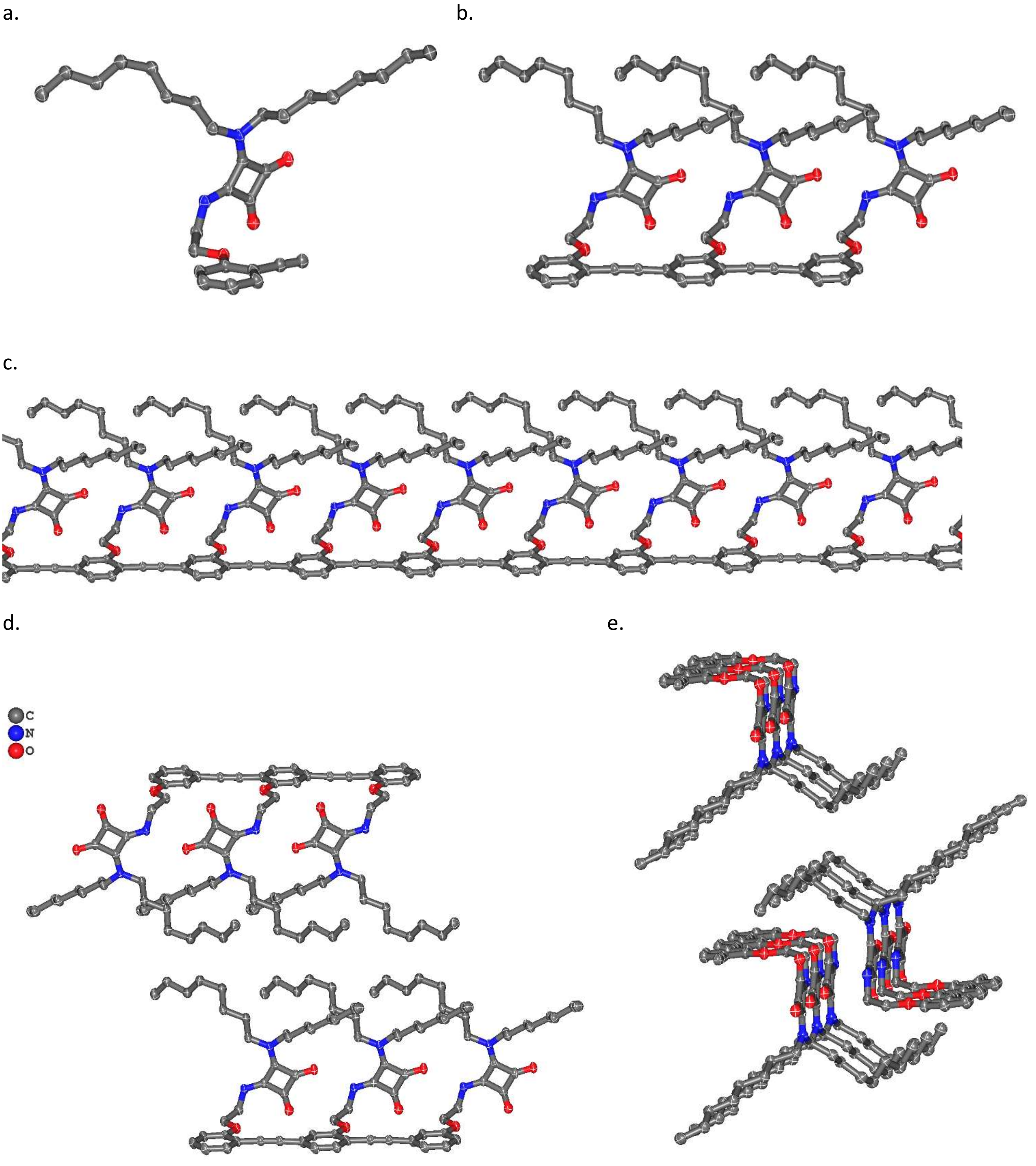

Figure S43. a. ORTEP representation of the asymmetric unit of compound **3** (thermal ellipsoids set at 50% probability). b. Representation of a section of the packing of **3** showing a single molecule (lateral translocation by a third gives 33% occupancy for each atom in a trimer). c. Representation of the packing of **3** as indefinitely extended head-to-tail chains. d-e. Representation of the packing of **3** as head-to-tail chains with opposing macrodipoles.

a.

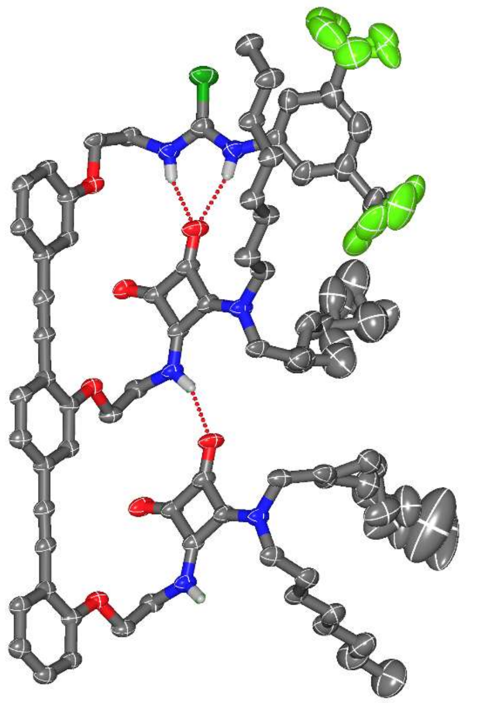

b.

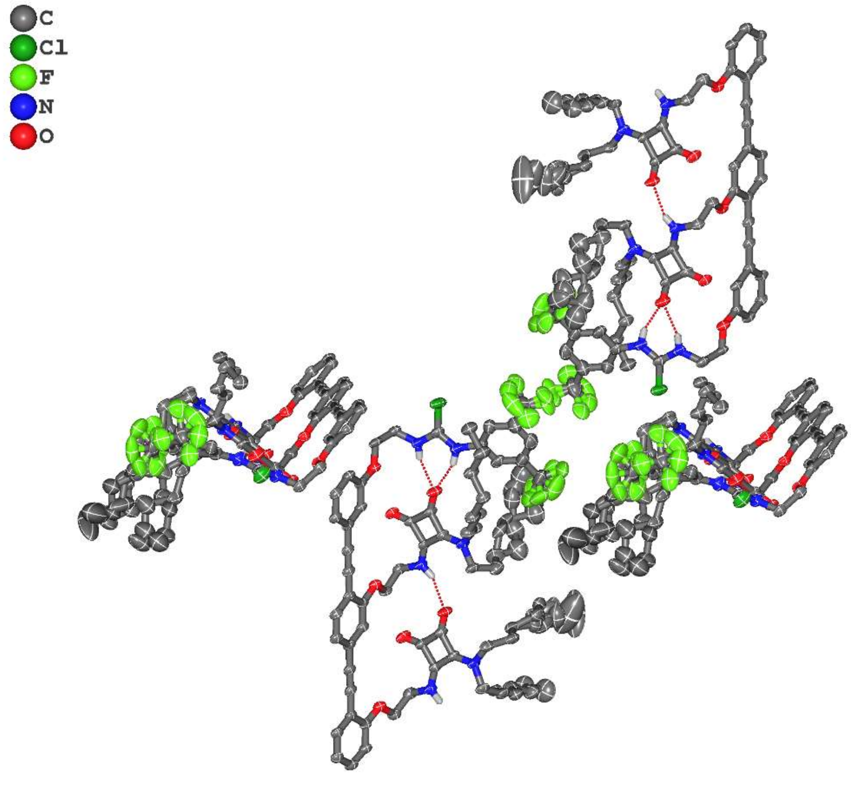

Figure S44. a. ORTEP representation of the asymmetric unit of compound **4** (thermal ellipsoids set at 50% probability). b. Representation of the packing of **4** in a zigzag manner.

a. b. c.

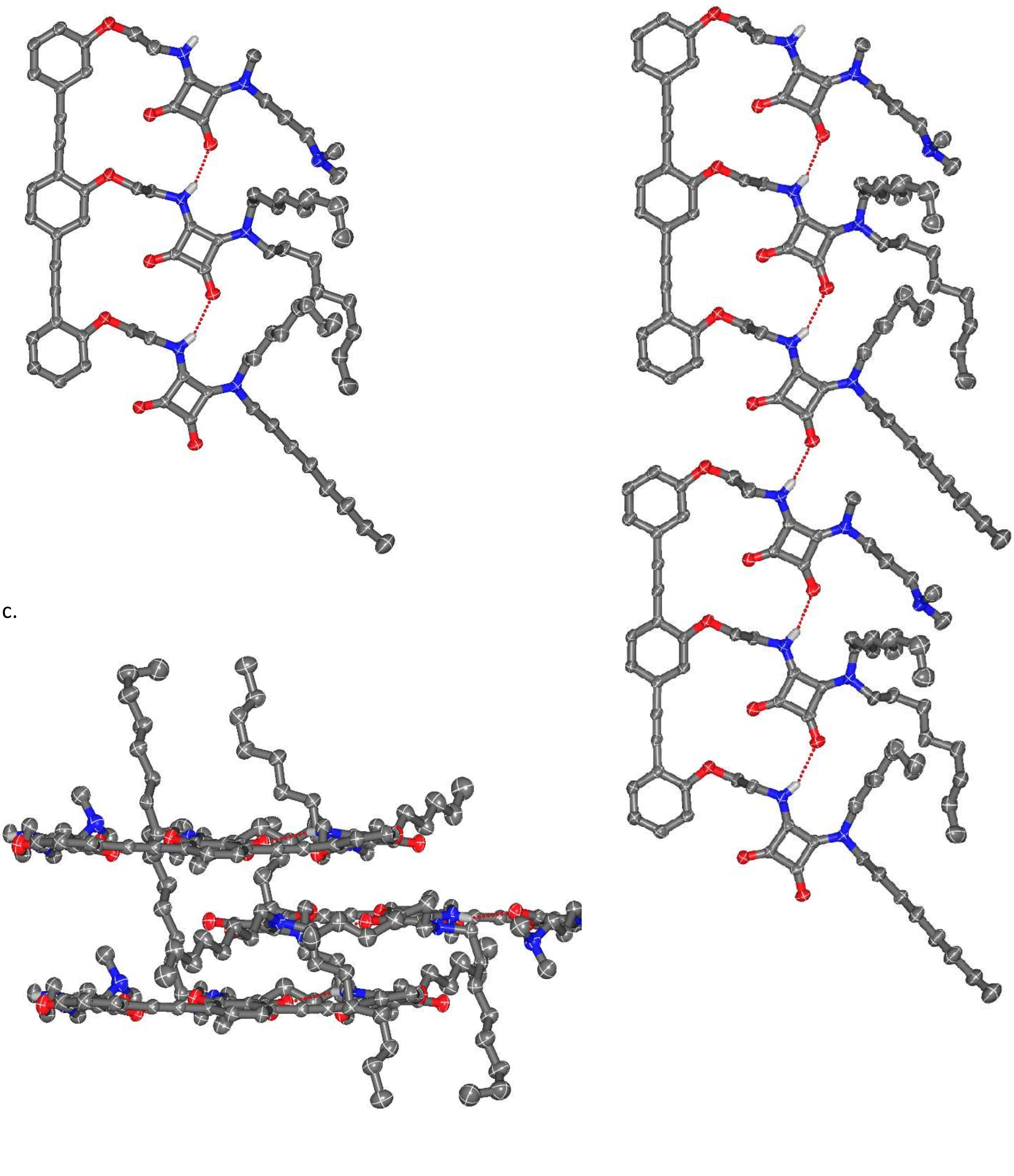

Figure S45. a. ORTEP representation of the asymmetric unit of compound **5** (thermal ellipsoids set at 50% probability). b-c. Representation of the packing of **5** as head-to-tail chains.

6. NMR spectra (complete)

3-(Diethylamino)-4-((2-(3-ethynylphenoxy)ethyl)amino)cyclobut-3-ene-1,2-dione (1a)

^{1}H NMR:

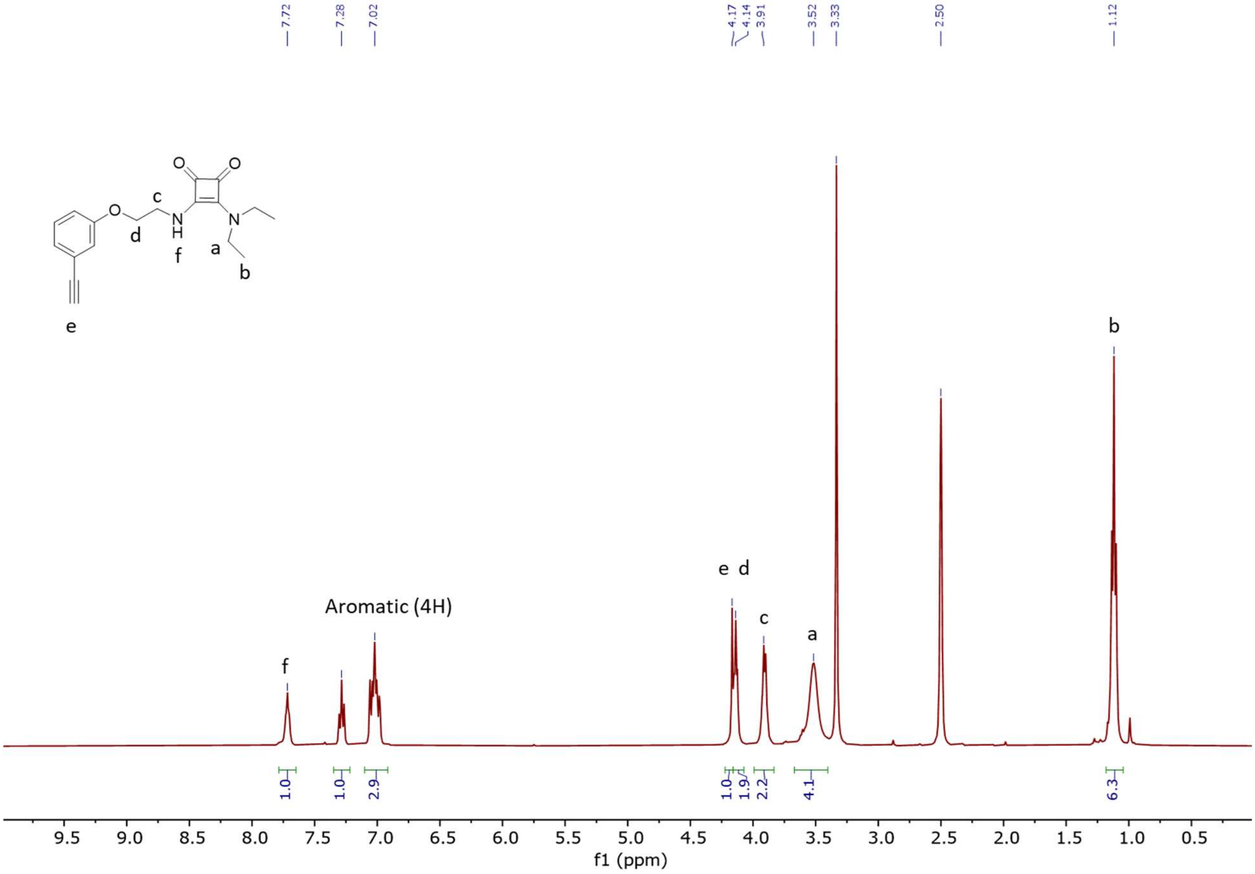

^{13}C NMR:

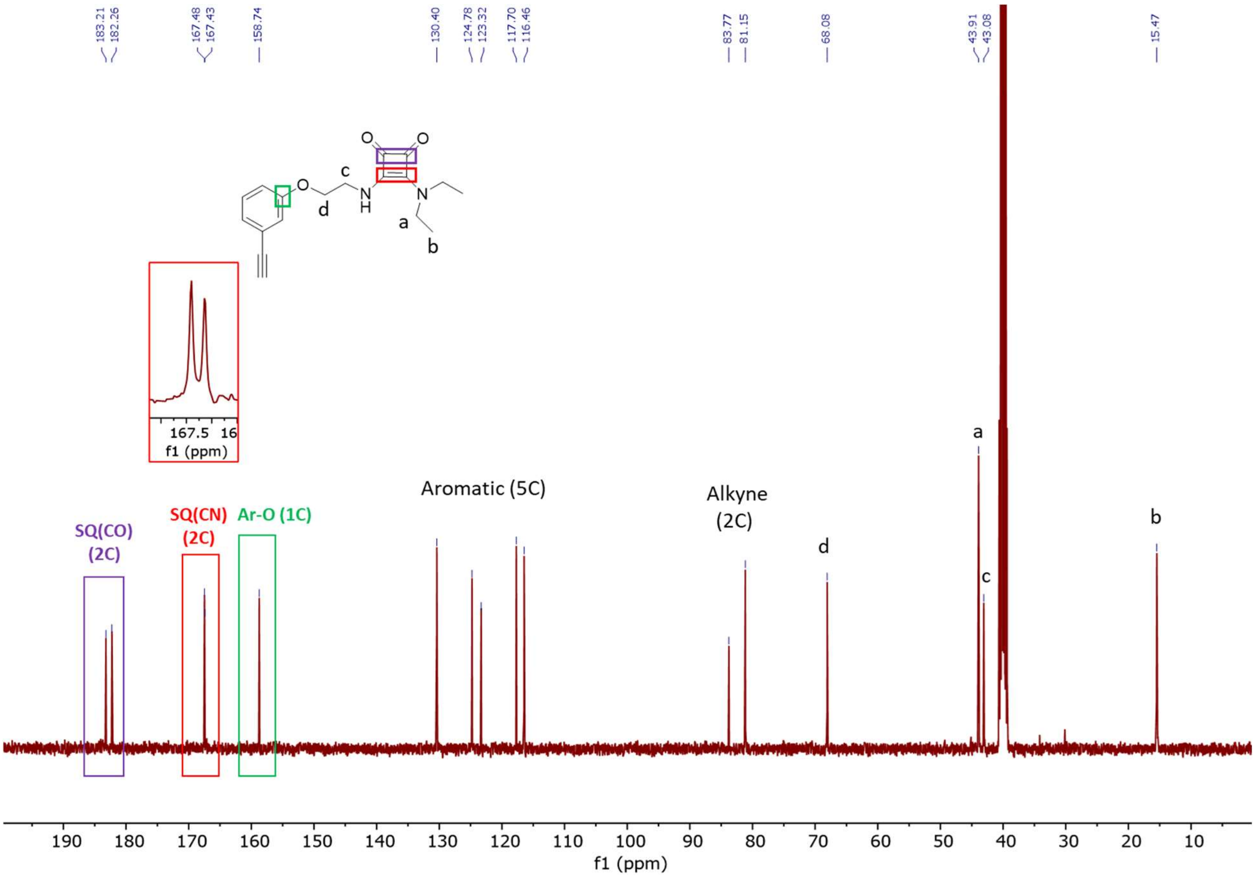

2D NMR:

(HSQC)

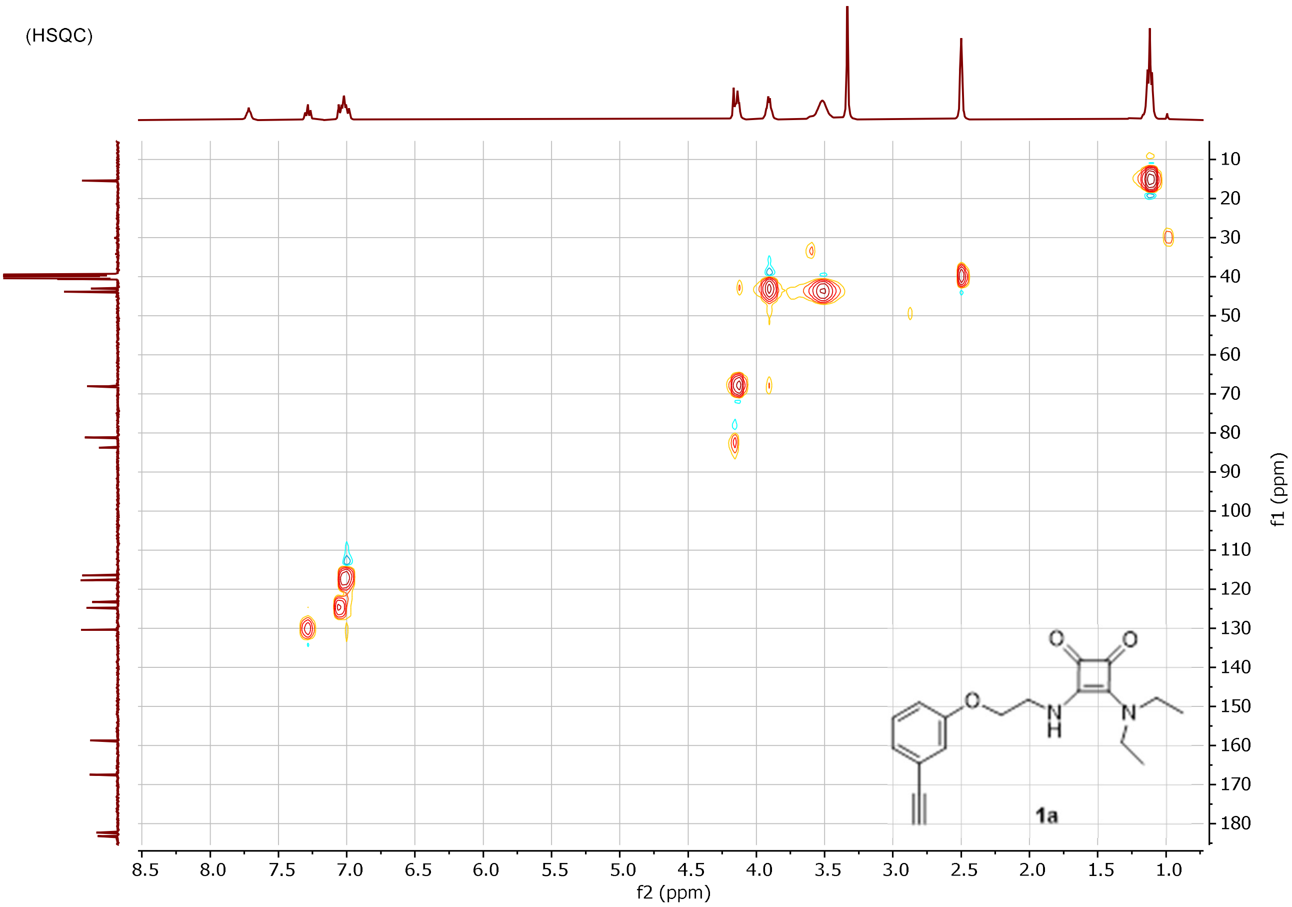

(HMBC)

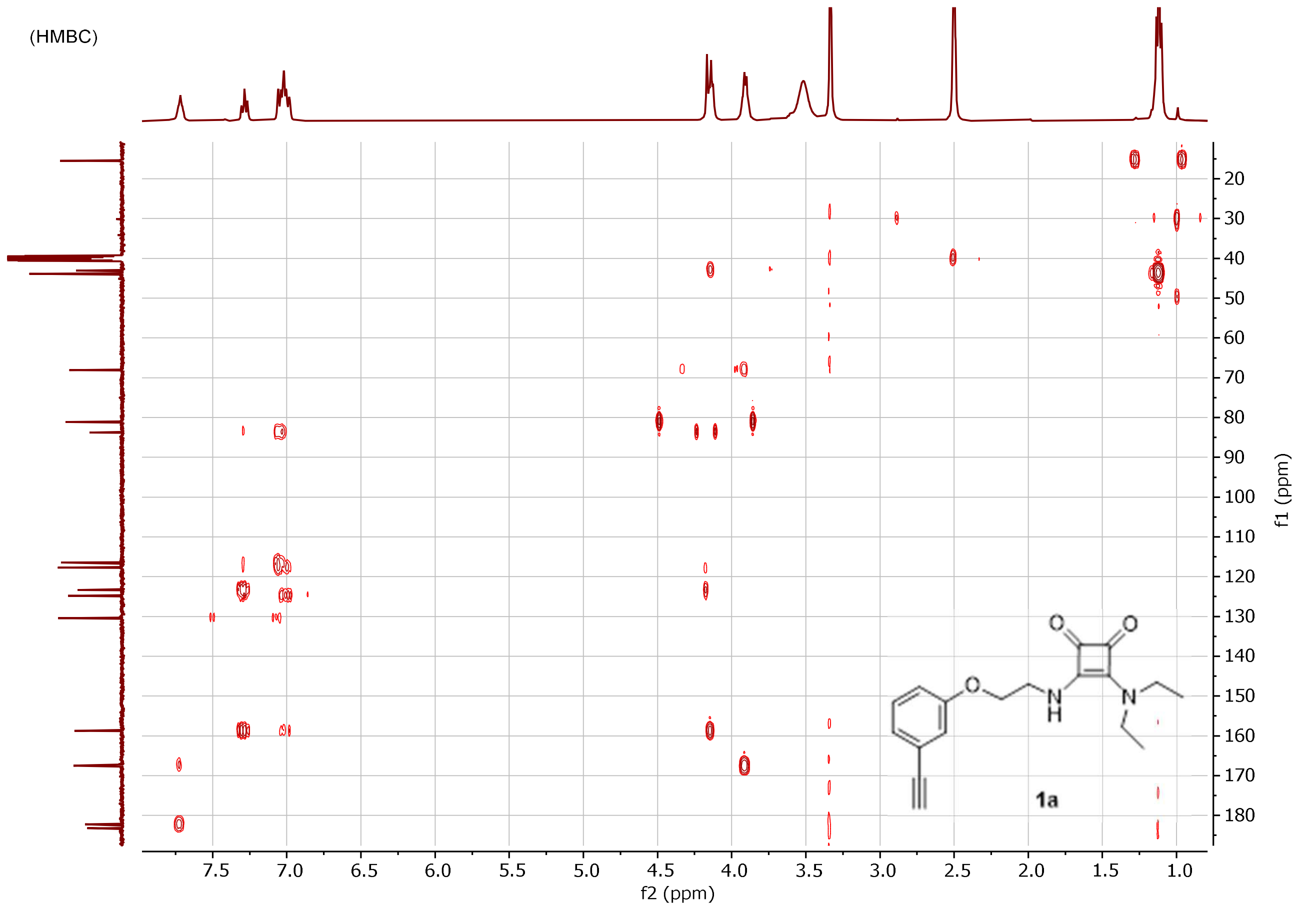

7.5
7.0
6.5
6.0
5.5
5.0
4.5
4.0
3.5
3.0
2.5
2.0
1.5
1.0
f2 (ppm)
20
30
40
50
60
70
80
90
100
110
120
130
140
150
160
170
180
f1 (ppm)
O
O
O
N
H
N
1a

3-(Dioctylamino)-4-((2-(3-ethynylphenoxy)ethyl)amino)cyclobut-3-ene-1,2-dione (1b)
^{1}H NMR:

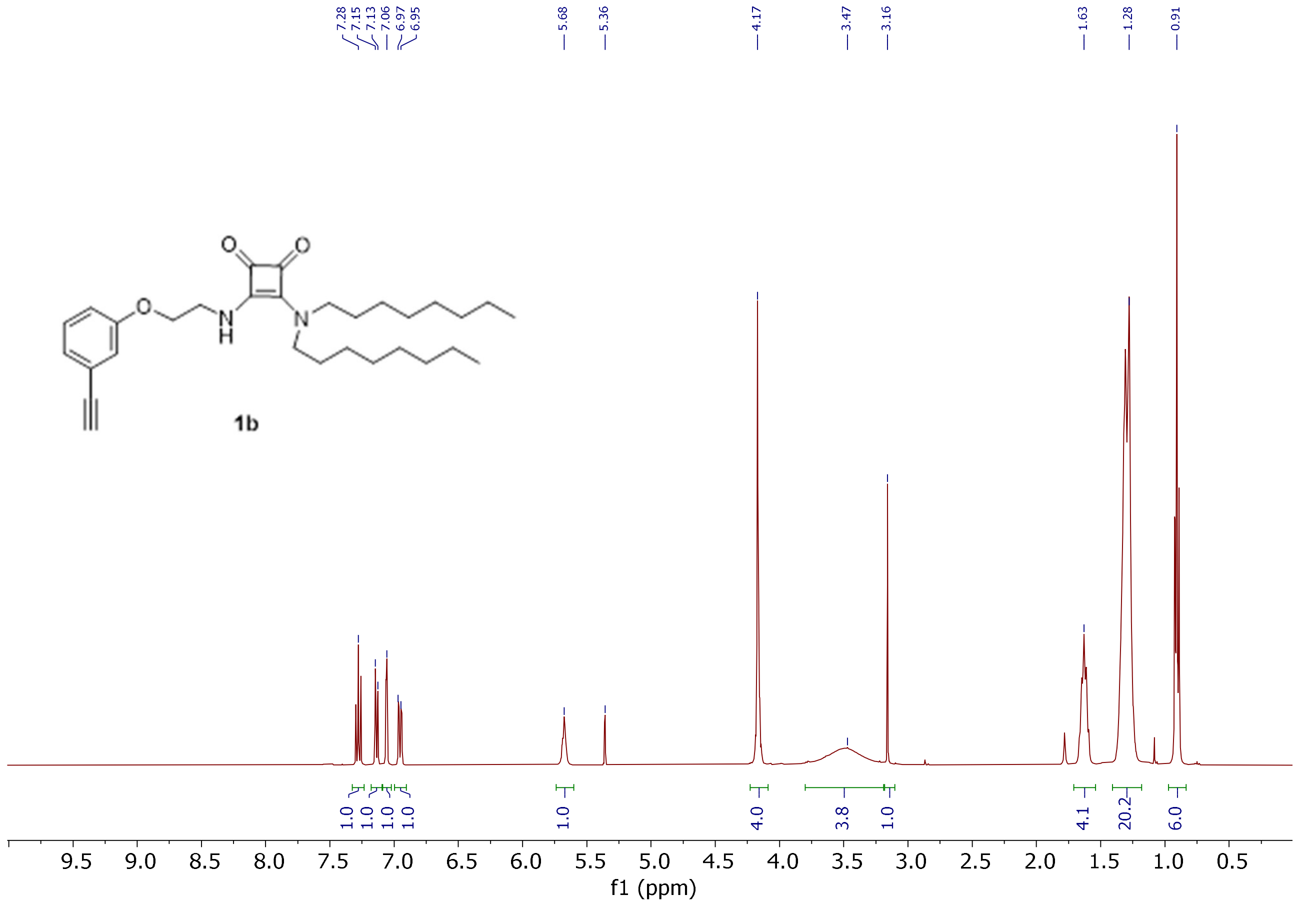

^{13}C NMR:

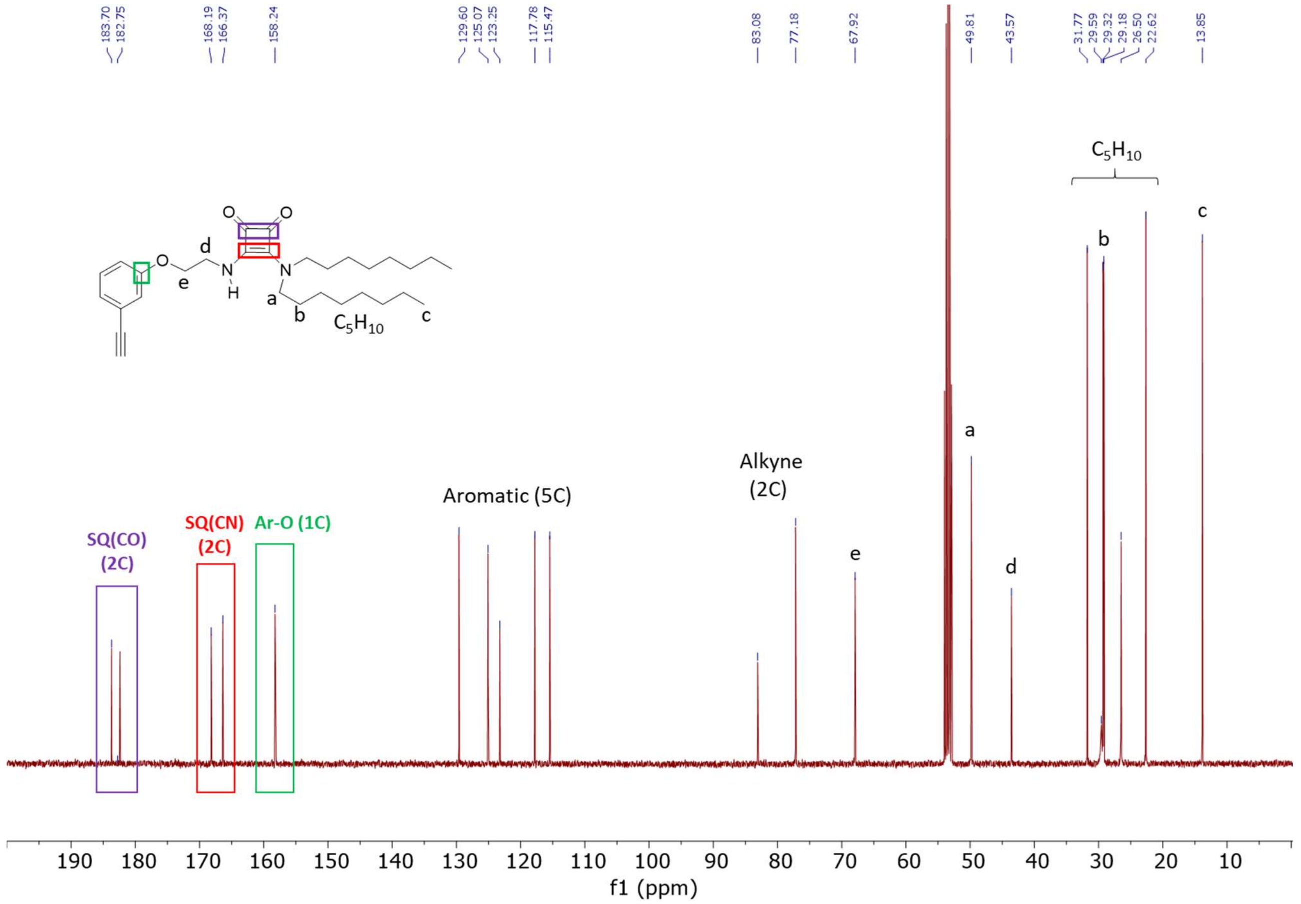

2D NMR:

(COSY)

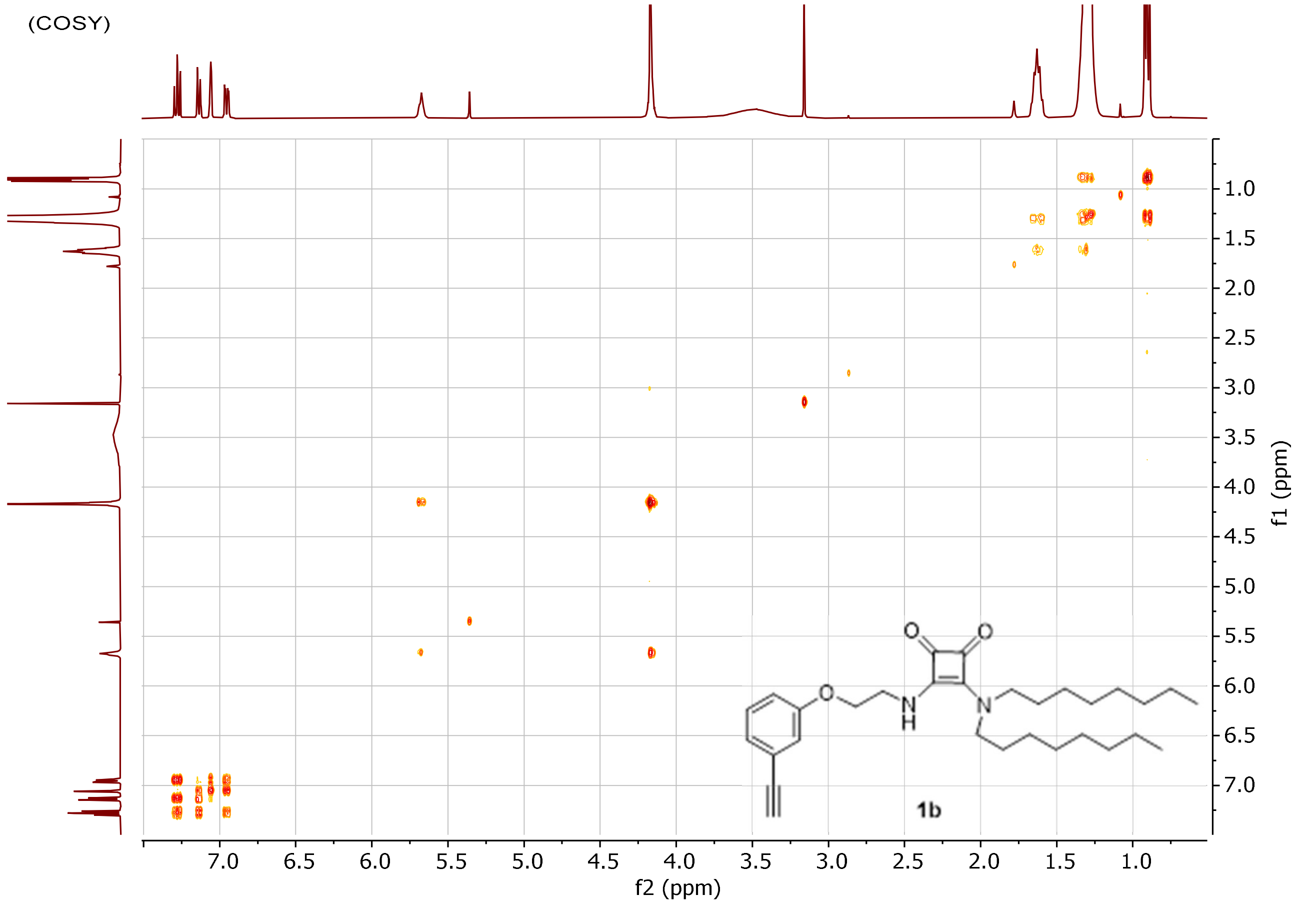

(HSQC)

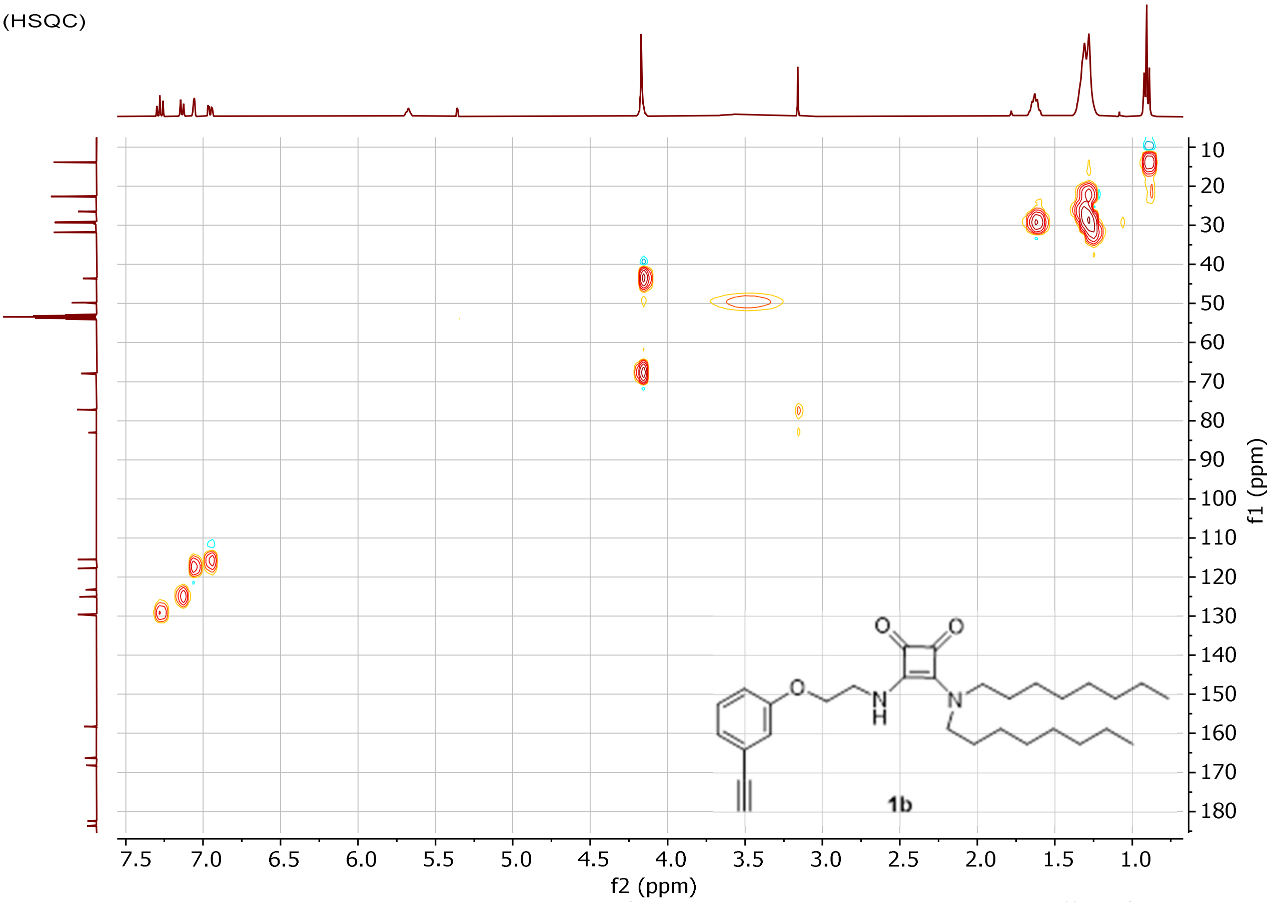

(HMBC)

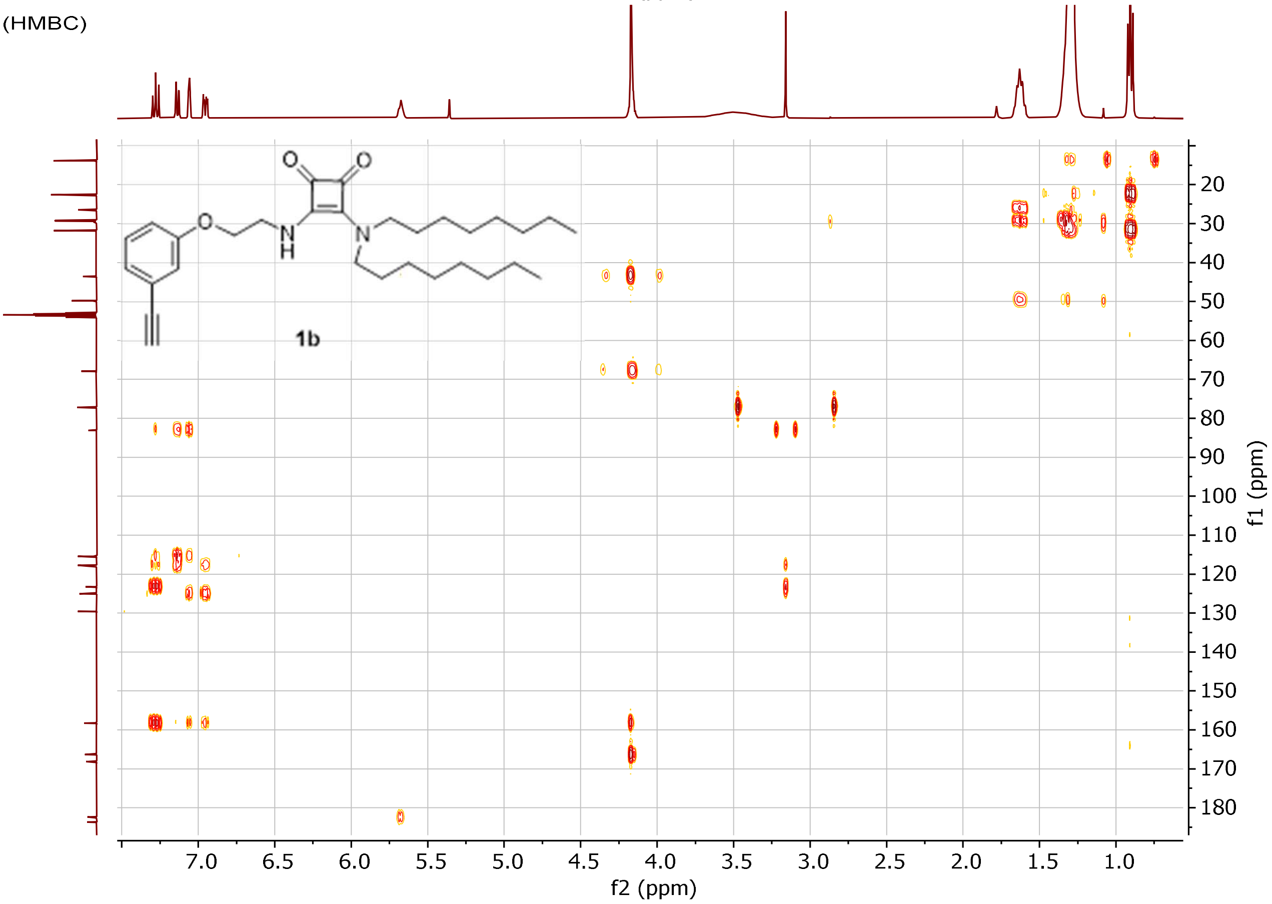

3-(Diethylamino)-4-((2-(2-((3-(2-((2-(diethylamino)-3,4-dioxocyclobut-1-en-1-yl)amino)ethoxy)phenyl)-ethynyl)phenoxy)ethyl)amino)cyclobut-3-ene-1,2-dione (2a)

^{1}H NMR:

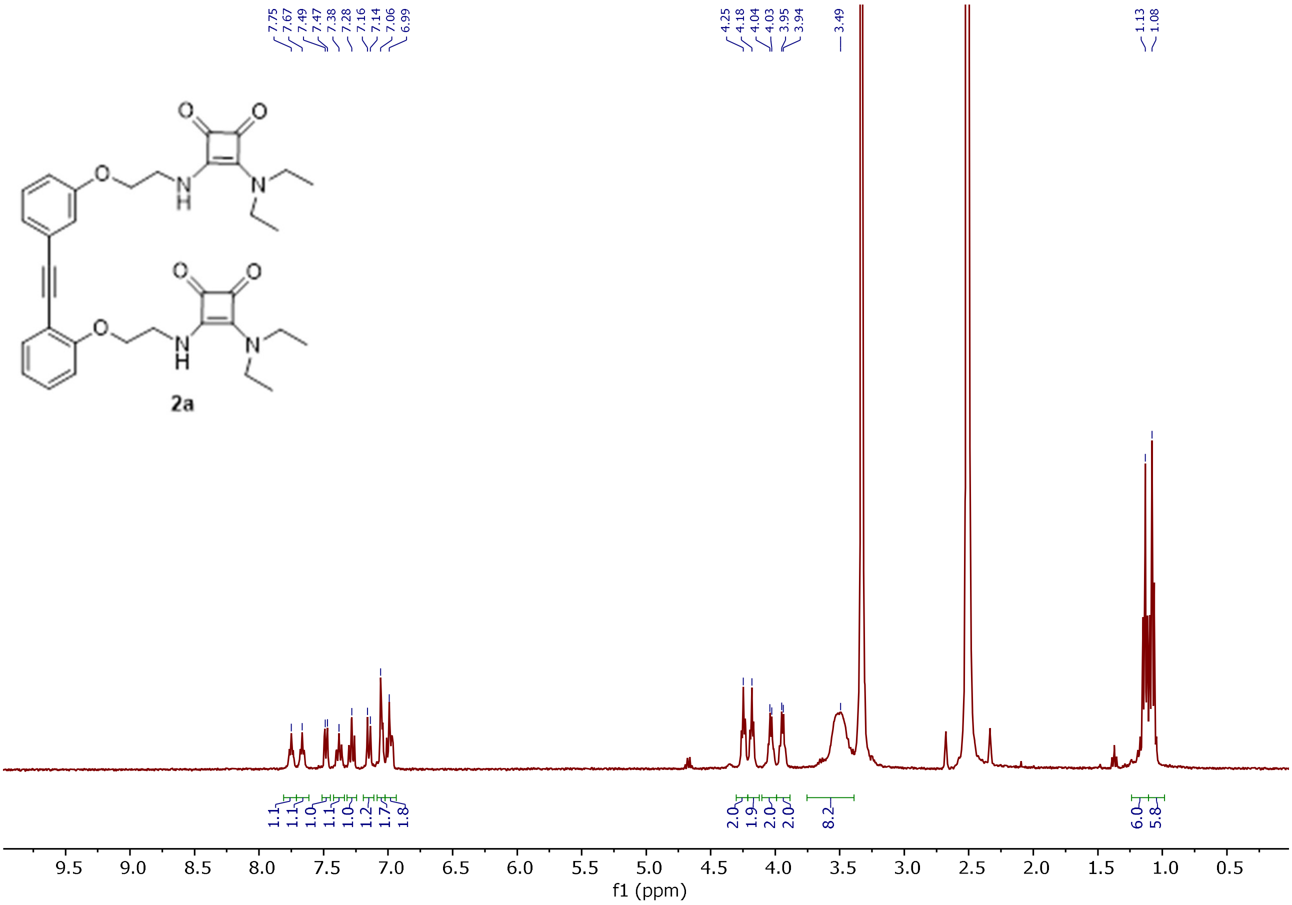

3-(Dioctylamino)-4-((2-(2-((3-(2-((2-(dioctylamino)-3,4-dioxocyclobut-1-en-1-yl)amino)ethoxy)phenyl)-ethynyl)phenoxy)ethyl)amino)cyclobut-3-ene-1,2-dione (2b)

^{1}H NMR:

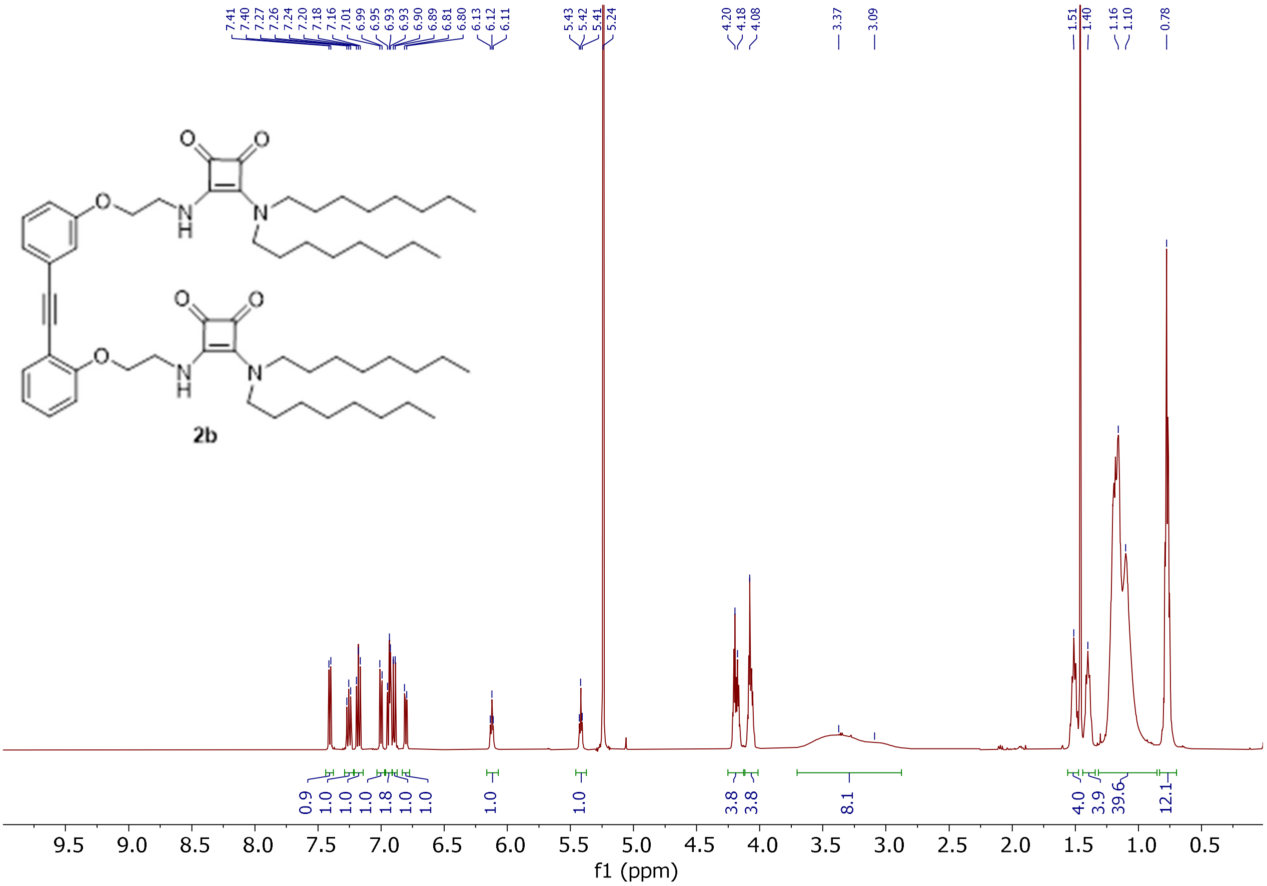

^{13}C NMR:

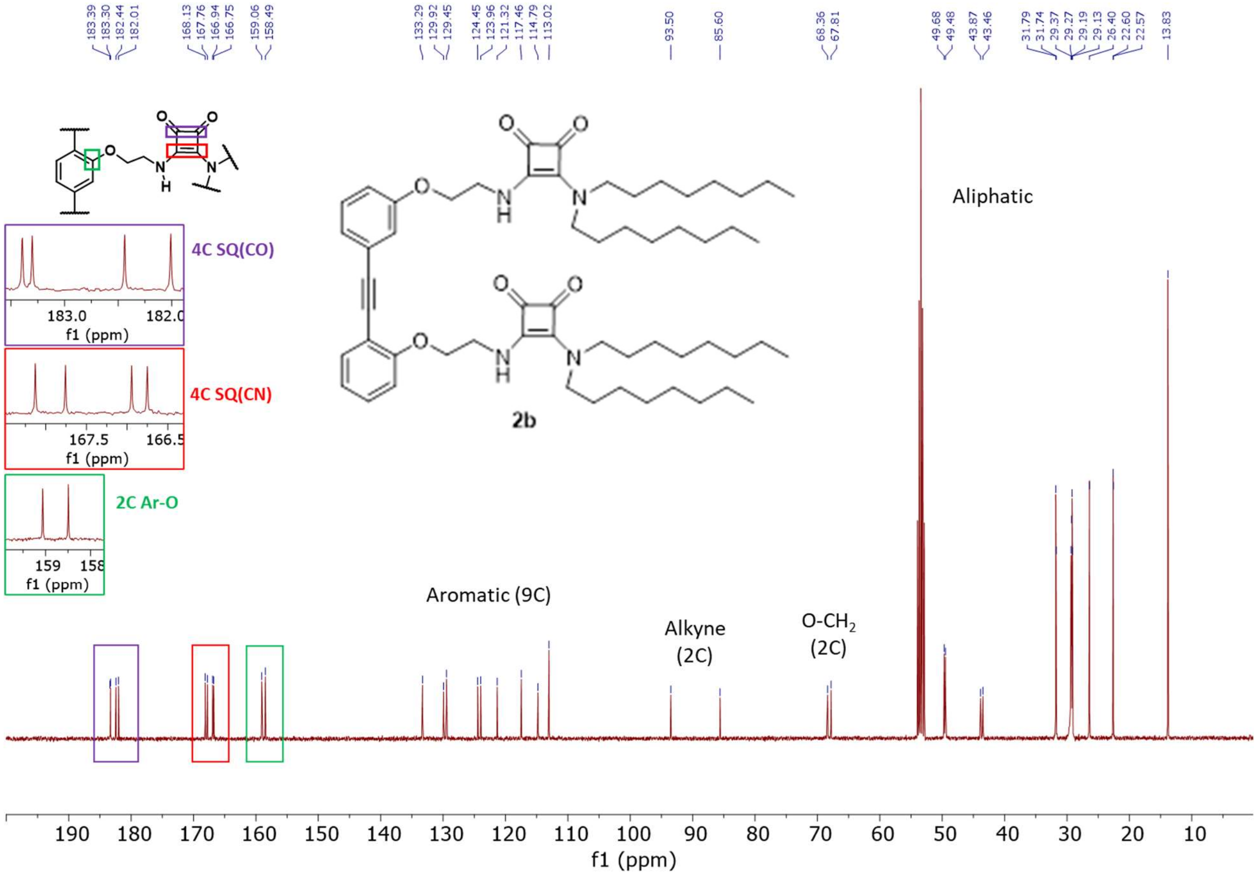

2D NMR:

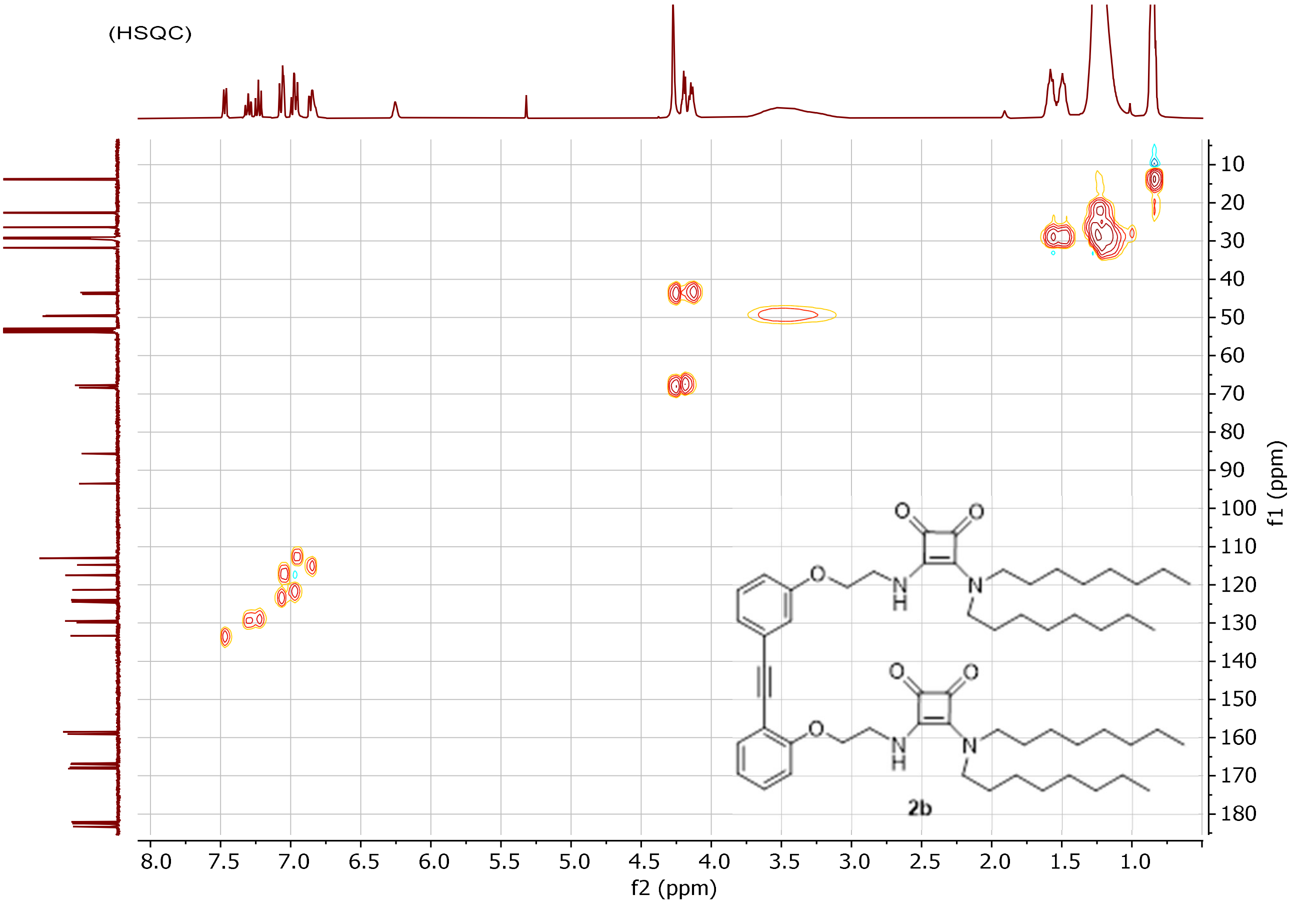

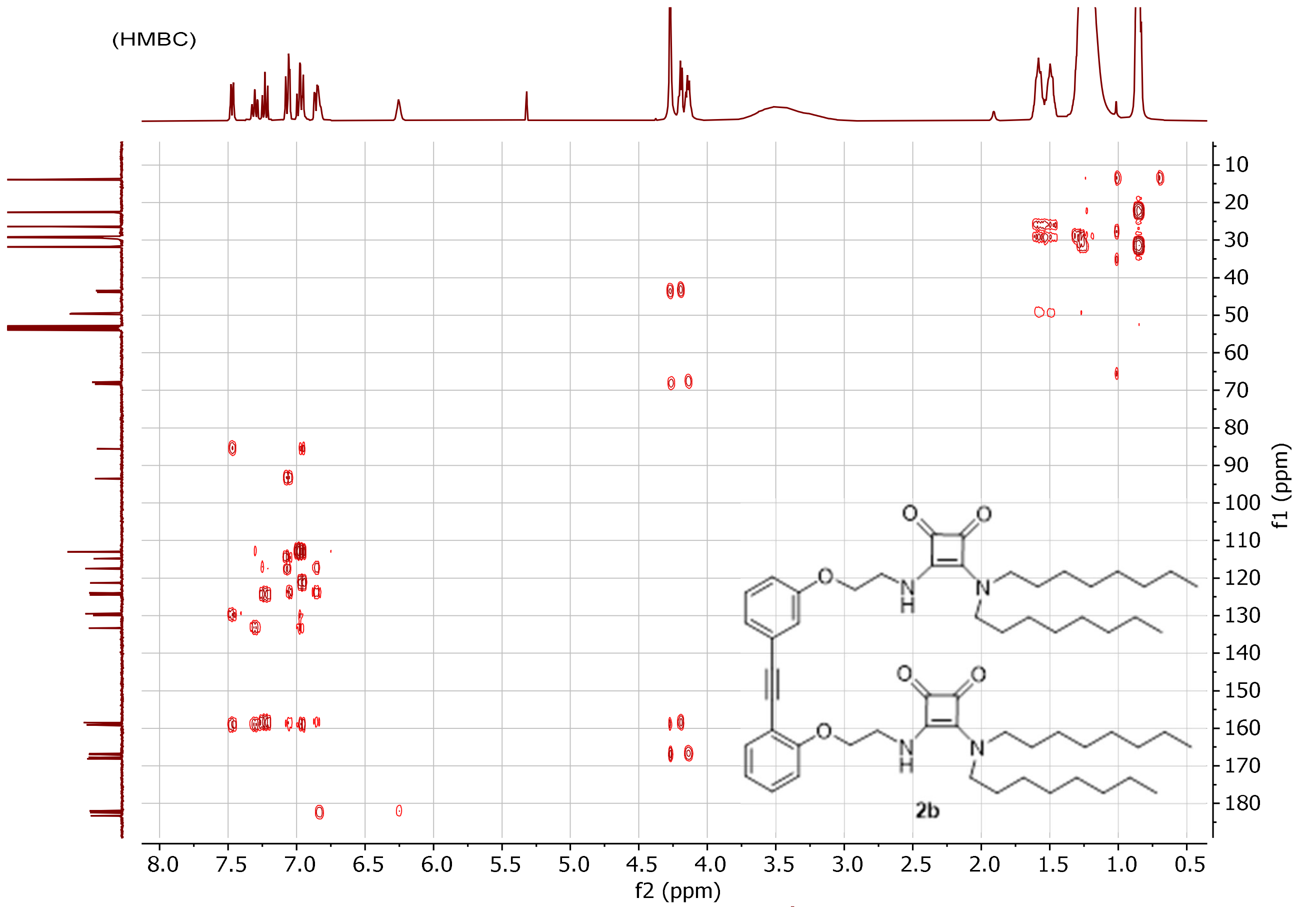
(HMBC)
f1 (ppm)
f2 (ppm)
2b

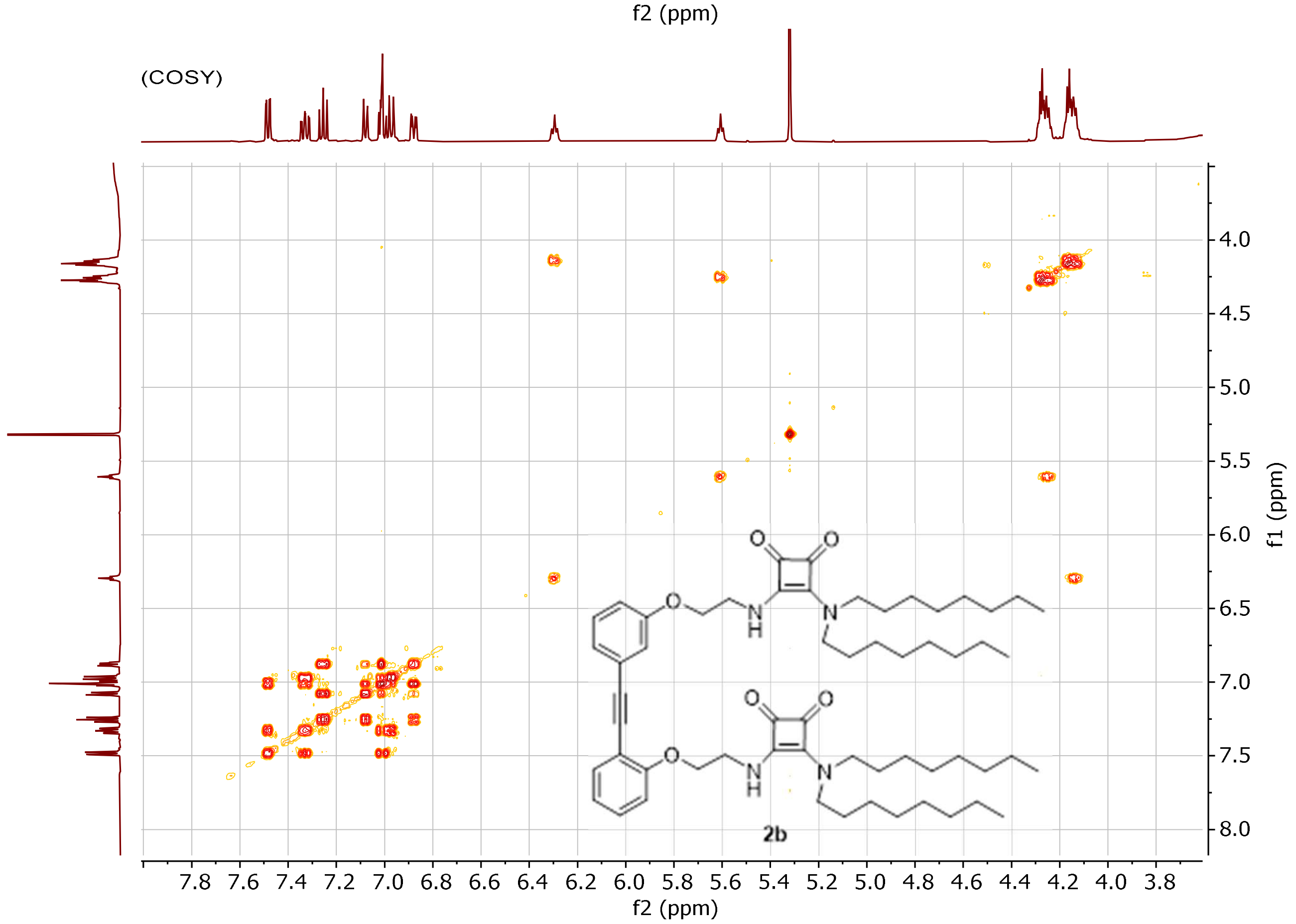
(COSY)
f1 (ppm)
f2 (ppm)
2b

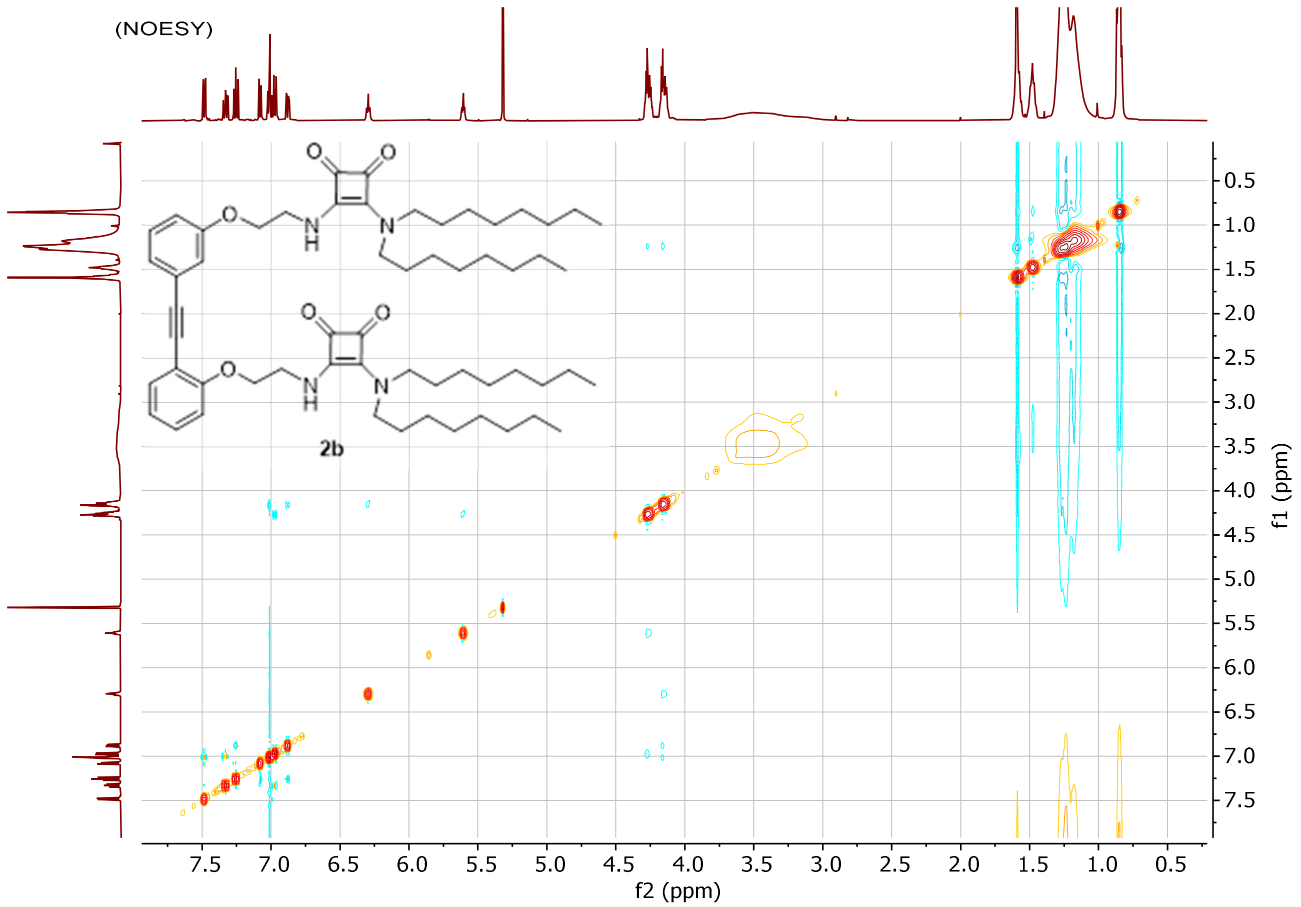
(NOESY)
O
N
H
N
2b
f2 (ppm)
f1 (ppm)

***tert*-Butyl (2-(2-((3-(2-((tert-butoxycarbonyl)amino)ethoxy)-4-((3-(2-((tert-butoxycarbonyl)amino)ethoxy)-phenyl)ethynyl)phenyl)ethynyl)phenoxy)ethyl)carbamate (12)**

^{1}H NMR:

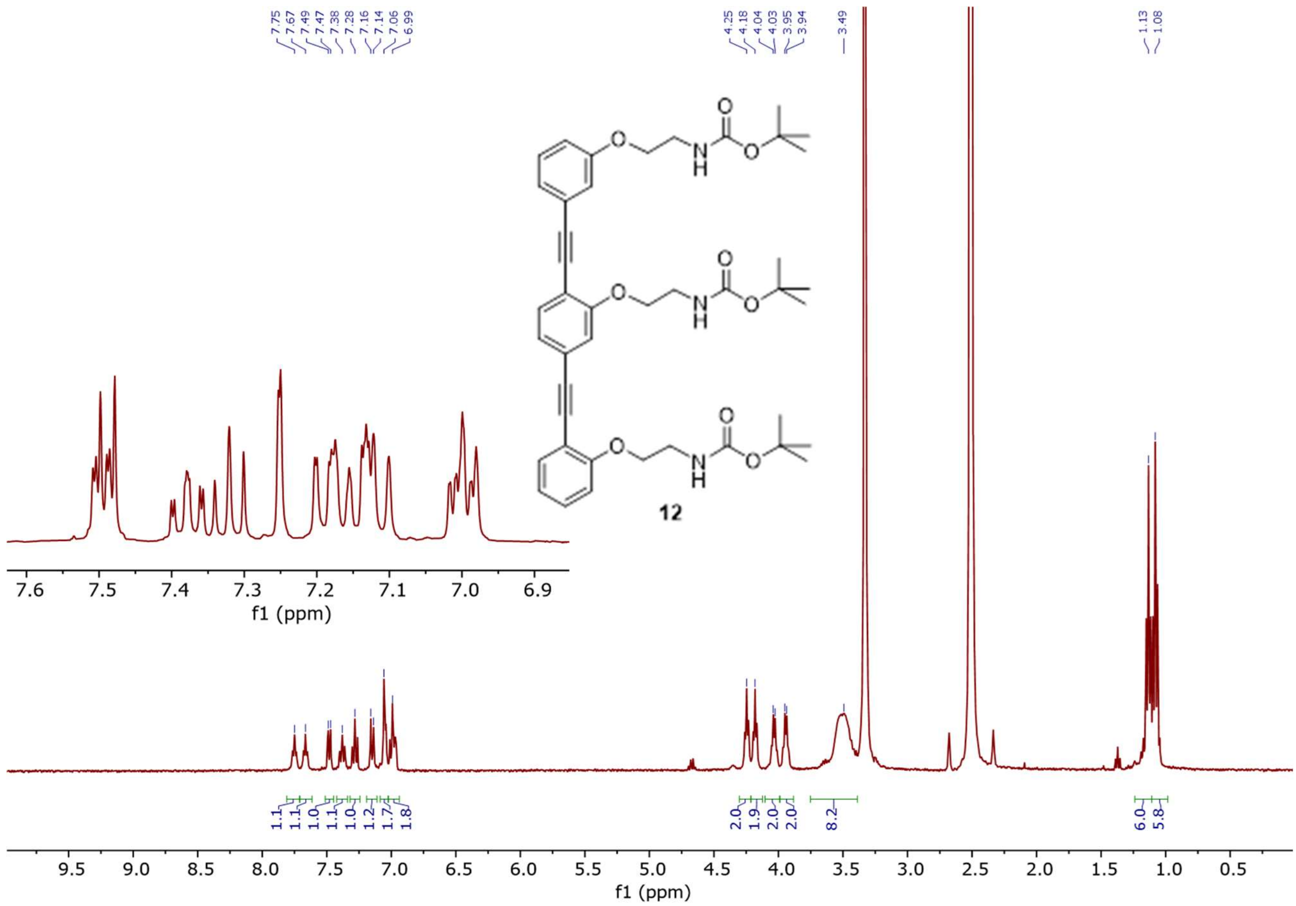

2D NMR:

(COSY)

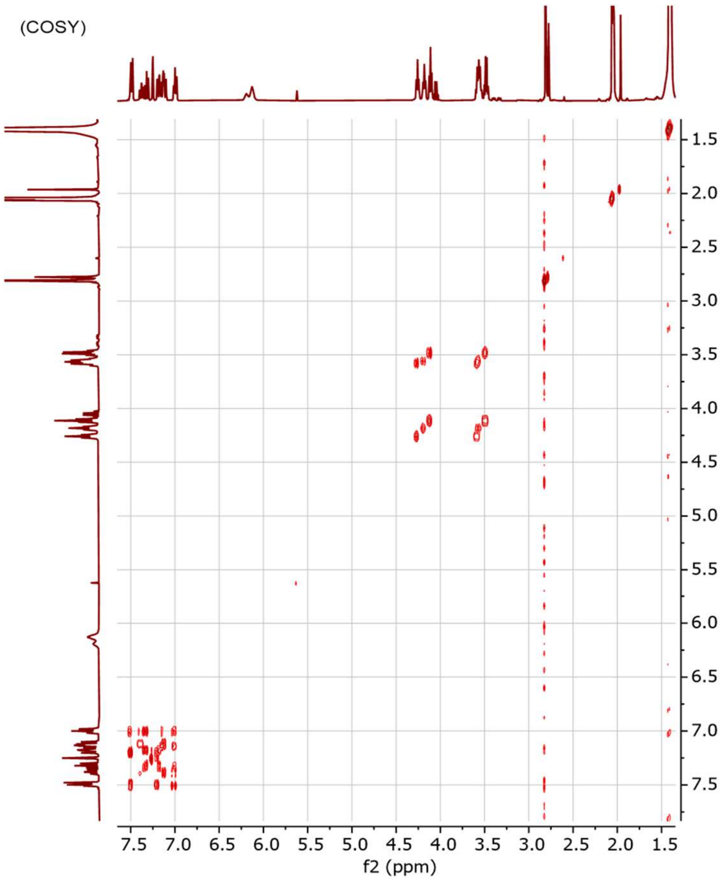

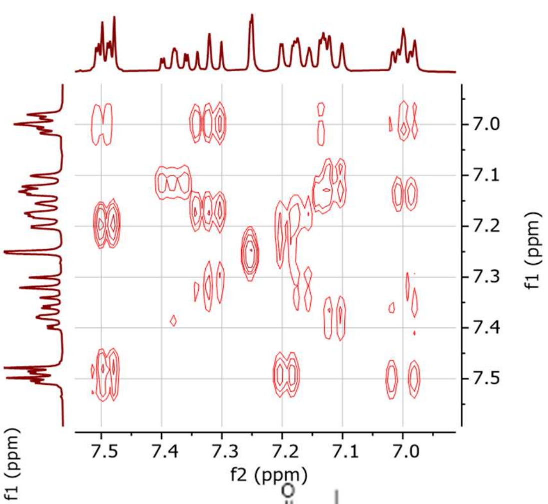

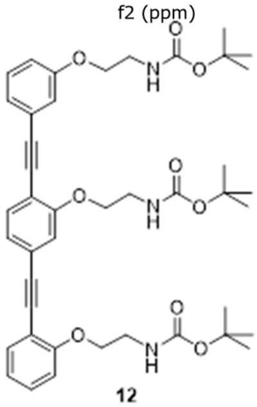

12

3-(Dioctylamino)-4-((2-(3-((2-(2-((2-(dioctylamino)-3,4-dioxocyclobut-1-en-1-yl)amino)ethoxy)-4-((2-(2-((2-(dioctylamino)-3,4-dioxocyclobut-1-en-1-yl)amino)ethoxy)phenyl)ethynyl)phenyl)ethynyl)phenoxy)-ethyl)amino)cyclobut-3-ene-1,2-dione (3)

^{1}H NMR:

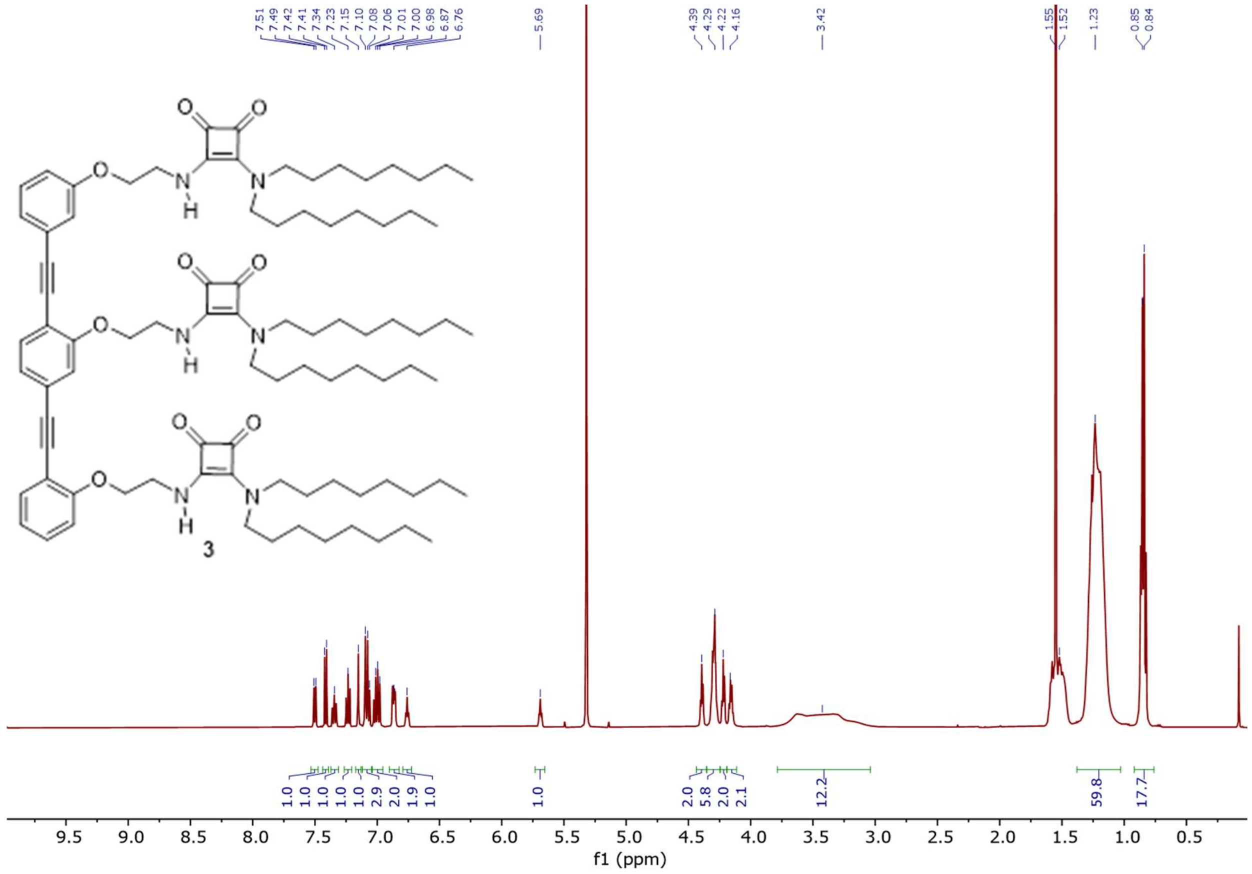

^{13}C NMR:

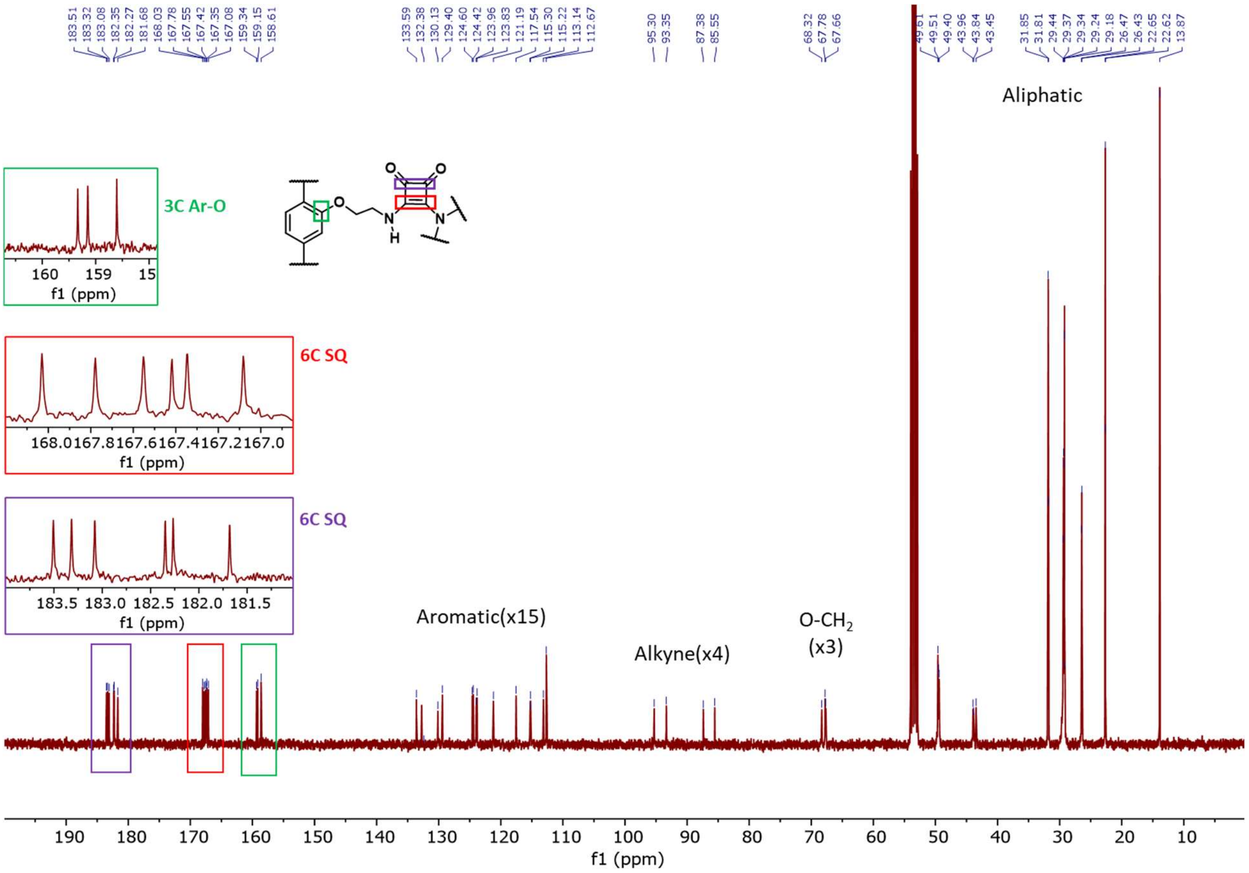

2D NMR:

(HSQC)

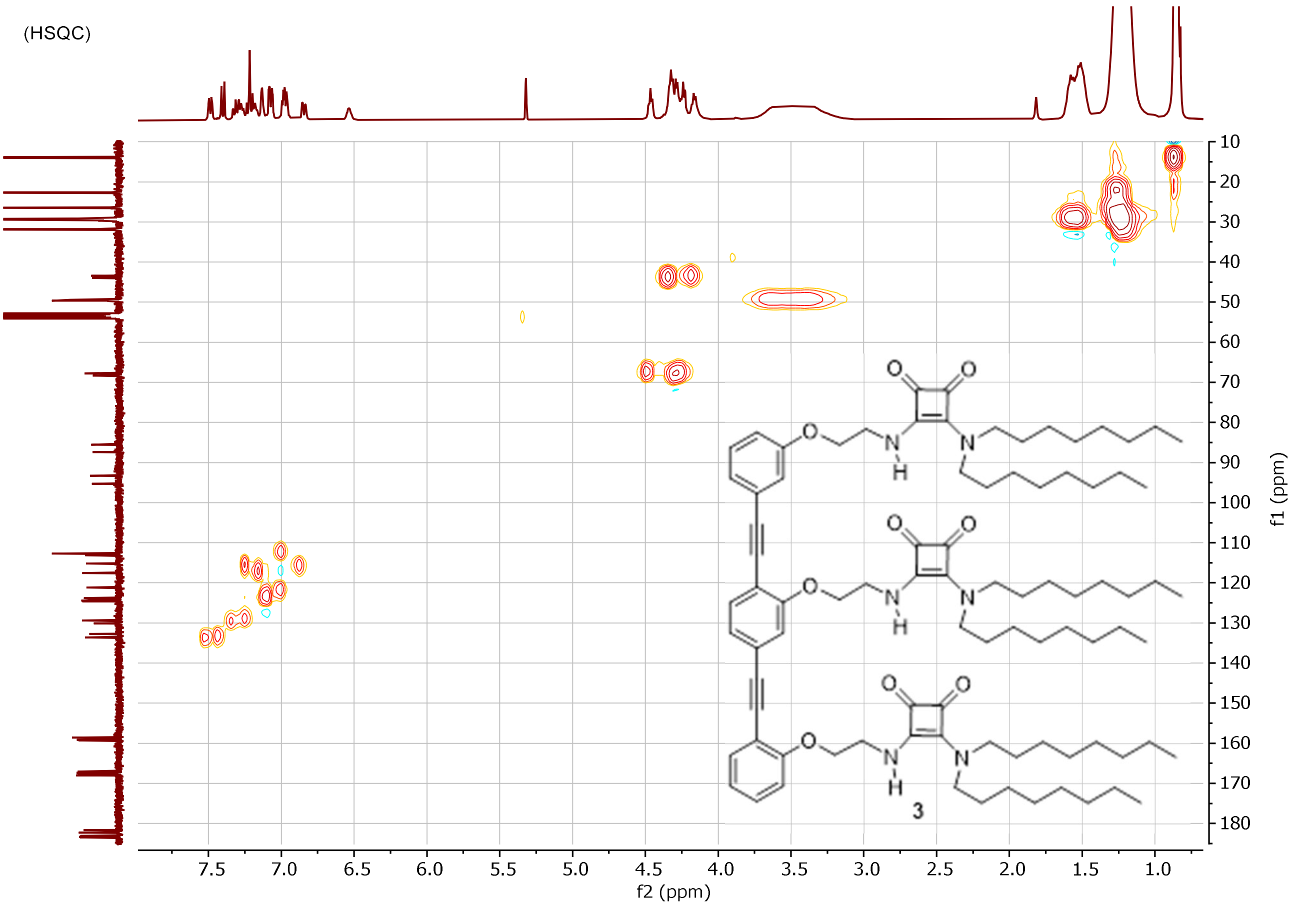

(HMBC)

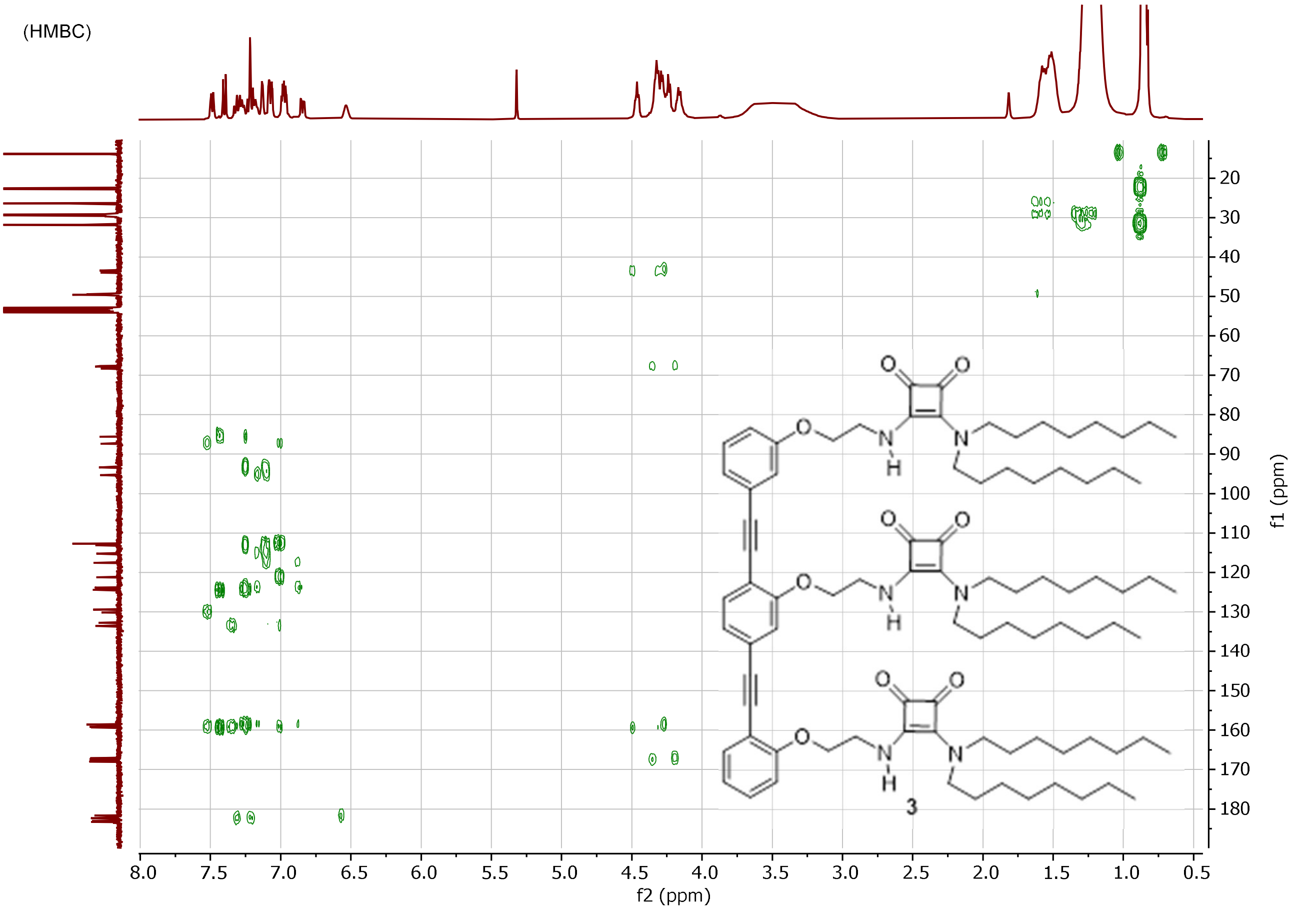

(COSY)

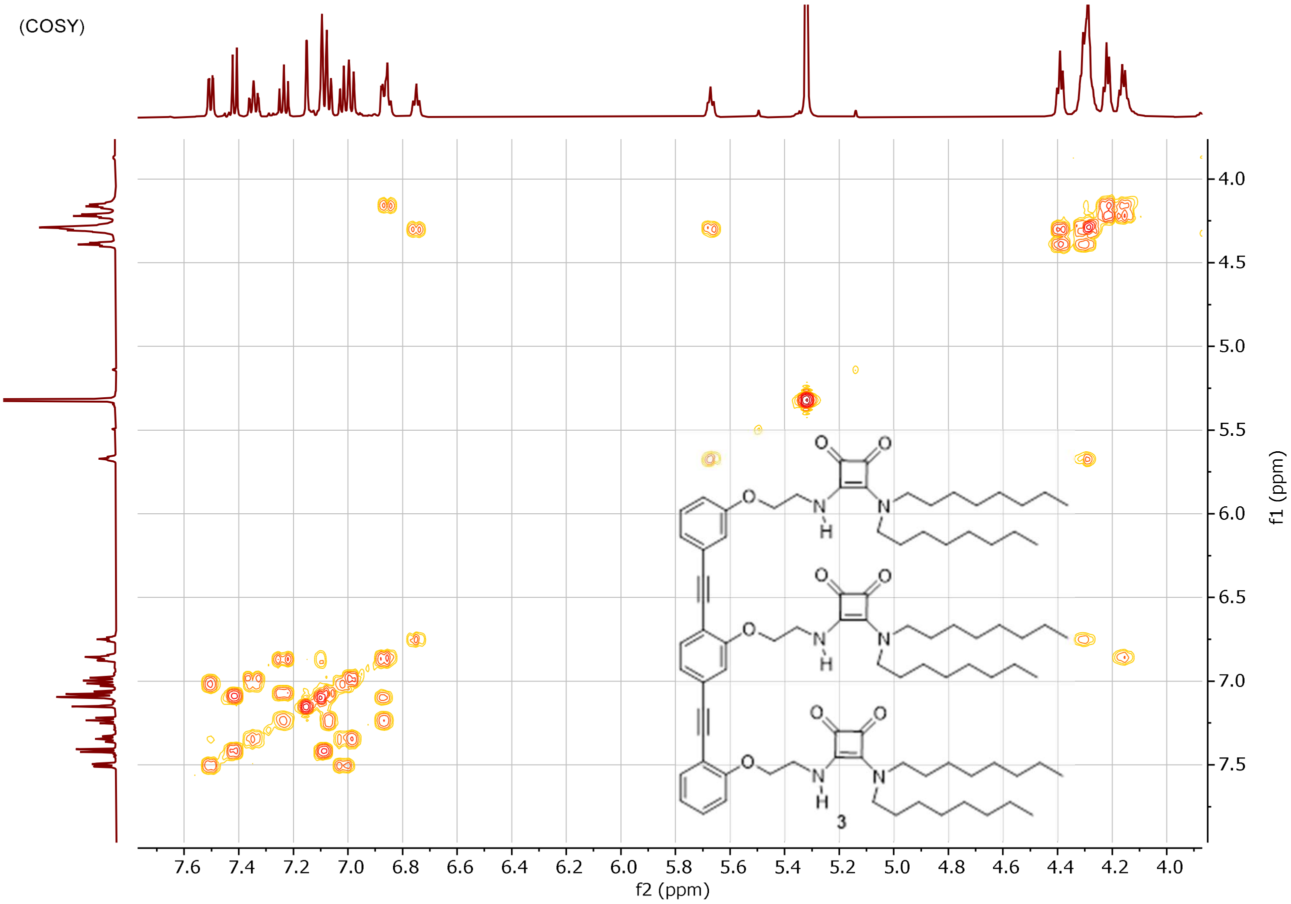

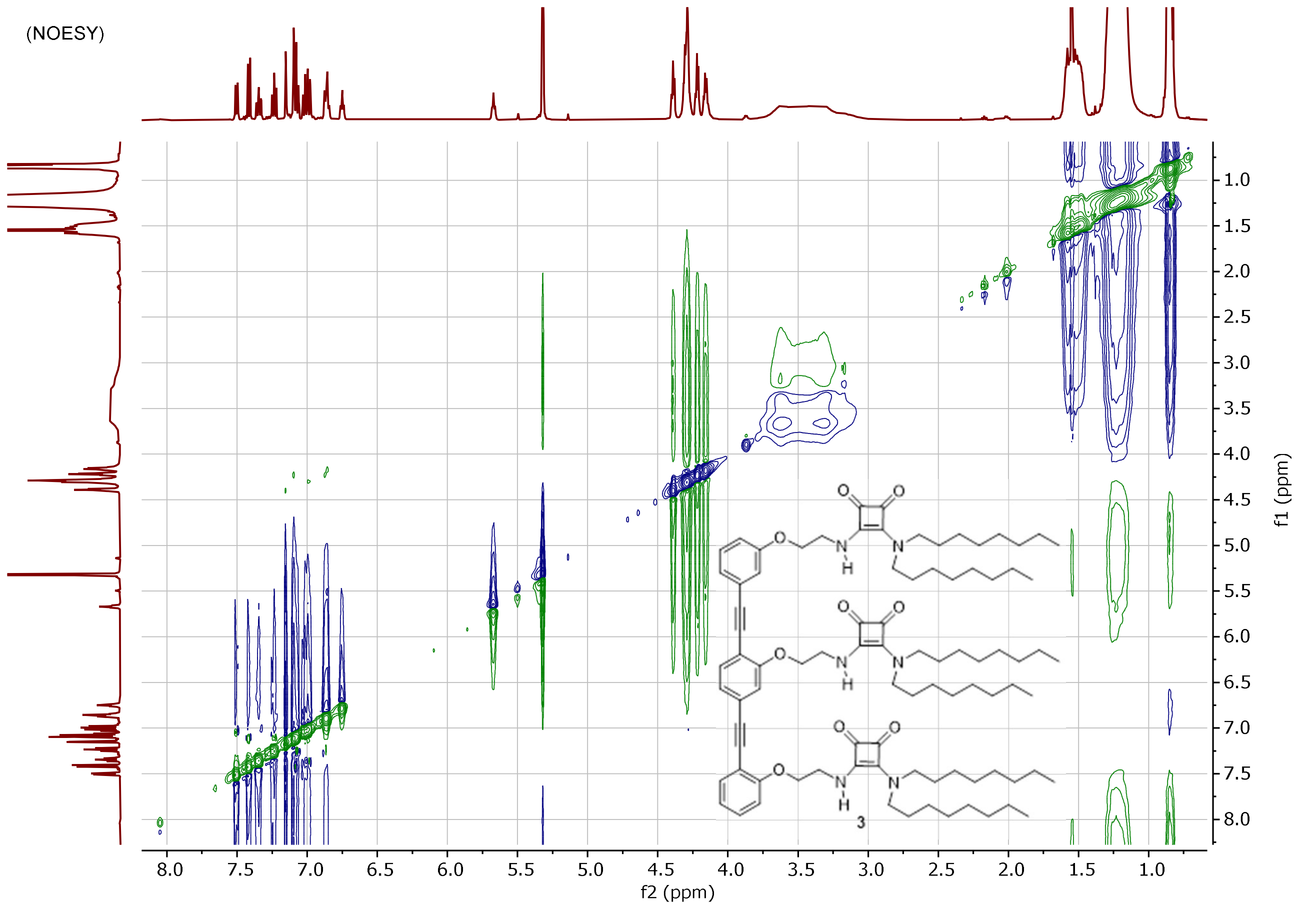
(NOESY)
f1 (ppm)
f2 (ppm)
3

7. References

S1. Fulmer, G. R.; Miller, A. J. M.; Sherden, N. H.; Gottlieb, H. E.; Nudelman, A.; Stoltz, B. M.; Bercaw, J. E.; Goldberg, K. I. NMR Chemical Shifts of Trace Impurities: Common Laboratory Solvents, Organics, and Gases in Deuterated Solvents Relevant to the Organometallic Chemist. *Organometallics* **2010**, *29*, 2176–2179. https://doi.org/10.1021/om100106e.

S2. Martínez-Crespo, L.; Vitórica-Yrezábal, I. J.; Whitehead, G. F. S.; Webb, S. J., Chemically Fueled Communication Along a Scaffolded Nanoscale Array of Squaramides. *Angew. Chem. Int. Ed.* **2023**, *62*, e202307841. https://doi.org/10.1002/anie.202307841

S3. P. Thordarson, P., Determining association constants from titration experiments in supramolecular chemistry. *Chem. Soc. Rev.*, **2011**, *40*, 1305. https://doi.org/10.1039/C0CS00062K

S4. Rigaku Oxford Diffraction, (2024), CrysAlisPro Software system, version 1.171.43, Rigaku Corporation, Wroclaw, Poland

S5. Sheldrick, G. M. Crystal Structure Refinement with SHELXL. *Acta Crystallogr. Sect. C Struct. Chem.* **2015**, *71* (Md), 3–8. https://doi.org/10.1107/S2053229614024218.

S6. Dolomanov, O. V.; Bourhis, L. J.; Gildea, R. J.; Howard, J. A. K.; Puschmann, H. OLEX2: A Complete Structure Solution, Refinement and Analysis Program. *J. Appl. Crystallogr.* **2009**, *42*, 339–341. https://doi.org/10.1107/S0021889808042726.
